# Mediating effect of insulin resistance in the relationship between dietary inflammatory index and cardiovascular–kidney–metabolic syndrome stage in US adults, 2007–2018

**DOI:** 10.29219/fnr.v70.13744

**Published:** 2026-05-20

**Authors:** Ying Xiao, Hongzhao You, Tianjie Wang, Yan Zeng, Yingxuan Zhu, Rui Zhang, Dong Liu, Yanyan Zhao, Yilu Liu, Shijie You, Bokang Qiao, Jiansong Yuan, Weixian Yang

**Affiliations:** 1 Fuwai Hospital, Chinese Academy of Medical Sciences & Peking Union Medical College, National Center for Cardiovascular Diseases, Beijing, China; 2 Beijing Institute of Heart, Lung and Vessel Disease, Capital Medical University Affiliated Anzhen Hospital, Beijing, China

**Keywords:** cardiovascular–kidney–metabolic syndrome, Dietary Inflammatory Index, insulin resistance, dietary intervention

## Abstract

**Background:**

Cardiovascular–kidney–metabolic (CKM) syndrome is a progressive, five-stage disease framework driven by interrelated cardiovascular, renal, and metabolic dysfunction, with chronic inflammation and insulin resistance (IR) playing key roles. The dietary inflammatory index (DII) quantifies overall dietary inflammatory potential, but its role in CKM staging remains unclear.

**Objective:**

This study aimed to evaluate the association between DII and CKM syndrome stage and to assess the potential mediating effect of IR.

**Design:**

This cross-sectional study used data from the National Health and Nutrition Examination Survey (2007–2018). DII was derived from 24-h dietary recall. CKM stages were defined by the American Heart Association criteria. IR was assessed using the triglyceride–glucose index (TyG), Metabolic Score for IR (METS-IR), and Homeostatic Model Assessment for IR (HOMA-IR). Statistical analyses were performed after accounting for the complex survey design.

**Results:**

Of the 27,635 participants, 94.08% had CKM syndrome (Stage 1 or higher). Compared with Stage 0, stepwise increases in the adjusted odds ratios for a higher DII score were observed across CKM stages (Q4 vs. Q1: 1.67 [1.31–2.15] for Stage 1; 2.11 [1.63–2.73] for Stage 2; 2.32 [1.08–4.98] for Stage 3; and 4.19 [2.65–6.63] for Stage 4). Restricted cubic splines models confirmed the positive linear association between DII and each CKM stage (all *P*_overall_ < 0.05; *P*_non-linear_ > 0.05), with increasingly steep slopes at higher stages. TyG, METS-IR, and HOMA-IR mediated 30.7, 45.0, and 25.3% of the association between DII and CKM syndrome stage, respectively.

**Conclusions:**

Pro-inflammatory diet, as indicated by a higher DII score, was positively associated with more advanced CKM syndrome stage, partly mediated by IR. The DII may serve as a practical measure of dietary inflammation in CKM management.

## Popular scientific summary

- A stepwise positive association existed between the Dietary Inflammatory Index (DII) and Cardiovascular–kidney–metabolic (CKM) syndrome stage, and the strength of association increasing across higher CKM stages.
- Insulin resistance (IR) mediated 25–45% of the association between DII and CKM syndrome stage.
- The positive association between higher DII score and advanced CKM syndrome stage was more pronounced in females.
- Clinical implications: A pro-inflammatory diet is associated with increased CKM burden, potentially mediated by IR. The DII is a valuable tool for evaluating dietary inflammatory potential, offering a practical approach for CKM management.

## Central illustration

**Figure:**
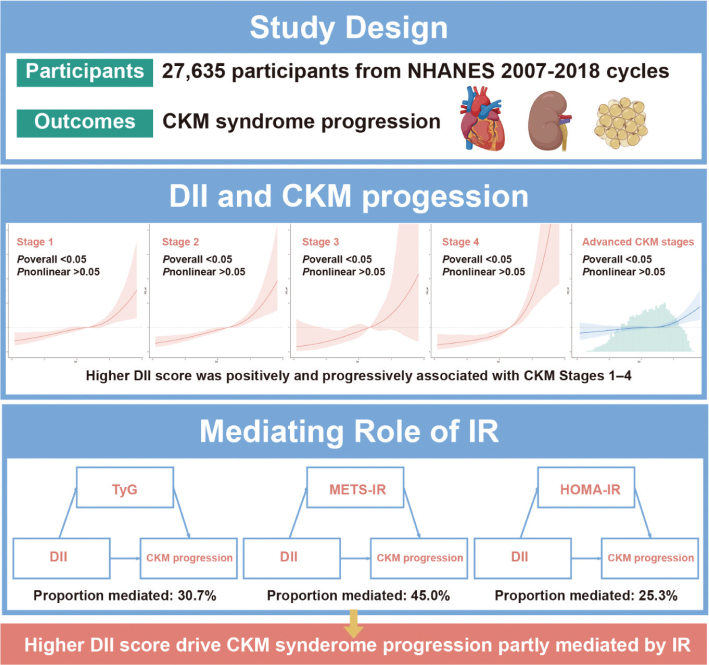
Advanced CKM was defined as Stages 3–4 (high-risk or established CVD). Adjusted for age, race, educational attainment, smoking status, physical activity, and total energy intake.

The complex interplay among metabolic risk factors, chronic kidney disease (CKD), and cardiovascular diseases (CVDs) is becoming increasingly recognized. The American Heart Association (AHA) introduced the concept of cardiovascular–kidney–metabolic (CKM) syndrome in 2023 as a unified disease spectrum (1). The concept stratifies individuals into five progressive stages, representing a continuum from the absence of risk factors (Stage 0) to the presence of excess or dysfunctional adiposity (Stage 1), to metabolic risk factors – such as hypertension, dyslipidemia, and diabetes – along with CKD (Stage 2), followed by subclinical CVD (Stage 3), and culminating in established CVD (Stage 4). The prevalence of CKM is steadily increasing; therefore, it poses a growing public health burden worldwide. Based on data from the 2011–2020 cycles of the National Health and Nutrition Examination Survey (NHANES), nearly 90% of US adults meet the criteria for CKM Stage 1 or higher, and approximately 15% have advanced CKM (Stage 3 or 4) (2). Thus, identifying modifiable risk factors and implementing early strategies to reduce CKM burden have become clinical priorities.

Inflammation and insulin resistance (IR), two closely interrelated mechanisms, are major pathological drivers of CKM progression (1, 3). Chronic systemic inflammation activates multiple inflammatory pathways that disrupt insulin signaling, thereby promoting IR (4). IR not only serves as a fundamental pathological basis for type 2 diabetes mellitus but it is also closely associated with various metabolic disturbances, such as obesity and dyslipidemia, as well as hypertension, atherosclerosis, and renal dysfunction (5, 6), collectively contributing to cardiovascular, renal, and metabolic deterioration. Certain anti-inflammatory approaches have been shown to enhance insulin sensitivity and improve metabolic status, thereby reducing the risk of CKM-associated adverse outcomes (7).

Diet, a core element of Life’s Essential 8 (8), is a key modifiable factor in CKM management. Evidence suggests that healthy dietary patterns, such as the consumption of a Mediterranean diet and the Dietary Approaches to Stop Hypertension diet, confer cardiovascular, renal, and metabolic benefits through shared anti-inflammatory and IR-improving effects (9–11). These findings highlight the pivotal role of optimizing dietary composition in CKM management.

The Dietary Inflammatory Index (DII), a validated composite score calculated based on 45 dietary components, is widely used to quantify the overall inflammatory potential of an individual’s diet (12). Previous research has established positive associations between higher DII score and the incidence of CKM-related diseases, such as obesity, type 2 diabetes mellitus, CVD, and CKD, supporting its potential for guiding nutritional interventions (13–15). However, the association between the DII and CKM syndrome progression across different stages, as well as the mechanistic association between the DII and IR, remains largely undetermined. Addressing these knowledge gaps may provide insights for optimizing dietary strategies for CKM management.

We conducted this large cross-sectional study using data from the 2007–2018 NHANES cycles to systematically evaluate the association between the DII and CKM syndrome stages, and to explore the potential mediating effect of IR.

## Methods

### Study design and population

The NHANES is an ongoing cross-sectional program that uses a complex multistage probability sampling method to collect nationally representative health data from the US population in biennial cycles. The study design and data are publicly available at www.cdc.gov/nchs/nhanes/. Ethical approval was obtained from the Ethics Review Board of the National Center for Health Statistics, and written informed consent was obtained from all participants.

**Fig. 1:**
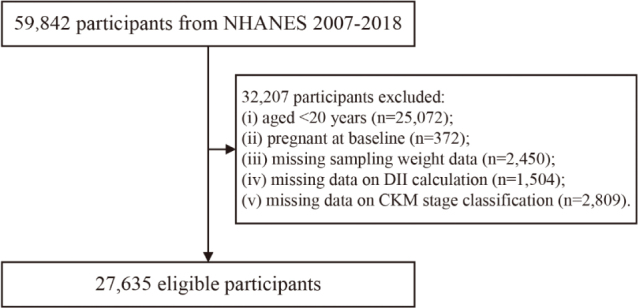
Participant selection flowchart.

We utilized data from six consecutive NHANES cycles (2007–2018), initially including 59,842 participants. Individuals were excluded if they were 1) aged < 20 years (*n* = 25,072), 2) pregnant (*n* = 372), 3) missing sampling weight data (*n* = 2,450), 4) lacking sufficient dietary data to calculate the DII score (*n* = 1,504), or 5) lacking complete information required for CKM staging (*n* = 2,809). Ultimately, 27,635 participants were included in the final analysis (Fig. 1).

## DII assessment

All dietary data for the participants were obtained through standardized 24-h dietary recall interviews. The DII, developed by Shivappa et al., is a well-established tool used to assess the potential inflammatory effects of diet (12). The DII is based on a comprehensive review of 45 dietary parameters and their influence on six key inflammatory biomarkers, including interleukin (IL)-1β, IL-4, IL-6, IL-10, tumor necrosis factor (TNF)-α, and C-reactive protein. The DII score ranges from negative to positive, with positive scores indicating a pro-inflammatory diet and negative scores indicating an anti-inflammatory diet. A higher DII score represents stronger inflammatory potential.

In NHANES, the DII was calculated based on 28 dietary components. Previous studies have shown that fewer than 30 parameters do not compromise the validity of DII (16). Detailed formulas are listed in Supplementary Table 1.

### Assessment of CKM stages

CKM syndrome was classified into five stages based on the 2023 AHA Presidential Advisory Statement (1), with adaptations for use in NHANES (2). The stages were defined as follows: Stage 0: no CKM health risk factors (normal body mass index [BMI] and waist circumference [WC], and not meeting the criteria for a higher stage); Stage 1: excess or dysfunctional adiposity (participants with elevated BMI, WC, or prediabetes); Stage 2: metabolic risk factors and CKD (including hypertriglyceridemia, hypertension, diabetes mellitus, metabolic syndrome, or moderate-to-high-risk CKD as defined by the Kidney Disease – Improving Global Outcomes criteria) (17); Stage 3: subclinical CVD in CKM (participants with high predicted 10-year CVD risk according to the PREVENT equations or with very-high-risk CKD) (18); and Stage 4: clinical CVD in CKM (participants with self-reported CVD). Detailed definitions for the staging of CKM syndrome are provided in Supplementary Tables 2–4.

For analysis, non-advanced CKM was defined as Stages 0–2, and advanced CKM as Stages 3–4, as these stages identify individuals with or at high risk for CVD (2).

### Assessment of surrogate indices of IR

In this study, IR was assessed using three well-validated surrogate indices: triglyceride–glucose index (TyG), Metabolic Score for IR (METS-IR), and Homeostatic Model Assessment for IR (HOMA-IR). These indices were calculated as follows (19):

TyG = Ln [triglycerides (mg/dL) × fasting glucose (mg/dL) ÷ 2];

METS-IR = Ln [2 × fasting glucose (mg/dL) + triglycerides (mg/dL)] × BMI ÷ Ln [high-density lipoprotein cholesterol (HDL-C) (mg/dL)];

HOMA-IR = fasting plasma glucose (mmol/L) × fasting insulin (mIU/L) ÷ 22.5.

### Assessment of other covariates

To control for potential confounders, the following covariates were included: 1) demographic characteristics, including age, sex, race, and educational attainment; 2) lifestyle factors, including smoking status, drinking status, and physical activity level; and 3) dietary factors, including total energy intake (kcal/day) obtained from 24-h dietary recall.

To assess multicollinearity among the covariates, variance inflation factors (VIFs) were calculated, and all VIF values were within acceptable limits (< 5) (Supplementary Fig. 1).

### Statistical analysis

Normally distributed continuous variables are reported as weighted mean ± standard deviation and were compared using weighted analysis of variance. Non-normally distributed continuous variables are expressed as weighted medians with interquartile ranges and were analyzed using the weighted Kruskal–Wallis test. Categorical variables are presented as counts with weighted percentages and were compared using the weighted chi-square test. Weighted correlation analyses were conducted among variables (Supplementary Fig. 1). Missing data were handled using multiple imputation via a multilevel approach tailored for complex survey data (20). The baseline characteristics of the unimputed dataset and information about missing values are summarized in Supplementary Table 5. No significant differences were observed between the datasets before and after imputation.

In the initial exploratory analysis, CKM syndrome stage was treated as an ordinal variable, where higher stages were considered to reflect disease progression. Although ordinal logistic regression was theoretically appropriate, the proportional odds assumption was violated. Therefore, weighted logistic regression was applied to evaluate the associations between the DII score and CKM syndrome, evaluated in two ways: 1) as four separate stages with Stage 0 as the reference and 2) as a binary variable (advanced stages vs. non-advanced stages). Three models were constructed. Model 1 was unadjusted; Model 2 was adjusted for age, sex, and race; and Model 3 was adjusted for the factors in Model 2 plus educational attainment, smoking status, physical activity, and total energy intake. Subgroup analyses were performed by age, sex, race, educational attainment, smoking status, physical activity, and total energy intake. Weighted restricted cubic splines (RCS) regression was used to explore potential non-linear relationships with four knots placed at the 5th, 35th, 65th, and 95th percentiles of the DII distribution.

Structural equation modeling (SEM) was used to quantify the mediating effects of the three IR surrogate indices (TyG, METS-IR, and HOMA-IR). In the SEM framework, path ‘a’ represents the effect of the DII on the IR surrogates, path ‘b’ represents the effect of the IR surrogates on CKM stage, and path ‘c’ represents the direct effect of the DII on CKM syndrome stage. The indirect effect was defined as a × b, while the total effect was defined as c + a × b. The mediated proportion was calculated as a × b ÷ c + a × b.

To thoroughly assess the weighted interactions and joint associations between the DII score and IR surrogates, we further classified the participants into four groups according to the DII (divided by median) and IR surrogates (divided by the inflection point identified in the RCS analysis with CKM syndrome). Additive interactive effects were assessed using relative excess risk due to interaction, proportion attributable to interaction, and synergy index with corresponding 95% confidence intervals (CIs). Multiplicative interaction was assessed by the odds ratio (OR) and 95% CI of the product term. Joint associations were calculated by the OR (95% CI) for CKM syndrome within these groups, with participants with a low DII score and low IR surrogates considered as the reference group.

Finally, sensitivity analyses were performed to evaluate the robustness of the results, including 1) repeating the primary analyses using the complete dataset (*n* = 12,993); 2) recalculating the DII based on the mean intake from two 24-h dietary recalls; 3) excluding Stage 0 participants (*n* = 1,635) and redefining Stage 1–2 as the non-advanced CKM stages; 4) excluding participants with prior genetic conditions (type 1 diabetes mellitus and familial hypercholesterolemia, *n* = 138); and 5) additionally adjusting for the alternate Mediterranean Diet Index (aMD) (21).

All statistical analyses were performed using R software (version 4.4.2), and two-sided *P* < 0.05 was considered statistically significant.

## Results

### Baseline characteristics

Overall, 27,635 participants were included in the final analysis, with a weighted mean age of 48.38 ± 16.92 years and a mean DII score of 0.82 ± 2.05/day. Among them, 13,728 (weighted percentage 49.26%) were male, and 11,572 (66.54%) were non-Hispanic White (Table 1). Compared with those with non-advanced CKM, participants in the advanced stages of CKM were generally older, more likely to be male and of White ethnicity, and exhibited higher values for TyG, METS-IR, and HOMA-IR. They also tended to have lower educational attainment and engaged less frequently in physical activity (all *P* < 0.001) (Supplementary Table 6).

**Table 1:** Weighted baseline characteristics of the participants according to CKM syndrome stages

Characteristic	Total *N* = 27,635	Cardiovascular–kidney–metabolic syndrome stage					*P*
		0 *N* = 1,635	1 *N* = 6,485	2 *N* = 14,707	3 *N* = 1,423	4 *N* = 3,385	
Age, years	48.38 ± 16.92	35.20 ± 13.46	40.17 ± 14.08	49.63 ± 15.05	72.91 ± 9.21	64.76 ± 13.25	< 0.001
Male, %	13,728 (49.26%)	684 (40.56%)	2,889 (46.06%)	7,495 (51.22%)	722 (49.01%)	1,938 (54.47%)	< 0.001
Race, %							< 0.001
Mexican American	4,226 (8.80%)	142 (6.16%)	1,241 (11.78%)	2,365 (8.69%)	151 (4.84%)	327 (5.04%)	
Non-Hispanic White	11,572 (66.54%)	732 (70.25%)	2,477 (62.82%)	5,861 (66.22%)	712 (74.68%)	1,790 (72.08%)	
Non-Hispanic Black	5,795 (10.94%)	266 (8.31%)	1,219 (10.58%)	3,204 (11.16%)	350 (12.21%)	756 (12.32%)	
Other races	6,042 (13.73%)	495 (15.27%)	1,548 (14.82%)	3,277 (13.94%)	210 (8.27%)	512 (10.57%)	
Education, %							< 0.001
Less than high school	6,737 (15.48%)	207 (9.35%)	1,336 (13.54%)	3,654 (15.53%)	446 (21.40%)	1,094 (23.09%)	
High school	6,351 (23.40%)	304 (17.46%)	1,396 (22.62%)	3,421 (23.64%)	358 (26.66%)	872 (27.71%)	
Above high school	14,547 (61.12%)	1,124 (73.19%)	3,753 (63.84%)	7,632 (60.83%)	619 (51.94%)	1,419 (49.19%)	
Current smoker, %	5,516 (19.53%)	339 (18.45%)	1,298 (19.61%)	2,958 (19.68%)	190 (12.72%)	731 (22.01%)	< 0.001
Physical activity, %							< 0.001
Vigorous	5,534 (22.19%)	349 (21.11%)	1,467 (23.97%)	3,123 (23.48%)	138 (10.51%)	457 (15.85%)	
Moderate	5,895 (24.25%)	376 (25.92%)	1,459 (25.09%)	3,137 (24.13%)	271 (22.32%)	652 (22.05%)	
Inactive	16,206 (53.57%)	910 (52.97%)	3,559 (50.95%)	8,447 (52.40%)	1,014 (67.18%)	2,276 (62.10%)	
BMI, kg/m^2^	29.41 ± 6.76	21.84 ± 1.92	29.34 ± 5.43	30.28 ± 7.04	30.02 ± 6.41	30.80 ± 7.28	< 0.001
WC, cm	100.34 ± 16.12	79.89 ± 6.55	98.52 ± 12.78	102.67 ± 16.25	106.00 ± 15.15	106.98 ± 15.84	< 0.001
DII, /day	0.82 ± 2.05	0.43 ± 2.09	0.77 ± 2.07	0.82 ± 2.02	1.03 ± 1.96	1.20 ± 2.05	< 0.001
Energy intake, Kcal/day	2,141.89 ± 970.47	2,218.57 ± 1,039.19	2,188.75 ± 960.17	2,176.73 ± 983.15	1,794.45 ± 703.48	1,900.92 ± 896.67	< 0.001
TG, mg/dL	115.00 (91.03, 142.40)	76.00 (64.35, 87.72)	98.41 (84.75, 109.00)	126.57 (109.28, 151.00)	137.24 (117.77, 166.29)	155.32 (122.00, 184.51)	< 0.001
TC, mg/dL	194.44 ± 41.55	177.45 ± 34.14	191.66 ± 37.34	201.19 ± 42.05	190.12 ± 48.27	180.78 ± 43.79	< 0.001
LDL, mg/dL	115.54 ± 29.42	103.26 ± 23.24	114.76 ± 25.55	120.13 ± 30.29	110.98 ± 32.85	104.39 ± 31.14	< 0.001
HDL, mg/dL	52.97 ± 16.39	61.56 ± 16.17	53.75 ± 15.31	52.00 ± 16.70	51.36 ± 15.17	49.78 ± 15.78	< 0.001
FBG, mg/dL	99.99 (95.00, 106.00)	92.15 (89.36, 95.00)	96.20 (93.18, 100.00)	101.76 (98.00, 108.38)	110.00 (100.74, 156.61)	106.16 (100.51, 154.51)	< 0.001
TyG	8.68 ± 0.52	8.11 ± 0.31	8.39 ± 0.30	8.83 ± 0.49	9.02 ± 0.54	9.05 ± 0.57	< 0.001
METS-IR	44.06 ± 12.41	29.83 ± 3.55	42.46 ± 9.22	46.03 ± 12.82	46.55 ± 12.42	48.27 ± 13.70	< 0.001
HOMA-IR	3.02 (2.08, 4.14)	1.73 (1.15, 2.18)	2.72 (1.96, 3.31)	3.33 (2.38, 4.64)	3.57 (2.48, 7.98)	4.08 (2.87, 8.07)	< 0.001
Scr, mg/dL	0.85 (0.72, 1.00)	0.81 (0.70, 0.94)	0.82 (0.71, 0.96)	0.85 (0.72, 0.99)	1.04 (0.85, 1.32)	0.96 (0.80, 1.18)	< 0.001
eGFR, mL/min/1.73m^2^	95.24 ± 20.99	106.52 ± 16.40	102.59 ± 16.82	95.38 ± 18.68	65.15 ± 23.38	77.29 ± 23.09	< 0.001
UACR, mg/g	6.71 (4.43, 12.19)	6.16 (4.32, 9.03)	5.28 (3.82, 7.84)	7.01 (4.61, 13.45)	17.63 (8.33, 58.33)	11.26 (6.17, 31.88)	< 0.001
Hypertension, %	16,204 (54.37%)	0 (0.00%)	0 (0.00%)	11,933 (81.33%)	1,401 (98.16%)	2,870 (81.65%)	< 0.001
Diabetes, %	5,228 (14.06%)	0 (0.00%)	0 (0.00%)	3,107 (16.39%)	724 (46.84%)	1,397 (37.85%)	< 0.001
CKD, %							< 0.001
Low-risk	22,739 (86.24%)	1,635 (100.00%)	6,485 (100.00%)	12,068 (84.54%)	579 (43.63%)	1,972 (63.97%)	
Moderate-to-high-risk	4,325 (12.54%)	0 (0.00%)	0 (0.00%)	2,639 (15.46%)	565 (40.76%)	1,121 (29.60%)	
Very high-risk	571 (1.22%)	0 (0.00%)	0 (0.00%)	0 (0.00%)	279 (15.61%)	292 (6.43%)	
CVD history	3,385 (9.66%)	0 (0.00%)	0 (0.00%)	0 (0.00%)	0 (0.00%)	3,385 (100.00%)	< 0.001

The data are presented as weighted mean ± standard deviation for normally distributed continuous variables, weighted median (interquartile range) for non-normally distributed continuous variables, and number (weighted percentage) for categorical variables.

The baseline characteristics of the participants according to the CKM syndrome stages are summarized in Table 1. CKM Stage 2 comprised the largest proportion of the study population (14,707 participants, 52.54%). The DII score exhibited a significant upward trend in parallel with CKM stage progression (Stages 0–4: 0.43 ± 2.09, 0.77 ± 2.07, 0.82 ± 2.02, 1.03 ± 1.96, and 1.20 ± 2.05, respectively, *P*_trend_ < 0.001). In addition, the prevalence of advanced CKM also increased steadily across ascending DII quartiles (Q1–Q4: 10.98, 12.39, 14.14and 16.42%, respectively; *P*_trend_ < 0.001) (Supplementary Table 7).

### Association between the DII score and CKM syndrome stage

As shown in Table 2, the weighted logistic regression analysis revealed a positive association between the DII score and CKM Stages 1–4 (Stage 0 as the reference group), both when analyzed as continuous and categorical variables. This association remained statistically significant after adjustment for age, sex, race, and other potential confounders (all *P* < 0.05). Notably, the strength of the association between the DII score and CKM stage increased progressively with advancing CKM stages. Comparing the highest to the lowest DII quartiles, a stepwise increase in the adjusted OR and 95% CI for CKM syndrome was observed across stages (1.67 [1.31–2.15] for Stage 1, 2.11 [1.63–2.73] for Stage 2, 2.32 [1.08–4.98] for Stage 3, and 4.19 [2.65–6.63] for Stage 4).

**Table 2:** Weighted logistic regression analysis of the association between the DII and CKM syndrome stages

	DII	Stage 1	Stage 2	Stage 3	Stage 4	Advanced stages
Model 1^a^	Continuous	1.08 (1.04, 1.12)	1.10 (1.06, 1.13)	1.15 (1.10, 1.21)	1.19 (1.15, 1.24)	1.10 (1.07, 1.13)
	Q1 (< −0.69)	Reference	Reference	Reference	Reference	Reference
	Q2 (−0.69 to 0.99)	1.08 (0.88, 1.34)	1.27 (1.02, 1.58)	1.44 (1.08, 1.93)	1.32 (1.07, 1.63)	1.15 (0.97, 1.35)
	Q3 (0.99–2.42)	1.24 (0.99, 1.56)	1.33 (1.07, 1.65)	1.54 (1.12, 2.11)	1.75 (1.37, 2.25)	1.33 (1.13, 1.57)
	Q4 (≥ 2.42)	1.42 (1.17, 1.72)	1.56 (1.31, 1.87)	1.99 (1.52, 2.61)	2.46 (1.96, 3.10)	1.59 (1.38, 1.84)
	*P* for trend	< 0.001	< 0.001	< 0.001	< 0.001	< 0.001
Model 2^b^	Continuous	1.12 (1.08, 1.16)	1.15 (1.11, 1.19)	1.33 (1.18, 1.51)	1.36 (1.27, 1.46)	1.11 (1.08, 1.15)
	Q1 (<−0.69)	Reference	Reference	Reference	Reference	Reference
	Q2 (−0.69 to 0.99)	1.16 (0.94, 1.44)	1.32 (1.03, 1.70)	1.58 (0.75, 3.33)	1.66 (1.13, 2.44)	1.17 (0.98, 1.39)
	Q3 (0.99–2.42)	1.43 (1.13, 1.80)	1.58 (1.24, 2.00)	2.62 (1.32, 5.21)	3.38 (2.20, 5.18)	1.40 (1.16, 1.68)
	Q4 (≥ 2.42)	1.72 (1.38, 2.13)	2.02 (1.62, 2.50)	3.60 (1.90, 6.83)	4.84 (3.31, 7.07)	1.71 (1.47, 1.99)
	*P* for trend	< 0.001	< 0.001	< 0.001	< 0.001	< 0.001
Model 3^c^	Continuous	1.12 (1.07, 1.18)	1.17 (1.12, 1.23)	1.23 (1.05, 1.43)	1.34 (1.23, 1.47)	1.06 (1.02, 1.09)
	Q1 (< −0.69)	Reference	Reference	Reference	Reference	Reference
	Q2 (−0.69 to 0.99)	1.16 (0.93, 1.44)	1.36 (1.05, 1.76)	1.35 (0.63, 2.89)	1.62 (1.12, 2.35)	1.05 (0.88, 1.25)
	Q3 (0.99–2.42)	1.41 (1.09, 1.83)	1.63 (1.25, 2.13)	1.83 (0.88, 3.83)	3.06 (1.93, 4.85)	1.15 (0.95, 1.40)
	Q4 (≥ 2.42)	1.67 (1.31, 2.15)	2.11 (1.63, 2.73)	2.32 (1.08, 4.98)	4.19 (2.65, 6.63)	1.28 (1.07, 1.51)
	*P* for trend	< 0.001	< 0.001	0.032	< 0.001	0.005

a Unadjusted.

b Adjusted for age, sex, and race.

c Adjusted for age, sex, race, educational attainment, smoking status, physical activity, and total energy intake.

Data are presented as ORs (95% CIs). Advanced CKM was defined as Stages 3–4 (high-risk or established CVD).

**Fig. 2:**
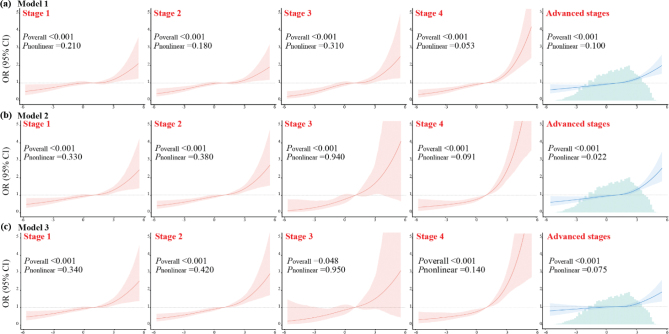
Weighted restricted cubic splines regression model for the DII across different CKM syndrome stages. (a) Unadjusted; (b) Adjusted for age, sex, and race; (c) Adjusted for age, sex, race, educational attainment, smoking status, physical activity, and total energy intake. Advanced CKM was defined as Stages 3–4 (high-risk or established CVD).

Furthermore, weighted RCS regression models identified a positive linear relationship between the DII score and each CKM stage (all *P*_overall_ < 0.05; *P*_non-linear_ > 0.05) (Fig. 2). In addition, as CKM stage increased, the RCS curve slope progressively steepened. Specifically, each 1 unit increase in the DII score was associated with a 12, 17, 23, and 34% increased risk of CKM Stages 1–4, respectively (all *P* < 0.001) (Table 2).

When dichotomizing the CKM stages into advanced versus non-advanced groups, a similar positive exposure–response association was observed between the DII score and CKM syndrome. Elevated DII score was linearly associated with an increased risk of advanced CKM (*P*_overall_ < 0.001; *P*_non-linear_ = 0.075), with each 1 unit increase in the DII score associated with an approximately 6% increased risk of advanced CKM syndrome (adjusted OR [95% CI]: 1.06 [1.02–1.09]) (Fig. 2, Table 2).

### Mediating effect of IR in the DII–CKM association

Using SEM, we evaluated the mediating roles of the surrogate IR indices (TyG, METS-IR, and HOMA-IR) in the association between the DII score and CKM stages. The estimated indirect effect (path a × b), direct effect (path c), and total effect (path c + a × b) were all statistically significant (*P* < 0.05). The detailed results are presented in Supplementary Tables 8–10.

**Fig. 3:**
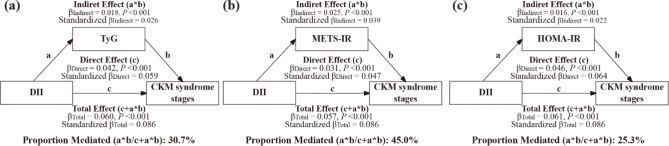
Mediation effects of TyG, METS-IR, and HOMA-IR between the DII and CKM syndrome stages. (a) Mediation effects of TyG; (b) Mediation effects of METS-IR; (c) Mediation effects of HOMA-IR. Adjusted for age, sex, race, educational attainment, smoking status, physical activity, and total energy intake.

As shown in Fig. 3, after adjusting for potential confounders, TyG, METS-IR, and HOMA-IR accounted for 30.7, 45.0, and 25.3% of the total effect of the DII score on higher CKM syndrome stage, respectively.

Moreover, when the CKM stage was dichotomized into categories, the mediation effects of IR indices remained significant, with an adjusted mediation proportion of 23.5% for TyG, 34.1% for METS-IR, and 54.2% for HOMA-IR (Supplementary Figs 11–13).

### Interaction and joint analysis of the DII score and IR with CKM syndrome stage

Weighted RCS regression models demonstrated a positive linear association between the DII score and IR surrogates (*P*_overall_ < 0.05; *P*_non-linear_ > 0.05) (Supplementary Fig. 2), whereas a non-linear relationship was observed between the IR surrogates and advanced CKM stages (*P*_overall_ < 0.05; *P*_non-linear_ < 0.05) (Supplementary Fig. 3). Based on these findings, the participants were stratified according to the inflection points of the IR surrogates identified from the RCS curves (8.31 for TyG, 42.18 for METS-IR, and 1.33 for HOMA-IR; Supplementary Fig. 3) alongside a median split in the DII score (0.99/day). These categorizations were subsequently applied to assess additive or multiplicative interactions and joint associations between the DII score and IR in relation to CKM syndrome stage.

No significant multiplicative or additive interactions were observed between the DII score and TyG, METS-IR, or HOMA-IR in relation to advanced CKM stages (Supplementary Tables 14–16). Supplementary Figs 4–6 illustrate the joint effect of the DII score and TyG, METS-IR, and HOMA-IR on CKM syndrome, revealing that participants with both a high DII score and high IR surrogates exhibited the greatest risk of advanced CKM (*P*_trend_ < 0.05).

### Subgroup and sensitivity analyses

**Fig. 4:**
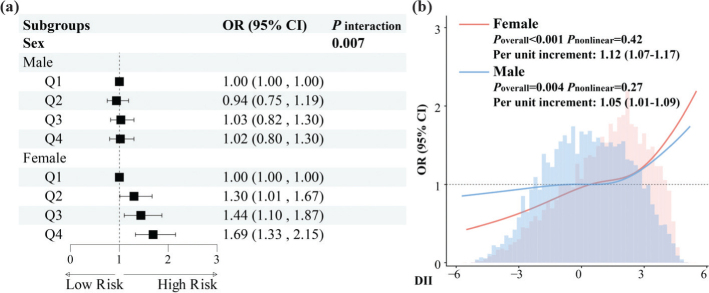
Sex-specific associations between DII and advanced CKM syndrome stages. (a) Sex-specific subgroup analysis of the DII in quartiles and advanced CKM syndrome stages; (b) Sex-stratified weighted restricted cubic spline regression model of the relationship between the DII and advanced CKM syndrome stages.

In the subgroup analyses (Fig. 4, Supplementary Figs 7–8), a significant interaction between the DII score and sex was observed (*P*_interaction_ < 0.05). When DII was analyzed in quartiles, females in the highest DII quartile had a significantly higher risk of advanced CKM compared to those in the lowest quartile (adjusted OR [95% CI]: 1.69 [1.33–2.15]), whereas no significant association was found in males (Fig. 4a). When analyzed as a continuous variable, each unit increase in DII was significantly associated with advanced CKM stages in both sexes, with a stronger association observed in females (adjusted OR [95% CI]: 1.12 [1.07–1.17] in females, 1.05 [1.01–1.09] in males).

This sex-specific difference was further explored through sex-stratified weighted RCS regression models (Fig. 4b). RCS analysis revealed a more pronounced positive relationship between DII and CKM stage in females, while the slope in males was weaker and more gradual, though still positive.

In the sensitivity analyses, the positive association between the DII score and advanced CKM syndrome stages, as well as the mediating roles of the IR surrogates, remained robust after conducting complete-case analyses (Supplementary Tables 17–21), recalculating the DII using the mean dietary intake from two 24-h recall interviews (Supplementary Tables 22–26), excluding Stage 0 participants (Supplementary Tables 27–28), excluding participants with prior genetic conditions (Supplementary Tables 29–30), and further adjusting for the aMD (Supplementary Fig. 9, Supplementary Tables 31–33).

## Discussion

In a nationally representative sample of US adults, we found that a more pro-inflammatory diet, as indicated by a higher DII score, was positively and progressively associated with CKM Stages 1–4. In addition, subgroup analyses indicated that this positive association was more pronounced among female participants. Notably, this is the first study to demonstrate that IR partially mediates the positive relationship between the DII score and CKM syndrome stage. These findings suggest that a higher DII score is associated with advanced CKM stages, highlighting the potential of early dietary intervention for reducing CKM burden.

### Dietary inflammation and CKM syndrome stage

CKM syndrome is a complex, progressive condition that originates from excess or dysfunctional adipose tissue. This dysfunctional fat accumulation triggers key pathophysiological processes, including chronic inflammation, oxidative stress, and IR, which contribute to metabolic disturbances and CKD onset. As the disease advances, overlapping comorbidities may lead to subclinical coronary artery calcification, myocardial structural and functional abnormalities, and persistent renal impairment, ultimately culminating in cardiovascular events and end-stage kidney disease (1, 3). The CKM staging model captures this continuum of pathological progression and the stepwise increase in adverse outcome risk. Each incremental increase in CKM stage is associated with a 22% increase in all-cause mortality, a 37% increase in cardiovascular mortality, and an approximate 3-year reduction in life expectancy (22). Therefore, the early identification of high-risk individuals with CKM progression and timely intervention may offer significant clinical benefits.

Among the various early intervention strategies, dietary management is widely recognized as a cornerstone for improving the health of individuals with CKM. Moreover, chronic low-grade inflammation has been identified in recent guidelines as a key risk-enhancing factor for CKM development (1). In this context, increasing attention has been directed toward the role of diet-induced inflammation. Different dietary components can induce varying degrees of systemic inflammatory responses, thereby influencing disease risk (23). For instance, anti-inflammatory foods, such as fruit and vegetables, are associated with reduced risk of cardiovascular events (24), while pro-inflammatory foods, such as processed meats and refined carbohydrates, are linked to increased CVD risk (25). Thus, there is a need to quantify the overall inflammatory potential of an individual’s diet, with the goal of providing practical and actionable tools to inform nutritional strategies.

To address this, Shivappa et al. developed the DII, a scoring algorithm based on the relationship between 45 dietary components and inflammatory biomarkers (12). DII has been widely validated in large-scale studies, and its predictive value for metabolic disorders, CVD, and CKD has been demonstrated previously (13–15). Moreover, two early studies attempted to integrate cardiometabolic syndrome and CKD as a composite phenotype to reflect multisystem injury, observing a positive association with the DII (26, 27).

However, treating cardiovascular, renal, and metabolic impairments as independent outcomes or analyzing them through simple aggregation fails to capture their synergistic progression. For instance, a nationally representative US study reported 10-year mortality rates of 11.5% for CKD and 7.7% for diabetes mellitus, but this risk increased sharply to 31.1% among individuals with comorbid CKD and diabetes mellitus (28), suggesting a substantial synergistic effect beyond additive risk.

Therefore, in this study, firstly based on the AHA-defined CKM staging model, we observed a significant positive association between the DII score and CKM stage, with the strength of this association gradually increasing with more advanced stages of CKM, thereby highlighting that the DII score is a promising factor related to CKM stage severity. Dietary modification represents a modifiable therapeutic strategy, and the DII offers a practical tool for quantifying dietary inflammation in CKM management.

### The mediating effect of IR

The mediation analysis in our study revealed that IR accounted for approximately 25–45% of the association between the DII score and CKM staging, indicating a substantial mediating effect.

The mediating effects of the three IR surrogate indices varied in this study. HOMA-IR, a classic marker of IR based on fasting insulin and glucose, exhibited the weakest mediating effect (adjusted mediation proportion: 25.3%). This may be attributed to its high variability owing to technical issues in insulin measurement and the sensitivity to demographic and metabolic factors, limiting its clinical reliability. The TyG, calculated from fasting triglycerides and glucose, showed a stronger mediating effect (30.7%) than HOMA-IR. The TyG more effectively captures systemic abnormalities in glucose and lipid metabolism and offers greater stability and feasibility in clinical practice (29). Among the three IR surrogates, METS-IR demonstrated the strongest mediating effect (45.0%). By further incorporating BMI and HDL-C into its calculation, METS-IR provides a more comprehensive assessment of adiposity and lipid status than the TyG. Previous studies have shown that METS-IR outperforms HOMA-IR and TyG in predicting CKD, type 2 diabetes mellitus, and cardiovascular mortality (19, 30, 31). Our findings suggest that METS-IR may serve as a more integrated and sensitive marker of IR in CKM syndrome, warranting investigation in future research.

Mechanistically, the consumption of a pro-inflammatory diet is strongly linked to IR through multiple pathways. Firstly, a pro-inflammatory diet can upregulate pro-inflammatory cytokines, such as IL-6, IL-1β, and TNF-α (12); activate signaling pathways, such as c-Jun N-terminal kinase and inhibitor of kappa B kinase; and induce serine phosphorylation of insulin receptor substrate 1, thereby impairing insulin receptor binding and downstream signaling (4, 32). Secondly, specific dietary components, such as saturated fat, may directly disrupt hepatic gene expression and cellular signaling, promoting IR (33). Moreover, excess intake of energy-dense foods, such as high-fat and high-sugar foods, may activate the mTOR/S6K1 pathway via aldosterone and angiotensin II signaling, leading to IR in cardiovascular and renal tissues (34). Chronic systemic inflammation and IR synergistically impair endothelial function, reduce vascular elasticity, and activate the renin–angiotensin–aldosterone system (RAAS), thereby promoting vasoconstriction, tissue fibrosis, and organ damage, collectively accelerating CKM pathophysiology (3). Our findings support the potential pathogenic role of diet-induced inflammation in CKM progression and highlight its mechanistic link to IR, offering new evidence for targeted dietary interventions.

### Sex differences in DII and CKM

Our study revealed significant sex-specific differences in the association between the DII score and advanced CKM syndrome. A sharper positive association was observed among females, whereas males exhibited a weaker, more gradual yet still positive relationship. While the underlying mechanisms remain unclear, these sex differences could be influenced by variations in dietary habits, inflammatory responses, and sex hormones. Of particular note, the average age of participants in our study was over 40 years, with 52.04% of the female participants being postmenopausal. The decline in estrogen levels post-menopause is known to diminish its protective effects against inflammation and IR, potentially accelerating the inflammation-driven progression of CKM. Furthermore, estrogen deficiency may negatively impact lipid metabolism, endothelial function, and RAAS activation, thus exacerbating damage along the CKM pathway (35, 36). These findings highlight the importance of incorporating sex stratifications in future studies to better understand the biological mechanisms driving these sex-specific associations and their implications for CKM management.

### Strengths and limitations

This study has several prominent strengths. To our knowledge, this is the first study to systematically evaluate the association between the DII score and CKM syndrome across five progressive AHA stages in a large, nationally representative US sample. Importantly, we also identified IR as a partial mediator of this relationship. Comprehensive sensitivity analyses confirmed the robustness of our findings.

Several limitations should also be acknowledged. Firstly, owing to the cross-sectional design of this study, causal inferences could not be made. Secondly, the DII score was calculated using 24-h dietary recall, which may have introduced recall bias and affected the measurement accuracy. Thirdly, as the study sample was derived from the NHANES in the US, the generalizability of the findings to other regions may be limited. Finally, although we used multiple imputation to address missing data, some variables had relatively high rates of missingness, which may have affected the imputation quality. However, the consistency between the complete and imputed datasets supports the robustness of our conclusions.

### Implications for practice and research

Our study carries important implications for clinical practice and future research. Firstly, we identified pro-inflammatory dietary patterns – quantified by the DII score – as promising, modifiable risk factors for more advanced CKM syndrome stages. Furthermore, the dynamically increasing strength of the positive association between the DII score and CKM staging underscores that reducing dietary inflammatory load should be a key target in optimizing nutritional interventions across the CKM spectrum. Notably, our mediation analysis revealed that IR may mediate a substantial proportion of the positive relationship between the DII score and CKM stage, advocating that chronic inflammation may drive CKM pathophysiology partially by disrupting insulin signaling pathways. In addition, our finding of a sex-specific association between the DII score and advanced CKM implies that future research should consider sex-based differences when developing DII-relevant management strategies.

## Conclusion

Based on our findings, we propose that pro-inflammatory dietary patterns, reflected by higher DII scores, may simultaneously promote chronic inflammation and exacerbate IR, jointly contributing to more advanced CKM syndrome stages. This framework provides a novel perspective on how diet-induced inflammation may contribute to the integrated deterioration of cardiovascular, renal, and metabolic systems, offering a rationale for future mechanistic studies and targeted dietary interventions.

## Supplementary Material



## Declarations

### Availability of data and materials

All relevant data in this study are publicly available in National Health and Nutrition Examination Survey Homepage (https://www.cdc.gov/nchs/nhanes/index.htm).
