# Supplementary material for: Mediating effect of insulin resistance in the relationship between dietary inflammatory index and cardiovascular–kidney–metabolic syndrome stage in US adults, 2007–2018
Source: Food Nutr Res. 2026 May 20;70:10.29219/fnr.v70.13744. doi: 10.29219/fnr.v70.13744 (PMC13224937; doi:10.29219/fnr.v70.13744)
Supplement: Supplementary file 1 [file FNR-70-13744-s1.docx]

**Supplementary Material**

**Item**

**Tables**

Table S1 Detailed formulas and component-specific parameters used in the DII calculation.

Table S2 Definitions of CKM conditions.

Table S3 The basic PREVENT 10-year risk estimation model equations.

Table S4 Detailed definitions of CKM staging in NHANES.

Table S5 Weighted baseline characteristics of participants according to Cardiovascular-Kidney-Metabolic syndrome stages (unimputed dataset).

Table S6 Weighted baseline characteristics of participants according to advanced Cardiovascular-Kidney-Metabolic syndrome stages.

Table S7 Weighted baseline characteristics of participants according to the quartile level of dietary inflammatory index.

Table S8 Mediation effects of TyG between dietary inflammatory index and Cardiovascular-Kidney-Metabolic syndrome stages.

Table S9 Mediation effects of METS-IR between dietary inflammatory index and Cardiovascular-Kidney-Metabolic syndrome stages.

Table S10 Mediation effects of HOMA-IR between dietary inflammatory index and Cardiovascular-Kidney-Metabolic syndrome stages.

Table S11 Mediation effects of TyG between dietary inflammatory index and advanced Cardiovascular-Kidney-Metabolic syndrome stages.

Table S12 Mediation effects of METS-IR between dietary inflammatory index and advanced Cardiovascular-Kidney-Metabolic syndrome stages.

Table S13 Mediation effects of HOMA-IR between dietary inflammatory index and advanced Cardiovascular-Kidney-Metabolic syndrome stages.

Table S14 Weighted interactive effects of dietary inflammatory index and TyG on advanced Cardiovascular-Kidney-Metabolic syndrome stages.

Table S15 Weighted interactive effects of dietary inflammatory index and METS-IR on advanced Cardiovascular-Kidney-Metabolic syndrome stages.

Table S16 Weighted interactive effects of dietary inflammatory index and HOMA-IR on advanced Cardiovascular-Kidney-Metabolic syndrome stages.

Table S17 Weighted baseline characteristics of participants according to Cardiovascular-Kidney-Metabolic syndrome stages (complete dataset).

Table 18 Weighted logistic regression analysis for the association between dietary inflammatory index and Cardiovascular-Kidney-Metabolic syndrome stages (complete dataset).

Table S19 Mediation effects of TyG between dietary inflammatory index and Cardiovascular-Kidney-Metabolic syndrome stages (complete dataset).

Table S20 Mediation effects of METS-IR between dietary inflammatory index and Cardiovascular-Kidney-Metabolic syndrome stages (complete dataset).

Table S21 Mediation effects of HOMA-IR between dietary inflammatory index and Cardiovascular-Kidney-Metabolic syndrome stages (complete dataset).

Table S22 Weighted baseline characteristics of participants according to Cardiovascular-Kidney-Metabolic syndrome stages (Dietary inflammatory index recalculated dataset).

Table S23 Weighted logistic regression analysis for the association between dietary inflammatory index and Cardiovascular-Kidney-Metabolic syndrome stages (Dietary inflammatory index recalculated dataset).

Table S24 Mediation effects of TyG between dietary inflammatory index and Cardiovascular-Kidney-Metabolic syndrome stages (Dietary inflammatory index recalculated dataset).

Table S25 Mediation effects of METS-IR between dietary inflammatory index and Cardiovascular-Kidney-Metabolic syndrome stages (Dietary inflammatory index recalculated dataset).

Table S26 Mediation effects of HOMA-IR between dietary inflammatory index and Cardiovascular-Kidney-Metabolic syndrome stages (Dietary inflammatory index recalculated dataset).

Table S27 Weighted logistic regression analysis for the association between dietary inflammatory index and Redefined Advanced Cardiovascular-Kidney-Metabolic syndrome stages (Excluding Stage 0 participants).

Table S28 Mediation effects of IR surrogates between dietary inflammatory index and Redefined Advanced Cardiovascular-Kidney-Metabolic syndrome stages (Excluding Stage 0 participants).

Table S29 Weighted logistic regression analysis for the association between dietary inflammatory index and Cardiovascular-Kidney-Metabolic syndrome stages (Excluding participants with prior genetic conditions).

Table S30 Mediation effects of IR surrogates between dietary inflammatory index and Redefined Advanced Cardiovascular-Kidney-Metabolic syndrome stages (Excluding participants with prior genetic conditions).

Table S31 Alternate Mediterranean Diet Index components and criteria for scoring.

Table S32 Weighted logistic regression analysis for the association between dietary inflammatory index and Cardiovascular-Kidney-Metabolic syndrome stages (Further adjusting for aMD).

**Figures**

Fig. S1 Weighted correlation matrix and variance inflation factor of covariates.

Fig. S2 Weighted RCS regression model for dietary inflammatory index with TyG, METS-IR, and HOMA-IR.

Fig. S3 Weighted RCS regression model for TyG, METS-IR, and HOMA-IR across different Cardiovascular-Kidney-Metabolic syndrome stages.

Fig. S4 Weighted joint effects of dietary inflammatory index and TyG on advanced Cardiovascular-Kidney-Metabolic syndrome stages.

Fig. S5 Weighted joint effects of dietary inflammatory index and METS-IR on advanced Cardiovascular-Kidney-Metabolic syndrome stages.

Fig. S6 Weighted joint effects of dietary inflammatory index and HOMA-IR on advanced Cardiovascular-Kidney-Metabolic syndrome stages.

Fig.S7. Age subgroup analysis of the association between dietary inflammatory index and advanced Cardiovascular-Kidney-Metabolic syndrome stages.

Fig.S8 Subgroup analysis of the association between dietary inflammatory index and advanced Cardiovascular-Kidney-Metabolic syndrome stages.

Fig. S9. Subgroup analysis by aMD of the association between the DII and advanced CKM syndrome stages.

**Table S1** **Detailed formulas and component-specific parameters used in the DII calculation.**

| Food parameter | Overall inflammatory effect score | Global daily mean intake (units/d) | standard deviation |
| --- | --- | --- | --- |
| Alcohol (g) | -0.278 | 13.98 | 3.72 |
| Vitamin A (RE) | -0.401 | 983.9 | 518.6 |
| Vitamin B6 (mg) | -0.365 | 1.47 | 0.74 |
| Vitamin B12 (μg) | 0.106 | 5.15 | 2.7 |
| Vitamin C (mg) | -0.424 | 118.2 | 43.46 |
| Vitamin D (μg) | -0.446 | 6.26 | 2.21 |
| Vitamin E (mg) | -0.419 | 8.73 | 1.49 |
| Thiamin (mg) | -0.098 | 1.7 | 0.66 |
| Riboflavin (mg) | -0.068 | 1.7 | 0.79 |
| Beta-carotene (μg) | -0.584 | 3718 | 1720 |
| Caffeine (g) | -0.11 | 8.05 | 6.67 |
| Carbohydrate (g) | 0.097 | 272.2 | 40 |
| Cholesterol (mg) | 0.11 | 279.4 | 51.2 |
| Energy (kcal) | 0.18 | 2056 | 338 |
| Total fat (g) | 0.298 | 71.4 | 19.4 |
| Fiber (g) | -0.663 | 18.8 | 4.9 |
| Folate (μg) | -0.19 | 273 | 70.7 |
| Iron (mg) | 0.032 | 13.35 | 3.71 |
| Magnesium (mg) | -0.484 | 310.1 | 139.4 |
| Monounsaturated fatty acids (g) | -0.009 | 27 | 6.1 |
| Polyunsaturated fatty acids (g) | -0.337 | 13.88 | 3.76 |
| n-3 Fatty acids (g) | -0.436 | 1.06 | 1.06 |
| n-6 Fatty acids (g) | -0.159 | 10.8 | 7.5 |
| Niacin (mg) | -0.246 | 25.9 | 11.77 |
| Protein (g) | 0.021 | 79.4 | 13.9 |
| Saturated fat (g) | 0.373 | 28.6 | 8 |
| Selenium (μg) | -0.191 | 67 | 25.1 |
| Zinc (mg) | -0.313 | 9.84 | 2.19 |

The formula of DII calculation:

1. For each nutrient: $Z score= \frac{daily mean intake-global daily mean intake}{standard deviation}$;
2. Conversion to centered percentile score: $centered percentile score=2*percentile of Z score-1$;
3. Nutrient-specific DII score: $DII score=centered percentile score*inflammatory effect score$;
4. Overall DII score: $overall DII score= \sum DII score for each nutrient component$.

**Table S2.** **Definitions of CKM conditions.**

| CKM conditions | CKM indicators | Definitions |
| --- | --- | --- |
| CVD | Clinical CVD | History of chronic heart failure, coronary heart disease, angina, heart attack, or stroke |
|  | Subclinical CVD | Any of the following criterion is met:  1) Very high-risk CKD in KDIGO classification: UACR ≥ 300 mg/g and eGFR ≤ 45-59 ml/min/1.73m^2^, UACR ≥ 30 mg/g and eGFR ≤ 30-44 ml/min/1.73m^2^, or eGFR ≤ 29 ml/min/1.73m^2^  2) Predicted 10-year CVD risk ≥ 20% in PREVENT equations |
| Kidney diseases | CKD | Moderate-to-high-risk CKD in KDIGO classification: UACR ≥ 30 mg/g and eGFR ≥ 60 ml/min/1.73m^2^, UACR < 300 mg/g and eGFR ≤ 45-59 ml/min/1.73m^2^, or UACR < 30 mg/g and eGFR ≤ 30-44 ml/min/1.73m^2^. |
| Metabolic disorders | Overweight/obesity | BMI ≥25 kg/m2 (or ≥23 kg/m2 if Asian ancestry)* |
|  | Abdominal obesity | Waist circumference ≥88/102 cm in female/male (or if Asian ancestry ≥80/90 cm in female/male) |
|  | Prediabetes | Fasting blood glucose ≥ 100-124 mg/dL or HbA1c ≥ 5.7%-6.4% and without self-reported diagnosis of diabetes, use of insulin, or oral hypoglycemic agents |
|  | Diabetes | Fasting blood glucose ≥ 125 mg/dL or HbA1c ≥ 6.5% or self-reported diagnosis of diabetes, use of insulin, or oral hypoglycemic agents |
|  | Hypertension | SBP ≥130 mm Hg or DBP ≥80 mmHg or self-reported diagnosis of hypertension or use of antihypertensive medications |
|  | Hypertriglyceridemia | Triglycerides ≥ 135 mg/dL |
|  | MetS | MetS is defined by the presence of 3 or more of the following:  1) Waist circumference ≥88/102 cm in female/male (or if Asian ancestry ≥80/90 cm in female/male).  2) HDL cholesterol ≥50/40 mg/dL in female/male.  3) Triglycerides ≥150 mg/dL.  4) Elevated blood pressure (SBP ≥130 mmHg or DBP ≥80 mmHg and/or use of antihypertensive medications)  5) Fasting blood glucose ≥100 mg/dL |

Abbreviations: CKM, cardiovascular-kidney-metabolic; CVD, cardiovascular disease; CKD, chronic kidney disease; KDIGO, The Kidney Disease: Improving Global Outcomes; UACR, urinary albumin to creatinine ratio; eGFR, estimated glomerular filtration rate; BMI, body mass index; SBP, systolic blood pressure; DBP, diastolic blood pressure; MetS, metabolic syndrome.

*Asian was not listed as a separate race/ethnicity until NAHNES 2011-2012, therefore the uniform threshold for BMI and waist circumference was used in all participants in NHANES 2007-2010.

**Table S3.** **The basic PREVENT 10-year risk estimation model equations.**

| 10-year CVD risk assessment equation | |
| --- | --- |
| Men | log-Odds = -3.031168 + 0.7688528 × (age – 55) /10 + 0.0736174 × ((TC – HDL-C) × 0.02586 – 3.5) – 0.0954431 × (HDL-C × 0.02586 – 1.3) /0.3 – 0.4347345 × (min(SBP, 110) – 110) /20 + 0.3362658 × (max(SBP, 110) – 130) /20 + 0.7692857 × (if diabetes) + 0.4386871 × (if current smoker) + 0.5378979 × (min(eGFR, 60) – 60) / -15 + 0.0164827 × (max(eGFR, 60) – 90) / -15 + 0.288879 × (if using anti-hypertensive medication) – 0.1337349 × (if using statin) – 0.0475924 × (if using anti-hypertensive medication) × (max(SBP, 110) – 130) /20 + 0.150273 × (if using statin) × ((TC – HDL-C) × 0.02586 – 3.5) – 0.0517874 × (age – 55) /10 × ((TC – HDL-C) × 0.02586 – 3.5) + 0.0191169 × (age – 55) /10 × (HDL-C × 0.02586 – 1.3) /0.3 – 0.1049477 × (age – 55) /10 × (max(SBP, 110) – 130) /20 – 0.2251948 × (age – 55) /10 × (if diabetes) – 0.0895067 × (age – 55) /10 × (if current smoker) – 0.1543702 × (age – 55) /10 × (min(eGFR, 60) – 60) / -15 |
|  | Risk= 1 / (1 + exp(-log-Odds)) |
| Women | log-Odds = -3.307728 + 0.7939329 × (age – 55) /10 + 0.0305239 × ((TC – HDL-C) × 0.02586 – 3.5) – 0.1606857 × (HDL-C × 0.02586 – 1.3) /0.3 – 0.2394003 × (min(SBP, 110) – 110) /20 + 0.360078 × (max(SBP, 110) – 130) /20 + 0.8667604 × (if diabetes) + 0.5360739 × (if current smoker) + 0.6045917 × (min(eGFR, 60) – 60) / -15 + 0.0433769 × (max(eGFR, 60) – 90) / -15 + 0.3151672 × (if using anti-hypertensive medication) – 0.1477655 × (if using statin) – 0.0663612 × (if using anti-hypertensive medication) × (max(SBP, 110) – 130) /20 + 0.1197879 × (if using statin) × ((TC – HDL-C) × 0.02586 – 3.5) – 0.0819715 × (age – 55) /10 × ((TC – HDL-C) × 0.02586 – 3.5) + 0.0306769 × (age – 55) /10 × (HDL-C × 0.02586 × 0.02586 – 1.3) /0.3 – 0.0946348 × (age – 55) /10 × (max(SBP, 110) – 130) /20 – 0.27057 × (age – 55) /10 × (if diabetes) – 0.078715 × (age – 55) /10 × (if current smoker) – 0.1637806 × (age – 55) /10 × (min(eGFR, 60) – 60) / -15 |
|  | Risk= 1 / (1 + exp(-log-Odds)) |

Abbreviations: TC, total cholesterol; HDL-C, high-density lipoprotein cholesterol; SBP, systolic blood pressure; eGFR, estimated glomerular filtration rate.

**Table S4 Detailed definitions of CKM staging in NHANES.**

| CKM stages | Definition | Criterion | Threshold for CKM conditions |
| --- | --- | --- | --- |
| Stage 0: No CKM health risk factors | Individuals with normal BMI and waist circumference, normoglycemia, normotension, a normal lipid profile, and no evidence of CKD or subclinical or clinical CVD | All criteria are met | Normal BMI |
|  |  |  | Normal waist circumference |
|  |  |  | Normoglycemia |
|  |  |  | Normotension |
|  |  |  | Normal lipid status |
|  |  |  | Low-risk CKD in KDIGO classification |
|  |  |  | Predicted 10-year CVD risk < 20% |
|  |  |  | No clinical CVD |
| Stage 1: Excess or dysfunctional adiposity | Individuals with overweight/obesity, abdominal obesity, or dysfunctional adipose tissue, without the presence of other metabolic risk factors or CKD | Any of the three criteria is met | Overweight/obesity |
|  |  |  | Abdominal obesity |
|  |  |  | Prediabetes |
|  |  | All criteria are met | Normotension |
|  |  |  | Normal lipid status |
|  |  |  | Low-risk CKD in KDIGO classification |
|  |  |  | Predicted 10-year CVD risk < 20% |
|  |  |  | No clinical CVD |
| Stage 2: Metabolic risk factors and CKD | Individuals with metabolic risk factors (hypertriglyceridemia, hypertension, MetS, diabetes), or CKD | Any of the five criteria is met | Hypertriglyceridemia |
|  |  |  | Hypertension |
|  |  |  | Diabetes |
|  |  |  | MetS |
|  |  |  | Moderate-to-high-risk CKD in KDIGO classification |
|  |  | All criteria are met | No very high-risk CKD in KDIGO classification |
|  |  |  | Predicted 10-year CVD risk < 20% |
|  |  |  | No clinical CVD |
| Stage 3: Subclinical CVD in CKM | Subclinical CVD among individuals with excess/dysfunctional adiposity, other metabolic risk factors, or CKD | Any of the two criteria is met | Very high-risk CKD in KDIGO classification |
|  |  |  | Predicted 10-year CVD risk ≥ 20% |
|  |  | Any of the eight criteria is met | Overweight/obesity |
|  |  |  | Abdominal obesity |
|  |  |  | Prediabetes |
|  |  |  | Hypertriglyceridemia |
|  |  |  | Hypertension |
|  |  |  | Diabetes |
|  |  |  | MetS |
|  |  |  | Moderate-to-high-risk CKD in KDIGO classification |
|  |  | The criterion is met | No clinical CVD |
| Stage 4: Clinical CVD in CKM | Clinical CVD among individuals with excess/dysfunctional adiposity, other metabolic risk factors, or CKD | The criterion is met | Clinical CVD |
|  |  | Any of the nine criteria is met | Overweight/obesity |
|  |  |  | Abdominal obesity |
|  |  |  | Prediabetes |
|  |  |  | Hypertriglyceridemia |
|  |  |  | Hypertension |
|  |  |  | Diabetes |
|  |  |  | MetS |
|  |  |  | Moderate-to-high-risk CKD in KDIGO classification |
|  |  |  | Very high-risk CKD in KDIGO classification |

Abbreviations: CKM, cardiovascular-kidney-metabolic; CVD, cardiovascular disease; CKD, chronic kidney disease; KDIGO, The Kidney Disease: Improving Global Outcomes; UACR, urinary albumin to creatinine ratio; eGFR, estimated glomerular filtration rate; BMI, body mass index; SBP, systolic blood pressure; DBP, diastolic blood pressure; MetS, metabolic syndrome.

**Fig. S1. Weighted correlation matrix and variance inflation factor of covariates.**

**
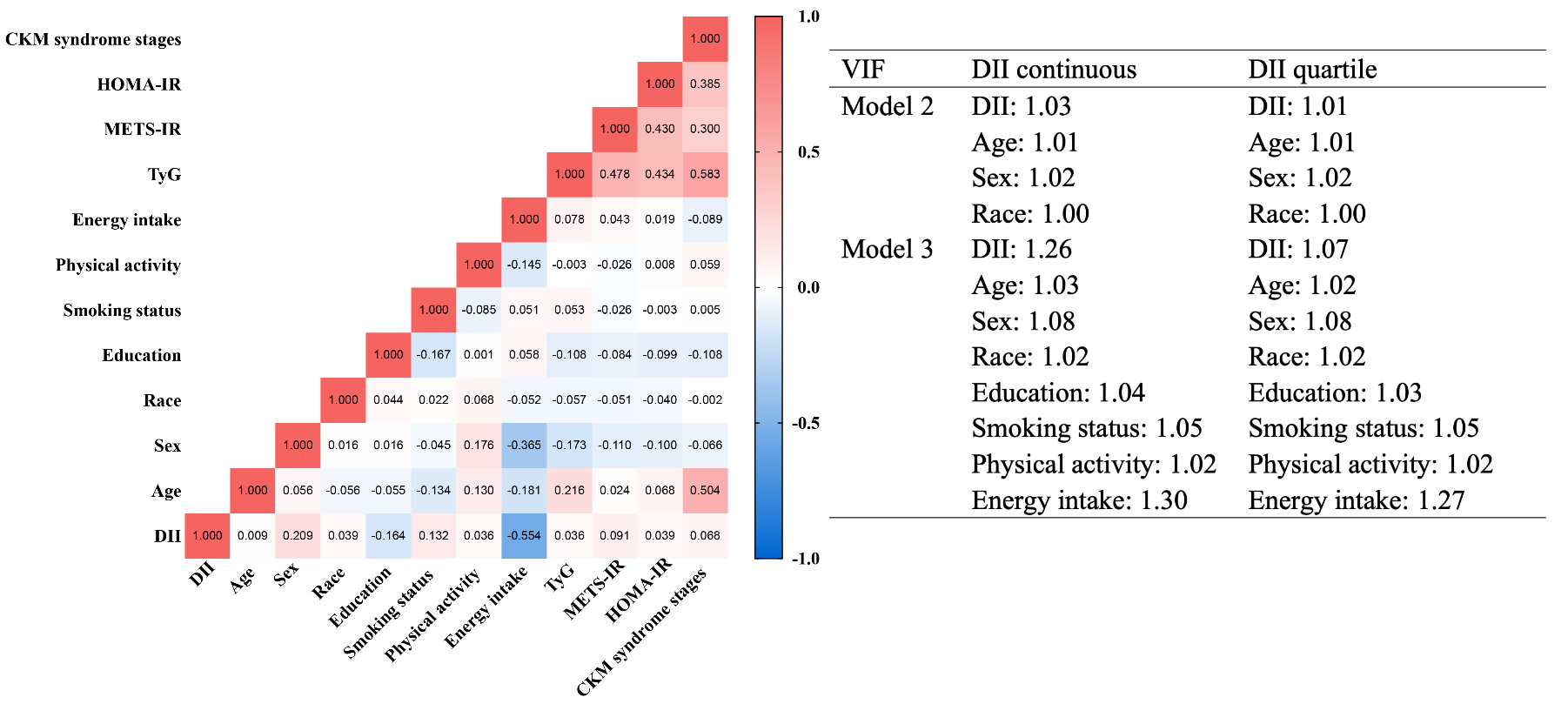
**

Weighted Pearson’s coefficients were calculated for normally distributed variables and weighted Spearman’s coefficients were calculated for non-normally distributed or categorical variables.

Abbreviations: DII, dietary inflammatory index; CKM, Cardiovascular-Kidney-Metabolic; VIF, variance inflation factor.

**Table S5** **Weighted baseline characteristics of participants according to** **Cardiovascular-Kidney-Metabolic syndrome stages (unimputed dataset).**

| Characteristic | N | Total  N = 27,635 | Cardiovascular–kidney–metabolic syndrome stage | | | | | *P* value |
| --- | --- | --- | --- | --- | --- | --- | --- | --- |
|  |  |  | 0  N = 1,635 | 1  N = 6,485 | 2  N = 1,4707 | 3  N = 1,423 | 4  N = 3,385 |  |
| Age, years | 27,635 | 50.57±17.47 | 33.96±12.70 | 40.19±14.19 | 51.28±15.47 | 72.83±9.20 | 66.06±12.73 | <0.001 |
| Male, % | 27,635 | 13,728 (49.68%) | 684 (41.83%) | 2,889 (44.55%) | 7,495 (50.96%) | 722 (50.74%) | 1,938 (57.25%) | <0.001 |
| Race, % | 27,635 |  |  |  |  |  |  | <0.001 |
| Mexican American |  | 4,226 (15.29%) | 142 (8.69%) | 1,241 (19.14%) | 2,365 (16.08%) | 151 (10.61%) | 327 (9.66%) |  |
| Non-Hispanic White |  | 11,572 (41.87%) | 732 (44.77%) | 2,477 (38.20%) | 5,861 (39.85%) | 712 (50.04%) | 1,790 (52.88%) |  |
| Non-Hispanic Black |  | 5,795 (20.97%) | 266 (16.27%) | 1,219 (18.80%) | 3,204 (21.79%) | 350 (24.60%) | 756 (22.33%) |  |
| Other races |  | 6,042 (21.86%) | 495 (30.28%) | 1,548 (23.87%) | 3,277 (22.28%) | 210 (14.76%) | 512 (15.13%) |  |
| Education, % | 27,612 |  |  |  |  |  |  | <0.001 |
| Less than high school |  | 6,731 (24.38%) | 207 (12.67%) | 1,336 (20.61%) | 3,652 (24.85%) | 444 (31.29%) | 1,092 (32.31%) |  |
| High school |  | 6,347 (22.99%) | 304 (18.60%) | 1,396 (21.53%) | 3,419 (23.26%) | 358 (25.23%) | 870 (25.74%) |  |
| Above high school |  | 14,534 (52.64%) | 1,123 (68.73%) | 3,751 (57.86%) | 7,625 (51.88%) | 617 (43.48%) | 1,418 (41.95%) |  |
| Current smoker, % |  | 5,515 (19.97%) | 339 (20.75%) | 1,298 (20.02%) | 2,957 (20.12%) | 190 (13.35%) | 731 (21.60%) | <0.001 |
| Physical activity, % |  |  |  |  |  |  |  | <0.001 |
| Vigorous |  | 5,533 (20.03%) | 349 (21.36%) | 1,467 (22.63%) | 3,123 (21.24%) | 138 (9.70%) | 456 (13.48%) |  |
| Moderate |  | 5,894 (21.34%) | 376 (23.01%) | 1,458 (22.49%) | 3,137 (21.34%) | 271 (19.04%) | 652 (19.28%) |  |
| Inactive |  | 16,197 (58.63%) | 909 (55.63%) | 3,558 (54.88%) | 8,442 (57.42%) | 1,014 (71.26%) | 2,274 (67.24%) |  |
| BMI, kg/m^2^ | 27,394 | 29.58±6.90 | 21.64±2.02 | 29.33±5.63 | 30.34±7.12 | 29.71±6.47 | 30.57±7.34 | <0.001 |
| WC, cm | 26,772 | 100.42±16.13 | 79.03±6.61 | 97.92±13.15 | 102.38±16.14 | 104.58±14.66 | 106.07±16.15 | <0.001 |
| DII, /day | 27,635 | 0.99±2.03 | 0.60±2.09 | 0.86±2.04 | 0.97±2.02 | 1.25±1.99 | 1.38±2.01 | <0.001 |
| Energy intake, Kcal/day | 27,635 | 2,093.61±992.78 | 2,254.88±1,063.12 | 2,192.21±1,000.29 | 2,126.25±1,009.46 | 1,740.66±743.97 | 1,833.37±876.75 | <0.001 |
| TG, mg/dL | 13,291 | 103.00  (71.00, 152.00) | 62.00  (46.00, 82.00) | 78.00  (59.00, 101.00) | 124.00  (82.00, 174.00) | 114.00  (83.00, 156.00) | 113.00  (79.00, 165.00) | <0.001 |
| TC, mg/dL | 27,438 | 192.83±41.83 | 175.92±34.03 | 190.56±37.44 | 199.15±41.87 | 188.11±46.89 | 179.05±44.75 | <0.001 |
| LDL, mg/dL | 13,069 | 113.77±35.62 | 98.84±27.85 | 113.57±31.67 | 118.86± 5.97 | 104.95±35.28 | 100.02±37.45 | <0.001 |
| HDL, mg/dL | 27,438 | 52.43±16.03 | 60.78±15.63 | 53.47±14.92 | 51.69±16.32 | 51.79±15.52 | 49.79±15.81 | <0.001 |
| FBG, mg/dL | 13,440 | 102.00  (94.00, 113.00) | 91.00  (87.00, 95.00) | 98.00  (92.00, 103.00) | 103.00  (96.00, 115.00) | 114.00  (100.00, 140.00) | 109.00  (99.00, 130.00) | <0.001 |
| TyG | 13,274 | 8.64±0.68 | 7.92±0.40 | 8.20±0.40 | 8.80±0.67 | 8.86±0.67 | 8.84±0.69 | <0.001 |
| METS-IR | 13,161 | 44.04±12.58 | 28.86±3.65 | 39.95±8.64 | 46.17±12.83 | 44.84±11.80 | 47.16±13.60 | <0.001 |
| HOMA-IR | 13,276 | 2.66 (1.59, 4.63) | 1.18 (0.79, 1.65) | 2.03 (1.38, 3.04) | 3.06 (1.82, 5.22) | 3.07 (1.77, 5.28) | 3.34 (1.88, 6.02) | <0.001 |
| Scr, mg/dL | 27,135 | 0.85 (0.72, 1.01) | 0.79 (0.68, 0.94) | 0.81 (0.68, 0.94) | 0.84 (0.72, 0.99) | 1.08 (0.86, 1.39) | 0.99 (0.82, 1.22) | <0.001 |
| eGFR, ml/min/1.73m^2^ | 27,135 | 93.58±22.67 | 108.40±16.40 | 103.80±17.00 | 94.63±19.37 | 62.50±23.75 | 74.25±24.09 | <0.001 |
| UACR, mg/g | 27,423 | 7.29  (4.67, 14.34) | 6.16  (4.35, 9.18) | 5.46  (3.88, 8.17) | 7.67  (4.86, 15.45) | 18.24  (8.30, 66.07) | 12.65  (6.50, 36.25) | <0.001 |
| Hypertension, % | 27,635 | 16,204 (58.64%) | 0 (0.00%) | 0 (0.00%) | 11,933 (81.14%) | 1,401 (98.45%) | 2,870 (84.79%) | <0.001 |
| Diabetes, % | 27,635 | 5,228 (18.92%) | 0 (0.00%) | 0 (0.00%) | 3,107 (21.13%) | 724 (50.88%) | 1,397 (41.27%) | <0.001 |
| CKD, % | 27,291 |  |  |  |  |  |  | <0.001 |
| Low-risk |  | 22,627 (82.91%) | 1,635 (100.00%) | 6,485 (100.00%) | 12,063 (82.16%) | 576 (41.29%) | 1,868 (60.39%) |  |
| Moderate-to-high-risk |  | 4,122 (15.10%) | 0 (0.00%) | 0 (0.00%) | 2,620 (17.84%) | 544 (39.00%) | 958 (30.97%) |  |
| Very high-risk |  | 542 (1.99%) | 0 (0.00%) | 0 (0.00%) | 0 (0.00%) | 275 (19.71%) | 267 (8.63%) |  |
| CVD history | 27,635 | 3,385 (12.25%) | 0 (0.00%) | 0 (0.00%) | 0 (0.00%) | 0 (0.00%) | 3,385 (100.00%) | <0.001 |

Data are presented as weighted mean ± standard deviation for normally distributed continuous variables, weighted median (interquartile range) for non-normally distributed continuous variables, or number (weighted percentage) for categorical variables.

Abbreviations: BMI: body mass index; WC, waist circumference; DII, dietary inflammatory index; TG: triglyceride; TC, total cholesterol; LDL, low-density lipoprotein cholesterol; HDL, high-density lipoprotein cholesterol; FBG, fasting blood glucose; TyG, triglyceride–glucose index; METS-IR, Metabolic Score for IR; HOMA-IR, Homeostatic Model Assessment for IR; Scr, serum creatinine; eGFR, estimated glomerular filtration rate; UACR, urinary albumin creatinine ratio; CKD, chronic kidney disease; CVD, cardiovascular disease.

**Table S6** **Weighted baseline characteristics of participants according to advanced Cardiovascular-Kidney-Metabolic syndrome stages.**

| Characteristic | Cardiovascular–kidney–metabolic syndrome stage | | *P* value |
| --- | --- | --- | --- |
|  | Non-advanced  N = 22,827 | Advanced  N = 4,808 |  |
| Age, years | 45.46±15.57 | 67.07±12.78 | <0.001 |
| Male, % | 11,068 (48.69%) | 2,660 (52.92%) | <0.001 |
| Race, % |  |  | <0.001 |
| Mexican American | 3,748 (9.39%) | 478 (4.98%) |  |
| Non-Hispanic White | 9,070 (65.56%) | 2,502 (72.82%) |  |
| Non-Hispanic Black | 4,689 (10.73%) | 1,106 (12.29%) |  |
| Other races | 5,320 (14.32%) | 722 (9.92%) |  |
| Education, % |  |  | <0.001 |
| Less than high school | 5,197 (14.37%) | 1,540 (22.61%) |  |
| High school | 5,121 (22.77%) | 1,230 (27.41%) |  |
| Above high school | 12,509 (62.86%) | 2,038 (49.97%) |  |
| Current smoker, % | 4,595 (19.55%) | 921 (19.37%) | 0.859 |
| Physical activity, % |  |  | <0.001 |
| Vigorous | 4,939 (23.41%) | 595 (14.33%) |  |
| Moderate | 4,972 (24.58%) | 923 (22.13%) |  |
| Inactive | 12,916 (52.01%) | 3,290 (63.54%) |  |
| BMI, kg/m^2^ | 29.23±6.70 | 30.58±7.05 | <0.001 |
| WC, cm | 99.35±15.96 | 106.70±15.65 | <0.001 |
| DII, /day | 0.77±2.04 | 1.15±2.03 | <0.001 |
| Energy intake, Kcal/day | 2,184.15±981.53 | 1,870.70±847.62 | <0.001 |
| TG, mg/dL | 111.93 (89.07, 134.53) | 152.00 (119.00, 179.29) | <0.001 |
| TC, mg/dL | 196.16±40.67 | 183.43±45.30 | <0.001 |
| LDL, mg/dL | 116.98±28.77 | 106.26±31.77 | <0.001 |
| HDL, mg/dL | 53.39±16.46 | 50.23±15.62 | <0.001 |
| FBG, mg/dL | 99.00 (94.63, 104.62) | 107.00 (100.56, 155.60) | <0.001 |
| TyG | 8.63±0.50 | 9.04±0.56 | <0.001 |
| METS-IR | 43.48±12.16 | 47.78±13.37 | <0.001 |
| HOMA-IR | 2.93 (2.02, 3.91) | 3.99 (2.75, 8.01) | <0.001 |
| Scr, mg/dL | 0.84 (0.72, 0.98) | 0.98 (0.81, 1.22) | <0.001 |
| eGFR, ml/min/1.73m^2^ | 98.57±18.40 | 73.84±23.81 | <0.001 |
| UACR, mg/g | 6.27 (4.27, 10.70) | 12.69 (6.75, 40.63) | <0.001 |
| Hypertension, % | 11,933 (49.39%) | 4,271 (86.34%) | <0.001 |
| Diabetes, % | 3,107 (9.95%) | 2,121 (40.41%) | <0.001 |
| CKD, % |  |  | <0.001 |
| Low-risk | 20,188 (90.61%) | 2,551 (58.19%) |  |
| Moderate-to-high-risk | 2,639 (9.39%) | 1,686 (32.77%) |  |
| Very high-risk | 0 (0.00%) | 571 (9.04%) |  |
| CVD history | 0 (0.00%) | 3,385 (71.62%) | <0.001 |

Data are presented as weighted mean ± standard deviation for normally distributed continuous variables, weighted median (interquartile range) for non-normally distributed continuous variables, or number (weighted percentage) for categorical variables.

Advanced CKM stages was defined as Stage 3—4 (high-risk or established cardiovascular disease).

Abbreviations: BMI: body mass index; WC, waist circumference; DII, dietary inflammatory index; TG: triglyceride; TC, total cholesterol; LDL, low-density lipoprotein cholesterol; HDL, high-density lipoprotein cholesterol; FBG, fasting blood glucose; TyG, triglyceride–glucose index; METS-IR, Metabolic Score for IR; HOMA-IR, Homeostatic Model Assessment for IR; Scr, serum creatinine; eGFR, estimated glomerular filtration rate; UACR, urinary albumin creatinine ratio; CKD, chronic kidney disease; CVD, cardiovascular disease.

**Table S7 Weighted baseline characteristics of participants according to the quartile level of dietary inflammatory index.**

| Characteristic | DII quartile | | | | *P* value |
| --- | --- | --- | --- | --- | --- |
|  | 1 (<-0.69) | 2 (-0.69–0.99) | 3 (0.99-2.42) | 4 (≥2.42) |  |
| Age, years | 48.23±16.23 | 48.36±16.60 | 48.39±17.25 | 48.53±17.56 | 0.983 |
| Male, % | 3,839 (61.83%) | 3,737 (55.49%) | 3,263 (43.85%) | 2,889 (35.88%) | <0.001 |
| Race, % |  |  |  |  | <0.001 |
| Mexican American | 1,004 (9.01%) | 1,147 (9.68%) | 1,042 (8.54%) | 1,033 (7.97%) |  |
| Non-Hispanic White | 2,644 (69.26%) | 2,861 (67.16%) | 2,955 (66.43%) | 3,112 (63.29%) |  |
| Non-Hispanic Black | 1,026 (7.69%) | 1,225 (9.31%) | 1,529 (11.66%) | 2,015 (15.09%) |  |
| Other races | 1,482 (14.04%) | 1,527 (13.85%) | 1,499 (13.37%) | 1,534 (13.66%) |  |
| Education, % |  |  |  |  | <0.001 |
| Less than high school | 1,149 (10.85%) | 1,489 (14.08%) | 1,748 (15.93%) | 2,351 (21.05%) |  |
| High school | 1,149 (17.62%) | 1,497 (22.13%) | 1,707 (25.39%) | 1,998 (28.45%) |  |
| Above high school | 3,858 (71.53%) | 3,774 (63.79%) | 3,570 (58.68%) | 3,345 (50.50%) |  |
| Current smoker, % | 944 (13.52%) | 1,151 (16.90%) | 1,461 (20.31%) | 1,960 (27.38%) | <0.001 |
| Physical activity, % |  |  |  |  | 0.005 |
| Vigorous | 1,356 (22.84%) | 1,412 (23.25%) | 1,406 (22.61%) | 1,360 (20.03%) |  |
| Moderate | 1,394 (25.94%) | 1,461 (23.54%) | 1,496 (23.88%) | 1,544 (23.64%) |  |
| Inactive | 3,406 (51.21%) | 3,887 (53.21%) | 4,123 (53.51%) | 4,790 (56.33%) |  |
| BMI, kg/m2 | 28.53±6.32 | 29.21±6.54 | 29.79±6.93 | 30.12±7.13 | <0.001 |
| WC, cm | 98.96±15.80 | 100.20±15.89 | 100.88±16.33 | 101.33±16.34 | <0.001 |
| DII, /day | -1.93±0.94 | 0.18±0.48 | 1.72±0.41 | 3.33±0.61 | <0.001 |
| Energy intake, Kcal/day | 2,843.46±1,122.40 | 2,347.47±810.21 | 1,937.23±653.52 | 1,439.29±595.18 | <0.001 |
| TG, mg/dL | 113.98 (88.00, 142.07) | 116.30 (91.00, 142.03) | 114.67 (93.00, 141.19) | 115.52 (92.66, 144.00) | 0.103 |
| TC, mg/dL | 193.34±40.98 | 194.24±40.24 | 195.20±42.14 | 194.99±42.78 | 0.468 |
| LDL, mg/dL | 113.98±28.86 | 115.49±28.59 | 116.26±29.79 | 116.41±30.35 | 0.016 |
| HDL, mg/dL | 53.70±16.47 | 52.55±16.24 | 53.42±16.54 | 52.21±16.27 | 0.001 |
| FBG, mg/dL | 99.73 (95.00, 106.00) | 100.00 (95.53, 106.00) | 100.00 (95.04, 106.00) | 99.81 (95.00, 107.00) | 0.294 |
| TyG | 8.66±0.53 | 8.69±0.53 | 8.69±0.51 | 8.70±0.53 | 0.036 |
| METS-IR | 42.56±11.96 | 43.89±12.23 | 44.54±12.66 | 45.27±12.64 | 0.086 |
| HOMA-IR | 2.86 (1.95, 3.93) | 3.02 (2.05, 4.09) | 3.03 (2.16, 4.21) | 3.15 (2.22, 4.38) | <0.001 |
| Scr, mg/dL | 0.88 (0.74, 1.01) | 0.87 (0.73, 1.01) | 0.84 (0.71, 0.99) | 0.82 (0.71, 0.99) | <0.001 |
| eGFR, ml/min/1.73m2 | 96.32±19.07 | 95.49±20.27 | 95.16±21.3 | 93.98±22.98 | 0.045 |
| UACR, mg/g | 6.11 (4.12, 10.76) | 6.45 (4.30, 11.61) | 6.99 (4.50, 12.69) | 7.44 (4.79, 14.27) | <0.001 |
| CKM syndrome, % |  |  |  |  | <0.001 |
| Stage 0 | 458 (9.67%) | 424 (8.05%) | 386 (7.37%) | 367 (6.23%) |  |
| Stage 1 | 1,574 (27.79%) | 1,621 (25.11%) | 1,643 (26.28%) | 1,647 (25.42%) |  |
| Stage 2 | 3,281 (51.55%) | 3,682 (54.45%) | 3,709 (52.22%) | 4,035 (51.93%) |  |
| Stage 3 | 261 (3.29%) | 327 (3.95%) | 385 (3.85%) | 450 (4.22%) |  |
| Stage 4 | 582 (7.69%) | 706 (8.44%) | 902 (10.28%) | 1,195 (12.20%) |  |
| Advanced | 5,313 (89.02%) | 5,727 (87.61%) | 5,738 (85.86%) | 6,049 (83.58%) | <0.001 |
| Non-advanced | 843 (10.98%) | 1,033 (12.39%) | 1,287 (14.14%) | 1,645 (16.42%) |  |
| Hypertension, % | 3,408 (51.62%) | 3,910 (54.97%) | 4,161 (55.26%) | 4,725 (55.63%) | 0.006 |
| Diabetes, % | 988 (11.74%) | 1,191 (13.61%) | 1,384 (14.15%) | 1,665 (16.73%) | <0.001 |
| CKD, % |  |  |  |  | <0.001 |
| Low-risk | 5,302 (89.13%) | 5,676 (87.35%) | 5,742 (85.58%) | 6,019 (82.90%) |  |
| Moderate-to-high-risk | 794 (10.29%) | 982 (11.79%) | 1,125 (13.10%) | 1,424 (15.00%) |  |
| Very high-risk | 60 (0.59%) | 102 (0.87%) | 158 (1.32%) | 251 (2.10%) |  |
| CVD history | 582 (7.69%) | 706 (8.44%) | 902 (10.28%) | 1,195 (12.20%) | <0.001 |

Data are presented as weighted mean ± standard deviation for normally distributed continuous variables, weighted median (interquartile range) for non-normally distributed continuous variables, or number (weighted percentage) for categorical variables.

Advanced CKM stages was defined as Stage 3—4 (high-risk or established cardiovascular disease).

Abbreviations: BMI: body mass index; WC, waist circumference; DII, dietary inflammatory index; TG: triglyceride; TC, total cholesterol; LDL, low-density lipoprotein cholesterol; HDL, high-density lipoprotein cholesterol; FBG, fasting blood glucose; TyG, triglyceride–glucose index; METS-IR, Metabolic Score for IR; HOMA-IR, Homeostatic Model Assessment for IR; Scr, serum creatinine; eGFR, estimated glomerular filtration rate; UACR, urinary albumin creatinine ratio; CKD, chronic kidney disease; CVD, cardiovascular disease; CKM, Cardiovascular-Kidney-Metabolic.

**Table S8** **Mediation effects of TyG between dietary inflammatory index and Cardiovascular-Kidney-Metabolic syndrome stages.**

| **Model 1** | | | | | |
| --- | --- | --- | --- | --- | --- |
| **Exposure to Mediator (a: DII → TyG)** | | | **Indirect Effect (a*b: DII → TyG → CKM stages)** | | |
| $\beta$ (SE) | *P* value | Standardized $\beta$ | $\beta$ (95% CI) | *P* value | Standardized $\beta$ |
| 0.008 (0.002) | <0.001 | 0.028 | 0.009 (0.005, 0.012) | <0.001 | 0.015 |
| **Mediator to Outcome (b: TyG → CKM stages)** | | | **Total Effect (c+a*b: DII → CKM stages)** | | |
| $\beta$ (SE) | *P* value | Standardized $\beta$ | $\beta$ (95% CI) | *P* value | Standardized $\beta$ |
| 1.136 (0.008) | <0.001 | 0.532 | 0.056 (0.049, 0.064) | <0.001 | 0.096 |
| **Direct Effect (c: DII** $\frac{\boldsymbol{\times}\mathbf{TyG}}{\boldsymbol{\to}}$ **CKM stages)** | | | **Proportion Mediated (a*b/c+a*b)** | | |
| $\beta$ (SE) | *P* value | Standardized $\beta$ | 15.5% |  |  |
| 0.047 (0.003) | <0.001 | 0.081 |  |  |  |
| **Model 2** |  |  |  |  |  |
| **Exposure to Mediator (a: DII → TyG)** | | | **Indirect Effect (a*b: DII → TyG → CKM stages)** | | |
| $\beta$ (SE) | *P* value | Standardized $\beta$ | $\beta$ (95% CI) | *P* value | Standardized $\beta$ |
| 0.014 (0.002) | <0.001 | 0.052 | 0.015 (0.012, 0.018) | <0.001 | 0.021 |
| **Mediator to Outcome (b: TyG → CKM stages)** | | | **Total Effect (c+a*b: DII → CKM stages)** | | |
| $\beta$ (SE) | *P* value | Standardized $\beta$ | $\beta$ (95% CI) | *P* value | Standardized $\beta$ |
| 1.038 (0.009) | <0.001 | 0.406 | 0.070 (0.062, 0.077) | <0.001 | 0.100 |
| **Direct Effect (c: DII** $\frac{\boldsymbol{\times}\mathbf{TyG}}{\boldsymbol{\to}}$ **CKM stages)** | | | **Proportion Mediated (a*b/c+a*b)** | | |
| $\beta$ (SE) | *P* value | Standardized $\beta$ | 21.3% |  |  |
| 0.055 (0.003) | <0.001 | 0.078 |  |  |  |
| **Model 3** |  |  |  |  |  |
| **Exposure to Mediator (a: DII → TyG)** | | | **Indirect Effect (a*b: DII → TyG → CKM stages)** | | |
| $\beta$ (SE) | *P* value | Standardized $\beta$ | $\beta$ (95% CI) | *P* value | Standardized $\beta$ |
| 0.018 (0.002) | <0.001 | 0.065 | 0.018 (0.015, 0.022) | <0.001 | 0.026 |
| **Mediator to Outcome (b: TyG → CKM stages)** | | | **Total Effect (c+a*b: DII → CKM stages)** | | |
| $\beta$ (SE) | *P* value | Standardized $\beta$ | $\beta$ (95% CI) | *P* value | Standardized $\beta$ |
| 1.033 (0.009) | <0.001 | 0.403 | 0.060 (0.051, 0.069) | <0.001 | 0.086 |
| **Direct Effect (c: DII** $\frac{\boldsymbol{\times}\mathbf{TyG}}{\boldsymbol{\to}}$ **CKM stages)** | | | **Proportion Mediated (a*b/c+a*b)** | | |
| $\beta$ (SE) | *P* value | Standardized $\beta$ | 30.7% |  |  |
| 0.042 (0.004) | <0.001 | 0.059 |  |  |  |

Model 1: unadjusted.

Model 2: adjusted for age, gender, and race.

Model 3: adjusted for age, gender, race, education, smoking status, physical activity, and total energy intake.

Abbreviations: DII, dietary inflammatory index; CKM, Cardiovascular-Kidney-Metabolic; TyG, triglyceride–glucose index.

**Table S9** **Mediation effects of METS-IR between dietary inflammatory index and Cardiovascular-Kidney-Metabolic syndrome stages.**

| **Model 1** | | | | | |
| --- | --- | --- | --- | --- | --- |
| **Exposure to Mediator (a: DII →** **METS-IR)** | | | **Indirect Effect (a*b: DII → METS-IR → CKM stages)** | | |
| $\beta$ (SE) | *P* value | Standardized $\beta$ | $\beta$ (95% CI) | *P* value | Standardized $\beta$ |
| 0.506 (0.037) | <0.001 | 0.083 | 0.012 (0.011, 0.014) | <0.001 | 0.024 |
| **Mediator to Outcome (b: METS-IR → CKM stages)** | | | **Total Effect (c+a*b: DII → CKM stages)** | | |
| $\beta$ (SE) | *P* value | Standardized $\beta$ | $\beta$ (95% CI) | *P* value | Standardized $\beta$ |
| 0.025 (0.000) | <0.001 | 0.293 | 0.050 (0.043, 0.056) | <0.001 | 0.096 |
| **Direct Effect (c: DII** $\frac{\boldsymbol{\times}\mathbf{METS-IR}}{\boldsymbol{\to}}$ **CKM stages)** | | | **Proportion Mediated (a*b/c+a*b)** | | |
| $\beta$ (SE) | *P* value | Standardized $\beta$ | 25.2% |  |  |
| 0.037 (0.003) | <0.001 | 0.072 |  |  |  |
| **Model 2** |  |  |  |  |  |
| **Exposure to Mediator (a: DII →METS-IR)** | | | **Indirect Effect (a*b: DII → METS-IR → CKM stages)** | | |
| $\beta$ (SE) | *P* value | Standardized $\beta$ | $\beta$ (95% CI) | *P* value | Standardized $\beta$ |
| 0.583 (0.038) | <0.001 | 0.095 | 0.018 (0.015, 0.020) | <0.001 | 0.027 |
| **Mediator to Outcome (b: METS-IR → CKM stages)** | | | **Total Effect (c+a*b: DII → CKM stages)** | | |
| $\beta$ (SE) | *P* value | Standardized $\beta$ | $\beta$ (95% CI) | *P* value | Standardized $\beta$ |
| 0.030 (0.001) | <0.001 | 0.285 | 0.065 (0.058, 0.072) | <0.001 | 0.100 |
| **Direct Effect (c: DII** $\frac{\boldsymbol{\times}\mathbf{METS-IR}}{\boldsymbol{\to}}$ **CKM stages)** | | | **Proportion Mediated (a*b/c+a*b)** | | |
| $\beta$ (SE) | *P* value | Standardized $\beta$ | 27.2% |  |  |
| 0.047 (0.003) | <0.001 | 0.073 |  |  |  |
| **Model 3** |  |  |  |  |  |
| **Exposure to Mediator (a: DII →METS-IR)** | | | **Indirect Effect (a*b: DII → METS-IR → CKM stages)** | | |
| $\beta$ (SE) | *P* value | Standardized $\beta$ | $\beta$ (95% CI) | *P* value | Standardized $\beta$ |
| 0.806 (0.043) | <0.001 | 0.131 | 0.025 (0.023, 0.028) | <0.001 | 0.039 |
| **Mediator to Outcome (b: METS-IR → CKM stages)** | | | **Total Effect (c+a*b: DII → CKM stages)** | | |
| $\beta$ (SE) | *P* value | Standardized $\beta$ | $\beta$ (95% CI) | *P* value | Standardized $\beta$ |
| 0.032 (0.001) | <0.001 | 0.294 | 0.057 (0.048, 0.065) | <0.001 | 0.086 |
| **Direct Effect (c: DII** $\frac{\boldsymbol{\times}\mathbf{METS-IR}}{\boldsymbol{\to}}$ **CKM stages)** | | | **Proportion Mediated (a*b/c+a*b)** | | |
| $\beta$ (SE) | *P* value | Standardized $\beta$ | 45.0% |  |  |
| 0.031 (0.004) | <0.001 | 0.047 |  |  |  |

Model 1: unadjusted.

Model 2: adjusted for age, gender, and race.

Model 3: adjusted for age, gender, race, education, smoking status, physical activity, and total energy intake.

Abbreviations: DII, dietary inflammatory index; CKM, Cardiovascular-Kidney-Metabolic; METS-IR, Metabolic Score for IR.

**Table S10** **Mediation effects of HOMA-IR between dietary inflammatory index and Cardiovascular-Kidney-Metabolic syndrome stages.**

| **Model 1** | | | | | |
| --- | --- | --- | --- | --- | --- |
| **Exposure to Mediator (a: DII →** **HOMA-IR)** | | | **Indirect Effect (a*b: DII → HOMA-IR → CKM stages)** | | |
| $\beta$ (SE) | *P* value | Standardized $\beta$ | $\beta$ (95% CI) | *P* value | Standardized $\beta$ |
| 0.095 (0.018) | <0.001 | 0.035 | 0.009 (0.006, 0.012) | <0.001 | 0.016 |
| **Mediator to Outcome (b: HOMA-IR → CKM stages)** | | | **Total Effect (c+a*b: DII → CKM stages)** | | |
| $\beta$ (SE) | *P* value | Standardized $\beta$ | $\beta$ (95% CI) | *P* value | Standardized $\beta$ |
| 0.096 (0.000) | <0.001 | 0.466 | 0.054 (0.047, 0.061) | <0.001 | 0.096 |
| **Direct Effect (c: DII** $\frac{\boldsymbol{\times}\mathbf{HOMA-IR}}{\boldsymbol{\to}}$ **CKM stages)** | | | **Proportion Mediated (a*b/c+a*b)** | | |
| $\beta$ (SE) | *P* value | Standardized $\beta$ | 16.9% |  |  |
| 0.045 (0.004) | <0.001 | 0.080 |  |  |  |
| **Model 2** |  |  |  |  |  |
| **Exposure to Mediator (a: DII →HOMA-IR)** | | | **Indirect Effect (a*b: DII → HOMA-IR → CKM stages)** | | |
| $\beta$ (SE) | *P* value | Standardized $\beta$ | $\beta$ (95% CI) | *P* value | Standardized $\beta$ |
| 0.114 (0.020) | <0.001 | 0.042 | 0.012 (0.008, 0.016) | <0.001 | 0.017 |
| **Mediator to Outcome (b: HOMA-IR → CKM stages)** | | | **Total Effect (c+a*b: DII → CKM stages)** | | |
| $\beta$ (SE) | *P* value | Standardized $\beta$ | $\beta$ (95% CI) | *P* value | Standardized $\beta$ |
| 0.102 (0.000) | <0.001 | 0.395 | 0.070 (0.062, 0.077) | <0.001 | 0.100 |
| **Direct Effect (c: DII** $\frac{\boldsymbol{\times}\mathbf{HOMA-IR}}{\boldsymbol{\to}}$ **CKM stages)** | | | **Proportion Mediated (a*b/c+a*b)** | | |
| $\beta$ (SE) | *P* value | Standardized $\beta$ | 16.6% |  |  |
| 0.058 (0.004) | <0.001 | 0.083 |  |  |  |
| **Model 3** |  |  |  |  |  |
| **Exposure to Mediator (a: DII → HOMA-IR)** | | | **Indirect Effect (a*b: DII → HOMA-IR → CKM stages)** | | |
| $\beta$ (SE) | *P* value | Standardized $\beta$ | $\beta$ (95% CI) | *P* value | Standardized $\beta$ |
| 0.143 (0.025) | <0.001 | 0.052 | 0.016 (0.01, 0.021) | <0.001 | 0.022 |
| **Mediator to Outcome (b: HOMA-IR → CKM stages)** | | | **Total Effect (c+a*b: DII → CKM stages)** | | |
| $\beta$ (SE) | *P* value | Standardized $\beta$ | $\beta$ (95% CI) | *P* value | Standardized $\beta$ |
| 0.109 (0.000) | <0.001 | 0.413 | 0.061 (0.053, 0.070) | <0.001 | 0.086 |
| **Direct Effect (c: DII** $\frac{\boldsymbol{\times}\mathbf{HOMA-IR}}{\boldsymbol{\to}}$ **CKM stages)** | | | **Proportion Mediated (a*b/c+a*b)** | | |
| $\beta$ (SE) | *P* value | Standardized $\beta$ | 25.3% |  |  |
| 0.046 (0.005) | <0.001 | 0.064 |  |  |  |

Model 1: unadjusted.

Model 2: adjusted for age, gender, and race.

Model 3: adjusted for age, gender, race, education, smoking status, physical activity, and total energy intake.

Abbreviations: DII, dietary inflammatory index; CKM, Cardiovascular-Kidney-Metabolic; HOMA-IR, Homeostatic Model Assessment for IR.

**Table S11 Mediation effects of TyG between dietary inflammatory index and advanced Cardiovascular-Kidney-Metabolic syndrome stages.**

| **Model 1** | | | | | |
| --- | --- | --- | --- | --- | --- |
| **Exposure to Mediator (a: DII → TyG)** | | | **Indirect Effect (a*b: DII → TyG → Advanced CKM stages)** | | |
| $\beta$ (SE) | *P* value | Standardized $\beta$ | $\beta$ (95% CI) | *P* value | Standardized $\beta$ |
| 0.008 (0.002) | <0.001 | 0.028 | 0.006 (0.004, 0.009) | <0.001 | 0.011 |
| **Mediator to Outcome (b: TyG → Advanced CKM stages)** | | | **Total Effect (c+a*b: DII → Advanced CKM stages)** | | |
| $\beta$ (SE) | *P* value | Standardized $\beta$ | $\beta$ (95% CI) | *P* value | Standardized $\beta$ |
| 0.799 (0.013) | <0.001 | 0.403 | 0.065 (0.056, 0.075) | <0.001 | 0.121 |
| **Direct Effect (c: DII** $\frac{\boldsymbol{\times}\mathbf{TyG}}{\boldsymbol{\to}}$ **Advanced CKM stages)** | | | **Proportion Mediated (a*b/c+a*b)** | | |
| $\beta$ (SE) | *P* value | Standardized $\beta$ | 9.3% |  |  |
| 0.059 (0.005) | <0.001 | 0.109 |  |  |  |
| **Model 2** |  |  |  |  |  |
| **Exposure to Mediator (a: DII → TyG)** | | | **Indirect Effect (a*b: DII → TyG → Advanced CKM stages)** | | |
| $\beta$ (SE) | *P* value | Standardized $\beta$ | $\beta$ (95% CI) | *P* value | Standardized $\beta$ |
| 0.014 (0.002) | <0.001 | 0.052 | 0.010 (0.007, 0.012) | <0.001 | 0.014 |
| **Mediator to Outcome (b: TyG → Advanced CKM stages)** | | | **Total Effect (c+a*b: DII → Advanced CKM stages)** | | |
| $\beta$ (SE) | *P* value | Standardized $\beta$ | $\beta$ (95% CI) | *P* value | Standardized $\beta$ |
| 0.671 (0.016) | <0.001 | 0.258 | 0.077 (0.066, 0.087) | <0.001 | 0.108 |
| **Direct Effect (c: DII** $\frac{\boldsymbol{\times}\mathbf{TyG}}{\boldsymbol{\to}}$ **Advanced CKM stages)** | | | **Proportion Mediated (a*b/c+a*b)** | | |
| $\beta$ (SE) | *P* value | Standardized $\beta$ | 12.6% |  |  |
| 0.067 (0.005) | <0.001 | 0.094 |  |  |  |
| **Model 3** |  |  |  |  |  |
| **Exposure to Mediator (a: DII → TyG)** | | | **Indirect Effect (a*b: DII → TyG → Advanced CKM stages)** | | |
| $\beta$ (SE) | *P* value | Standardized $\beta$ | $\beta$ (95% CI) | *P* value | Standardized $\beta$ |
| 0.018 (0.002) | <0.001 | 0.065 | 0.012 (0.009, 0.014) | <0.001 | 0.016 |
| **Mediator to Outcome (b: TyG → Advanced CKM stages)** | | | **Total Effect (c+a*b: DII → Advanced CKM stages)** | | |
| $\beta$ (SE) | *P* value | Standardized $\beta$ | $\beta$ (95% CI) | *P* value | Standardized $\beta$ |
| 0.666 (0.016) | <0.001 | 0.252 | 0.050 (0.038, 0.063) | <0.001 | 0.070 |
| **Direct Effect (c: DII** $\frac{\boldsymbol{\times}\mathbf{TyG}}{\boldsymbol{\to}}$ **Advanced CKM stages)** | | | **Proportion Mediated (a*b/c+a*b)** | | |
| $\beta$ (SE) | *P* value | Standardized $\beta$ | 23.5% |  |  |
| 0.039 (0.006) | <0.001 | 0.053 |  |  |  |

Advanced CKM stages was defined as Stage 3—4 (high-risk or established cardiovascular disease).

Model 1: unadjusted.

Model 2: adjusted for age, gender, and race.

Model 3: adjusted for age, gender, race, education, smoking status, physical activity, and total energy intake.

Abbreviations: DII, dietary inflammatory index; CKM, Cardiovascular-Kidney-Metabolic; TyG, triglyceride–glucose index.

**Table S12 Mediation effects of METS-IR between dietary inflammatory index and advanced Cardiovascular-Kidney-Metabolic syndrome stages.**

| **Model 1** | | | | | |
| --- | --- | --- | --- | --- | --- |
| **Exposure to Mediator (a: DII → METS-IR)** | | | **Indirect Effect (a*b: DII → METS-IR → Advanced CKM stages)** | | |
| $\beta$ (SE) | *P* value | Standardized $\beta$ | $\beta$ (95% CI) | *P* value | Standardized $\beta$ |
| 0.506 (0.037) | <0.001 | 0.083 | 0.006 (0.005, 0.007) | <0.001 | 0.011 |
| **Mediator to Outcome (b: METS-IR → Advanced CKM stages)** | | | **Total Effect (c+a*b: DII → Advanced CKM stages)** | | |
| $\beta$ (SE) | *P* value | Standardized $\beta$ | $\beta$ (95% CI) | *P* value | Standardized $\beta$ |
| 0.011 (0.001) | <0.001 | 0.136 | 0.060 (0.052, 0.069) | <0.001 | 0.121 |
| **Direct Effect (c: DII** $\frac{\boldsymbol{\times}\mathbf{METS-IR}}{\boldsymbol{\to}}$ **Advanced CKM stages)** | | | **Proportion Mediated (a*b/c+a*b)** | | |
| $\beta$ (SE) | *P* value | Standardized $\beta$ | 9.3% |  |  |
| 0.055 (0.004) | <0.001 | 0.110 |  |  |  |
| **Model 2** |  |  |  |  |  |
| **Exposure to Mediator (a: DII → METS-IR)** | | | **Indirect Effect (a*b: DII → METS-IR → Advanced CKM stages)** | | |
| $\beta$ (SE) | *P* value | Standardized $\beta$ | $\beta$ (95% CI) | *P* value | Standardized $\beta$ |
| 0.583 (0.038) | <0.001 | 0.095 | 0.010 (0.009, 0.012) | <0.001 | 0.015 |
| **Mediator to Outcome (b: METS-IR → Advanced CKM stages)** | | | **Total Effect (c+a*b: DII → Advanced CKM stages)** | | |
| $\beta$ (SE) | *P* value | Standardized $\beta$ | $\beta$ (95% CI) | *P* value | Standardized $\beta$ |
| 0.018 (0.001) | <0.001 | 0.157 | 0.074 (0.063, 0.084) | <0.001 | 0.108 |
| **Direct Effect (c: DII** $\frac{\boldsymbol{\times}\mathbf{METS-IR}}{\boldsymbol{\to}}$ **Advanced CKM stages)** | | | **Proportion Mediated (a*b/c+a*b)** | | |
| $\beta$ (SE) | *P* value | Standardized $\beta$ | 13.9% |  |  |
| 0.064 (0.005) | <0.001 | 0.093 |  |  |  |
| **Model 3** |  |  |  |  |  |
| **Exposure to Mediator (a: DII →METS-IR)** | | | **Indirect Effect (a*b: DII → METS-IR → Advanced CKM stages)** | | |
| $\beta$ (SE) | *P* value | Standardized $\beta$ | $\beta$ (95% CI) | *P* value | Standardized $\beta$ |
| 0.806 (0.043) | <0.001 | 0.131 | 0.017 (0.015, 0.019 | <0.001 | 0.024 |
| **Mediator to Outcome (b: METS-IR → Advanced CKM stages)** | | | **Total Effect (c+a*b: DII → Advanced CKM stages)** | | |
| $\beta$ (SE) | *P* value | Standardized $\beta$ | $\beta$ (95% CI) | *P* value | Standardized $\beta$ |
| 0.021 (0.001) | <0.001 | 0.181 | 0.049 (0.037, 0.062) | <0.001 | 0.070 |
| **Direct Effect (c: DII** $\frac{\boldsymbol{\times}\mathbf{METS-IR}}{\boldsymbol{\to}}$ **Advanced CKM stages)** | | | **Proportion Mediated (a*b/c+a*b)** | | |
| $\beta$ (SE) | *P* value | Standardized $\beta$ | 34.1% |  |  |
| 0.032 (0.006) | <0.001 | 0.046 |  |  |  |

Advanced CKM stages was defined as Stage 3—4 (high-risk or established cardiovascular disease).

Model 1: unadjusted.

Model 2: adjusted for age, gender, and race.

Model 3: adjusted for age, gender, race, education, smoking status, physical activity, and total energy intake.

Abbreviations: DII, dietary inflammatory index; CKM, Cardiovascular-Kidney-Metabolic; METS-IR, Metabolic Score for IR.

**Table S13 Mediation effects of HOMA-IR between dietary inflammatory index and advanced Cardiovascular-Kidney-Metabolic syndrome stages.**

| **Model 1^a^** | | | | | |
| --- | --- | --- | --- | --- | --- |
| **Exposure to Mediator (a: DII → HOMA-IR)** | | | **Indirect Effect (a*b: DII → HOMA-IR → Advanced CKM stages)** | | |
| $\beta$ (SE) | *P* value | Standardized $\beta$ | $\beta$ (95% CI) | *P* value | Standardized $\beta$ |
| 0.095 (0.018) | <0.001 | 0.035 | 0.007 (0.004, 0.009) | <0.001 | 0.013 |
| **Mediator to Outcome (b: HOMA-IR → Advanced CKM stages)** | | | **Total Effect (c+a*b: DII → Advanced CKM stages)** | | |
| $\beta$ (SE) | *P* value | Standardized $\beta$ | $\beta$ (95% CI) | *P* value | Standardized $\beta$ |
| 0.072 (0.000) | <0.001 | 0.365 | 0.064 (0.055, 0.074) | <0.001 | 0.121 |
| **Direct Effect (c: DII** $\frac{\boldsymbol{\times}\mathbf{HOMA-IR}}{\boldsymbol{\to}}$ **Advanced CKM stages)** | | | **Proportion Mediated (a*b/c+a*b)** | | |
| $\beta$ (SE) | *P* value | Standardized $\beta$ | 10.5% |  |  |
| 0.058 (0.005) | <0.001 | 0.108 |  |  |  |
| **Model 2^b^** |  |  |  |  |  |
| **Exposure to Mediator (a: DII → HOMA-IR)** | | | **Indirect Effect (a*b: DII → HOMA-IR → Advanced CKM stages)** | | |
| $\beta$ (SE) | *P* value | Standardized $\beta$ | $\beta$ (95% CI) | *P* value | Standardized $\beta$ |
| 0.114 (0.020) | <0.001 | 0.042 | 0.206 (0.134, 0.277) | <0.001 | 0.031 |
| **Mediator to Outcome (b: HOMA-IR →Advanced CKM stages)** | | | **Total Effect (c+a*b: DII → Advanced CKM stages)** | | |
| $\beta$ (SE) | *P* value | Standardized $\beta$ | $\beta$ (95% CI) | *P* value | Standardized $\beta$ |
| 1.808 (0.022) | <0.001 | 0.734 | 0.721 (0.618, 0.824) | <0.001 | 0.108 |
| **Direct Effect (c: DII** $\frac{\boldsymbol{\times}\mathbf{HOMA-IR}}{\boldsymbol{\to}}$ **Advanced CKM stages)** | | | **Proportion Mediated (a*b/c+a*b)** | | |
| $\beta$ (SE) | *P* value | Standardized $\beta$ | 28.5% |  |  |
| 0.515 (0.058) | <0.001 | 0.077 |  |  |  |
| **Model 3^c^** |  |  |  |  |  |
| **Exposure to Mediator (a: DII → HOMA-IR)** | | | **Indirect Effect (a*b: DII → HOMA-IR → Advanced CKM stages)** | | |
| $\beta$ (SE) | *P* value | Standardized $\beta$ | $\beta$ (95% CI) | *P* value | Standardized $\beta$ |
| 0.143 (0.025) | <0.001 | 0.052 | 0.258 (0.170, 0.346) | <0.001 | 0.038 |
| **Mediator to Outcome (b: HOMA-IR → Advanced CKM stages)** | | | **Total Effect (c+a*b: DII → Advanced CKM stages)** | | |
| $\beta$ (SE) | *P* value | Standardized $\beta$ | $\beta$ (95% CI) | *P* value | Standardized $\beta$ |
| 1.809 (0.022) | <0.001 | 0.723 | 0.476 (0.355, 0.596) | <0.001 | 0.070 |
| **Direct Effect (c: DII** $\frac{\boldsymbol{\times}\mathbf{HOMA-IR}}{\boldsymbol{\to}}$ **Advanced CKM stages)** | | | **Proportion Mediated (a*b/c+a*b)** | | |
| $\beta$ (SE) | *P* value | Standardized $\beta$ | 54.2% |  |  |
| 0.218 (0.069) | <0.001 | 0.032 |  |  |  |

Advanced CKM stages was defined as Stage 3—4 (high-risk or established cardiovascular disease).

Model 1: unadjusted.

Model 2: adjusted for age, gender, and race.

Model 3: adjusted for age, gender, race, education, smoking status, physical activity, and total energy intake.

Abbreviations: DII, dietary inflammatory index; CKM, Cardiovascular-Kidney-Metabolic; HOMA-IR, Homeostatic Model Assessment for IR.

**Fig.S2.** **Weighted RCS regression model for dietary inflammatory index with TyG, METS-IR, and HOMA-IR.**


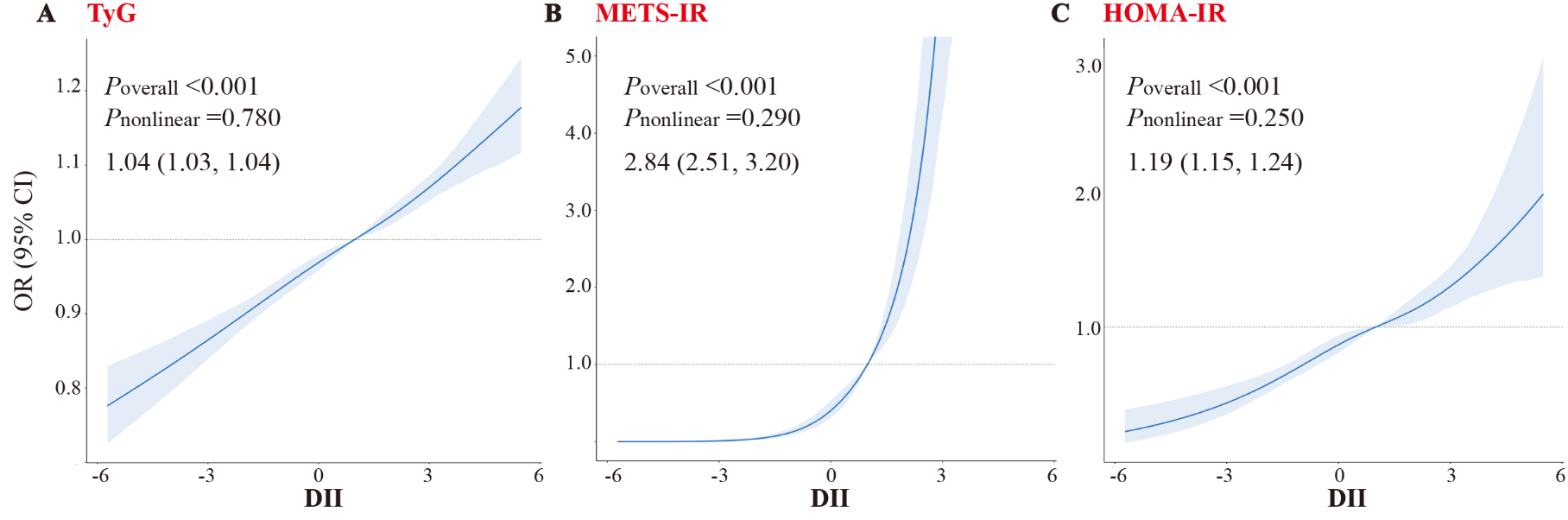


Adjusted for age, gender, race, education, smoking status, physical activity, and total energy intake.

Abbreviations: DII, dietary inflammatory index; TyG, triglyceride–glucose index; METS-IR, Metabolic Score for IR; HOMA-IR, Homeostatic Model Assessment for IR.

**Fig. S3. Weighted RCS regression model for TyG, METS-IR, and HOMA-IR across different** **Cardiovascular-Kidney-Metabolic syndrome stages.**


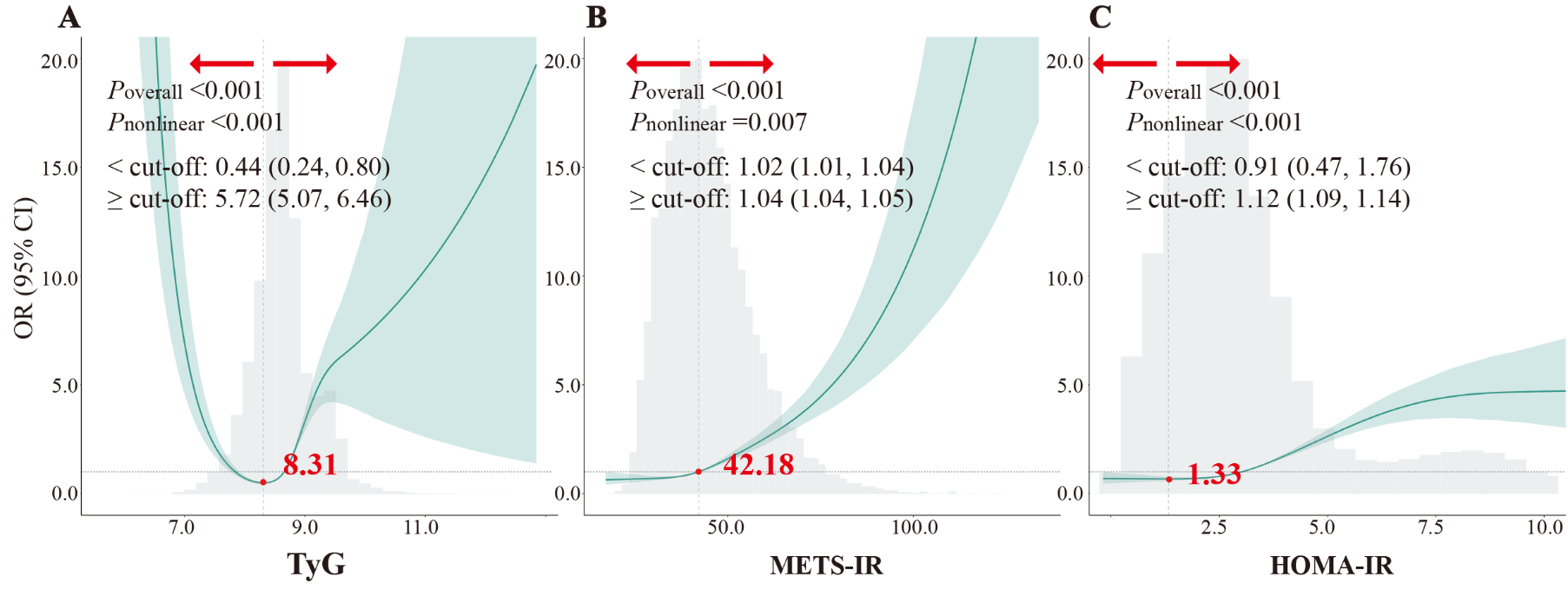


The inflection point is marked with red dot.

Adjusted for age, gender, race, education, smoking status, physical activity, and total energy intake.

Abbreviations: TyG, triglyceride–glucose index; METS-IR, Metabolic Score for IR; HOMA-IR, Homeostatic Model Assessment for IR.

**Table S14** **Weighted interactive effects of dietary inflammatory index and TyG on advanced Cardiovascular-Kidney-Metabolic syndrome stages.**

| Interactive terms | Interactive terms (95% CI) | | |
| --- | --- | --- | --- |
|  | Model 1 | Model 2 | Model 3 |
| Addictive effects |  |  |  |
| RERI | 0.46 (-0.03, 0.84) | 0.18 (-0.41, 0.61) | -0.10 (-0.71, 0.31) |
| AP | 0.13 (-0.01, 0.24) | 0.57 (-0.12, 0.20) | -0.04 (-0.25, 0.13) |
| SI | 1.22 (0.99, 1.55) | 1.09 (0.85, 1.46) | 0.94 (0.71, 1.30) |
| Multiplicative effect | 0.94 (0.69, 1.29) | 0.83 (0.58, 1.18) | 0.83 (0.57, 1.20) |

DII is divided by median (0.99), and TyG is devided by inflection point identified in RCS analysis with CKM syndrome (8.31).

Advanced CKM stages was defined as Stage 3—4 (high-risk or established cardiovascular disease).

Model 1: unadjusted.

Model 2: adjusted for age, gender, and race.

Model 3: adjusted for age, gender, race, education, smoking status, physical activity, and total energy intake.

Abbreviations: RERI, relative excess risk due to interaction; AP, proportion attributable to interaction; SI, synergy index.

**T****able S15 Weighted interactive effects of dietary inflammatory index and METS-IR on advanced Cardiovascular-Kidney-Metabolic syndrome stages.**

| Interactive terms | Interactive terms (95% CI) | | |
| --- | --- | --- | --- |
|  | Model 1 | Model 2 | Model 3 |
| Addictive effects |  |  |  |
| RERI | 0.02 (-0.30, 0.32) | 0.41 (-0.02, 0.81) | 0.12 (-0.28, 0.52) |
| AP | 0.01 (-0.13, 0.13) | 0.14 (-0.01, 0.26) | 0.05 (-0.12, 0.19) |
| SI | 1.02 (0.82, 1.28) | 1.26 (0.99, 1.62) | 1.08 (0.84, 1.45) |
| Multiplicative effect | 0.86 (0.72, 1.02) | 0.98 (0.80, 1.20) | 0.99 (0.81, 1.20) |

DII is divided by median (0.99), and METS-IR is devided by inflection point identified in RCS analysis with CKM syndrome (42.18).

Advanced CKM stages was defined as Stage 3—4 (high-risk or established cardiovascular disease).

Model 1: unadjusted.

Model 2: adjusted for age, gender, and race.

Model 3: adjusted for age, gender, race, education, smoking status, physical activity, and total energy intake.

Abbreviations: RERI, relative excess risk due to interaction; AP, proportion attributable to interaction; SI, synergy index.

**Table S16 Weighted interactive effects of dietary inflammatory index and HOMA-IR on advanced Cardiovascular-Kidney-Metabolic syndrome stages.**

| Interactive terms | Interactive terms (95% CI) | | |
| --- | --- | --- | --- |
|  | Model 1 | Model 2 | Model 3 |
| Addictive effects |  |  |  |
| RERI | -0.11 (-0.79, 0.32) | -0.09 (-0.89, 0.43) | -0.26 (-1.00, 0.24) |
| AP | -0.04 (-0.26, 0.14) | -0.03 (-0.29, 0.16) | -0.11 (-0.38, 0.11) |
| SI | 0.94 (0.71, 1.32) | 0.95 (0.71, 1.39) | 0.85 (0.62, 1.26) |
| Multiplicative effect | 0.76 (0.53, 1.07) | 0.73 (0.51, 1.06) | 0.74 (0.51, 1.07) |

DII is divided by median (0.99), and HOMA-IR is devided by inflection point identified in RCS analysis with CKM syndrome (1.33).

Advanced CKM stages was defined as Stage 3—4 (high-risk or established cardiovascular disease).

Model 1: unadjusted.

Model 2: adjusted for age, gender, and race.

Model 3: adjusted for age, gender, race, education, smoking status, physical activity, and total energy intake.

Abbreviations: RERI, relative excess risk due to interaction; AP, proportion attributable to interaction; SI, synergy index.

**Fig. S4.** **Weighted joint effects of dietary inflammatory index and TyG on advanced Cardiovascular-Kidney-Metabolic syndrome stages.**


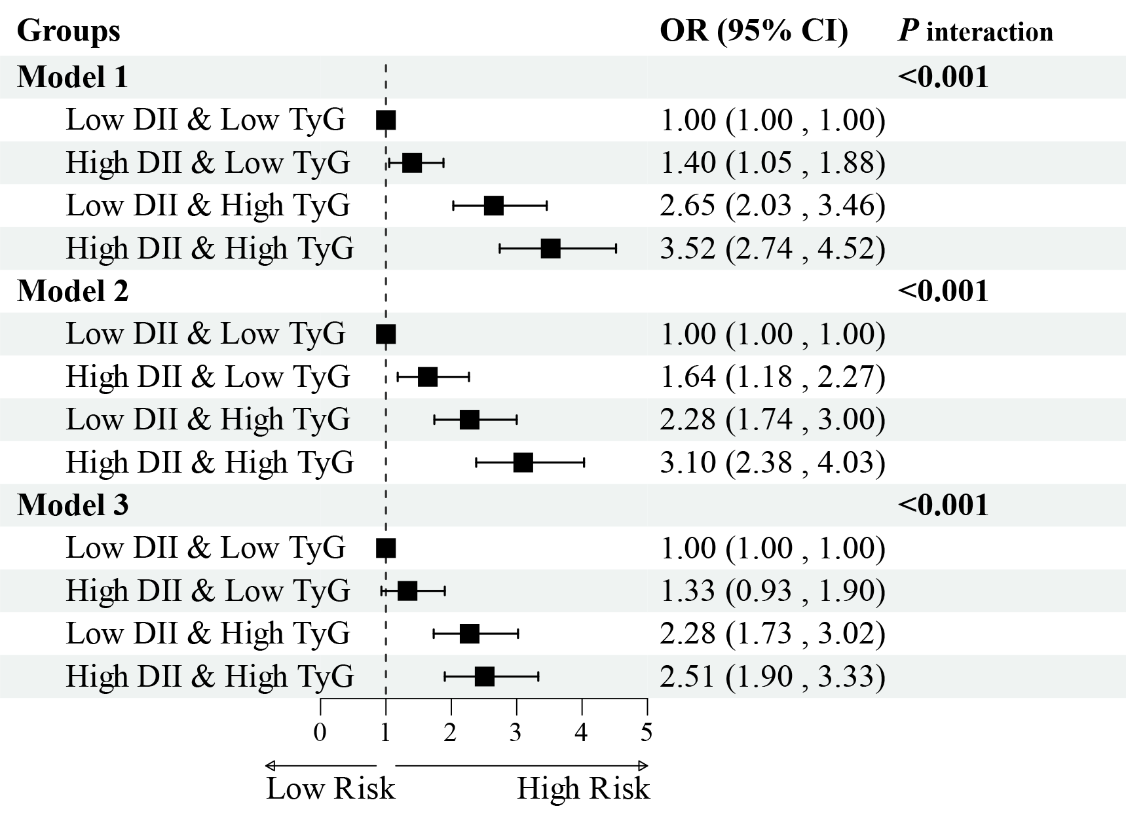


Participants are classified into four groups according to DII and TyG. DII is divided by median (0.99), and TyG is devided by inflection point identified in RCS analysis with CKM syndrome (8.31).

Advanced CKM stages was defined as Stage 3—4 (high-risk or established cardiovascular disease).

Model 1: unadjusted.

Model 2: adjusted for age, gender, and race.

Model 3: adjusted for age, gender, race, education, smoking status, physical activity, and total energy intake.

Abbreviations: DII, dietary inflammatory index; TyG, triglyceride–glucose index.

**Fig. S5.** **Weighted joint effects of dietary inflammatory index and METS-IR on advanced Cardiovascular-Kidney-Metabolic syndrome stages.**


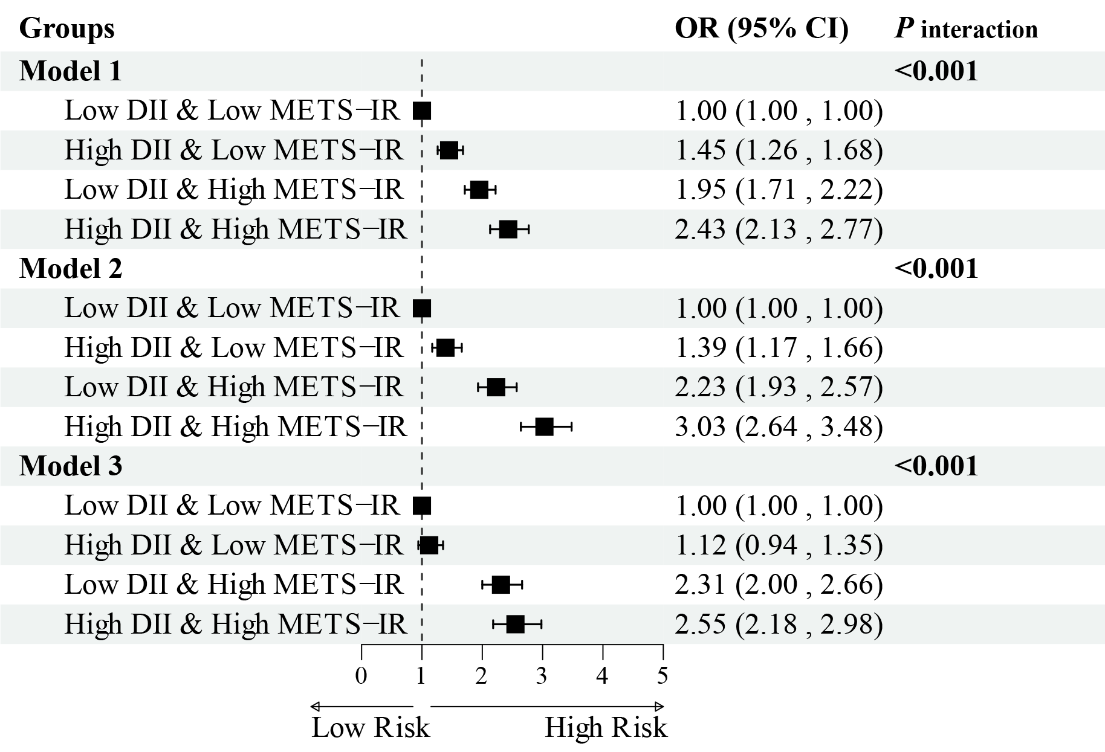


Participants are classified into four groups according to DII and TyG. DII is divided by median (0.99), and METS-IR is devided by inflection point identified in RCS analysis with CKM syndrome (42.18).

Advanced CKM stages was defined as Stage 3—4 (high-risk or established cardiovascular disease).

Model 1: unadjusted.

Model 2: adjusted for age, gender, and race.

Model 3: adjusted for age, gender, race, education, smoking status, physical activity, and total energy intake.

Abbreviations: DII, dietary inflammatory index; METS-IR, Metabolic Score for IR.

**Fig. S6. Weighted joint effects of dietary inflammatory index and HOMA-IR on advanced Cardiovascular-Kidney-Metabolic syndrome stages.**


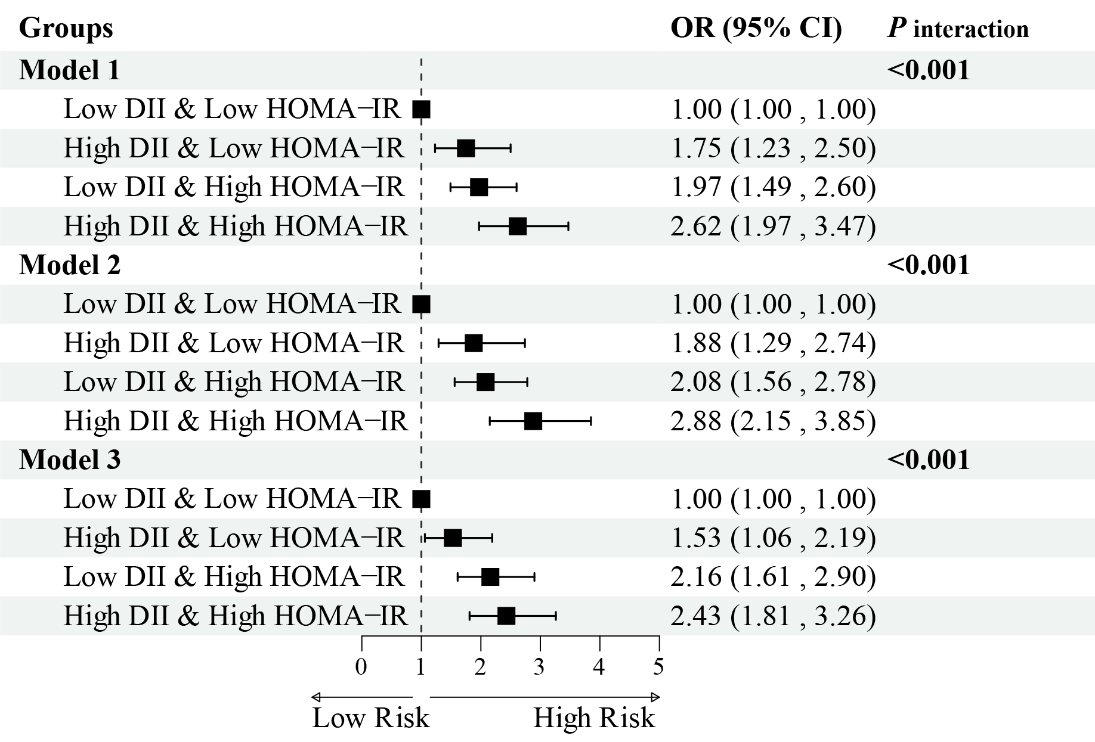


Participants are classified into four groups according to DII and TyG. DII is divided by median (0.99), and HOMA-IR is devided by inflection point identified in RCS analysis with CKM syndrome (1.33).

Advanced CKM stages was defined as Stage 3—4 (high-risk or established cardiovascular disease).

Model 1: unadjusted.

Model 2: adjusted for age, gender, and race.

Model 3: adjusted for age, gender, race, education, smoking status, physical activity, and total energy intake.

Abbreviations: DII, dietary inflammatory index; HOMA-IR, Homeostatic Model Assessment for IR.

**Fig.S7. Age subgroup analysis of the association between dietary inflammatory index and advanced Cardiovascular-Kidney-Metabolic syndrome stages.**


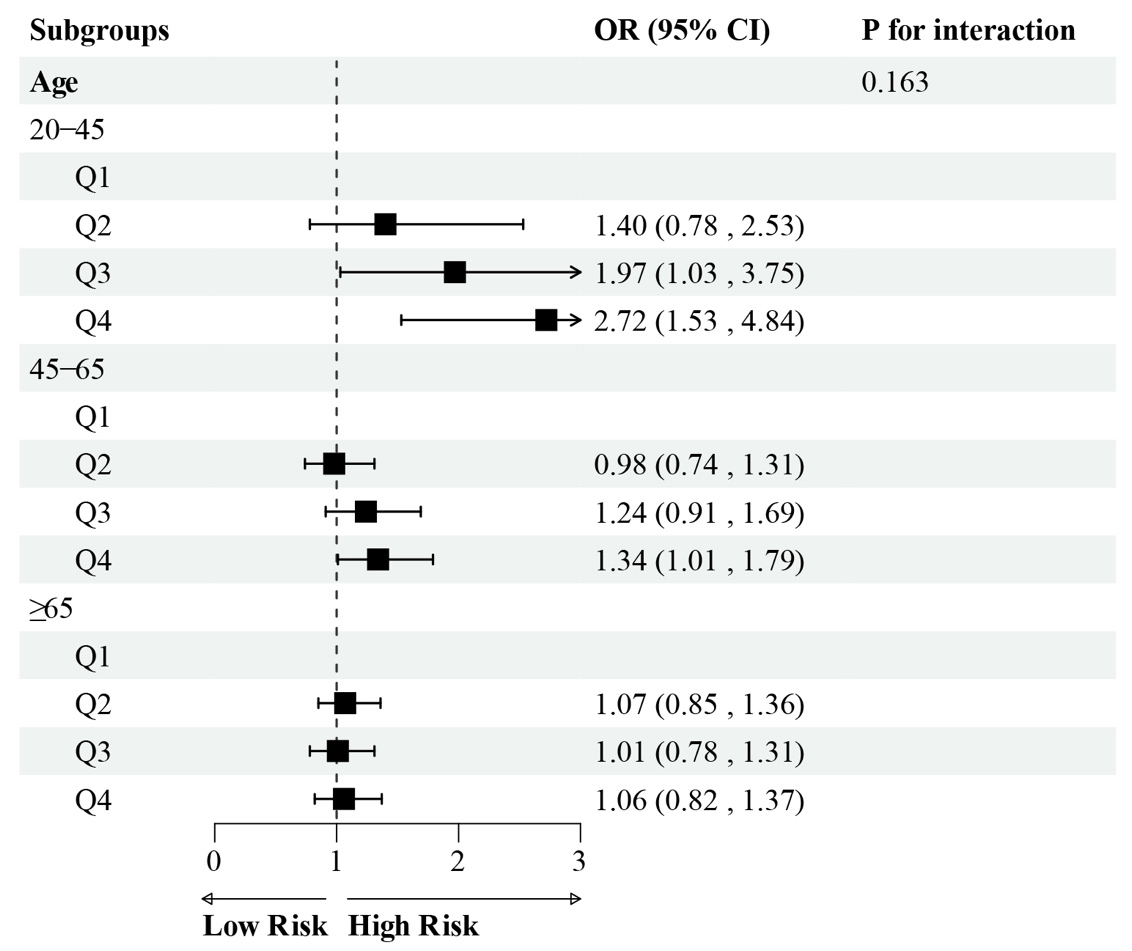


Advanced CKM stages was defined as Stage 3—4 (high-risk or established cardiovascular disease).

Adjusted for age, gender, race, education, smoking status, physical activity, and total energy intake.

**Fig.S8. Subgroup analysis of the association between dietary inflammatory index and advanced Cardiovascular-Kidney-Metabolic syndrome stages.**


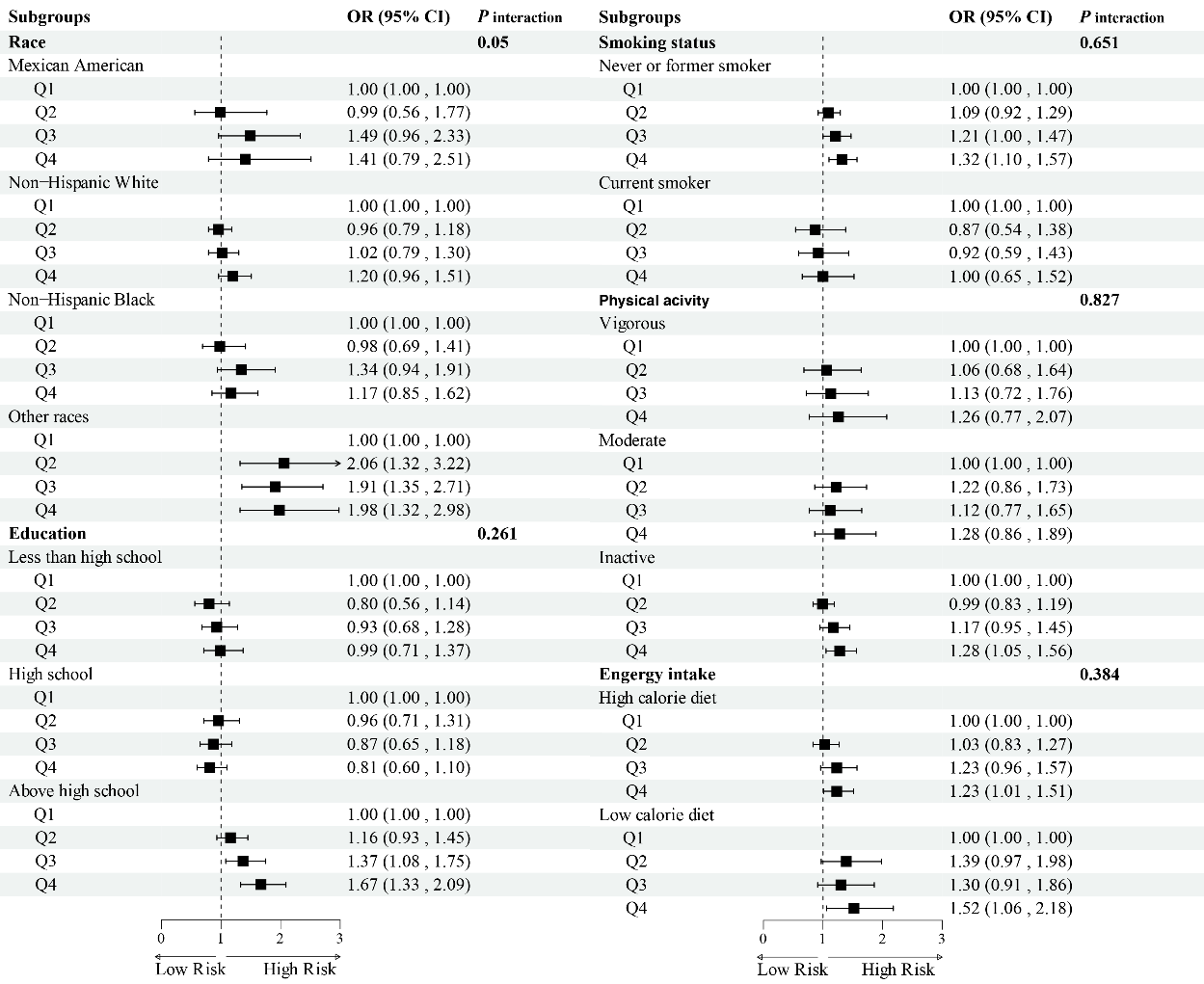


Advanced CKM stages was defined as Stage 3—4 (high-risk or established cardiovascular disease).

Adjusted for age, gender, race, education, smoking status, physical activity, and total energy intake.

**Table S17 Weighted baseline characteristics of participants according to Cardiovascular-Kidney-Metabolic syndrome stages (complete dataset).**

| Characteristic | Total  N = 12,993 | Cardiovascular–kidney–metabolic syndrome stage | | | | | *P* value |
| --- | --- | --- | --- | --- | --- | --- | --- |
|  |  | 0  N = 656 | 1  N = 2,718 | 2  N = 7,452 | 3  N = 643 | 4  N = 1,524 |  |
| Age, years | 48.77±16.87 | 34.71±13.87 | 40.48±14.57 | 49.37±14.96 | 73.53±8.87 | 64.87±12.81 | <0.001 |
| Male, % | 6,458 (49.37%) | 241 (36.56%) | 1,193 (45.88%) | 3,839 (51.56%) | 329 (48.14%) | 856 (53.74%) | <0.001 |
| Race, % |  |  |  |  |  |  | <0.001 |
| Mexican American | 2,010 (8.56%) | 44 (4.17%) | 508 (11.09%) | 1,254 (8.95%) | 59 (4.34%) | 145 (4.87%) |  |
| Non-Hispanic White | 5,475 (66.87%) | 297 (71.02%) | 1,032 (62.72%) | 2,991 (66.50%) | 326 (73.90%) | 829 (73.33%) |  |
| Non-Hispanic Black | 2,610 (10.67%) | 102 (8.67%) | 543 (11.23%) | 1,492 (10.37%) | 152 (12.47%) | 321 (11.78%) |  |
| Other races | 2,898 (13.90%) | 213 (16.14%) | 635 (14.95%) | 1,715 (14.18%) | 106 (9.29%) | 229 (10.02%) |  |
| Education, % |  |  |  |  |  |  | <0.001 |
| Less than high school | 3,230 (16.28%) | 80 (9.07%) | 542 (13.01%) | 1,909 (16.49%) | 193 (21.53%) | 506 (25.64%) |  |
| High school | 2,960 (23.57%) | 111 (16.86%) | 564 (22.60%) | 1,738 (23.95%) | 172 (28.60%) | 375 (26.18%) |  |
| Above high school | 6,803 (60.15%) | 465 (74.07%) | 1,612 (64.38%) | 3,805 (59.56%) | 278 (49.86%) | 643 (48.18%) |  |
| Current smoker, % | 10,400 (80.49%) | 539 (83.68%) | 2,203 (82.77%) | 5,906 (79.31%) | 564 (87.91%) | 1,188 (77.21%) | <0.001 |
| Physical activity, % |  |  |  |  |  |  | <0.001 |
| Vigorous | 2,623 (22.08%) | 133 (19.06%) | 621 (24.29%) | 1,588 (23.13%) | 64 (12.54%) | 217 (16.30%) |  |
| Moderate | 2,820 (24.60%) | 153 (24.26%) | 616 (24.48%) | 1,623 (25.33%) | 117 (21.46%) | 311 (22.00%) |  |
| Inactive | 7,550 (53.33%) | 370 (56.68%) | 1,481 (51.23%) | 4,241 (51.55%) | 462 (66.00%) | 996 (61.71%) |  |
| DII, /day | 0.87±2.04 | 0.54±2.09 | 0.73±2.07 | 0.88±2.01 | 1.17±2.01 | 1.21±2.05 | <0.001 |
| Energy intake, Kcal/day | 2,141.91±973.03 | 2,177.85±957.58 | 2,187.81±935.99 | 2,184.41±1,004.16 | 1,750.28±705.44 | 1,907.19±901.51 | <0.001 |
| TyG | 8.62±0.66 | 7.93±0.40 | 8.22±0.38 | 8.81±0.64 | 8.87±0.67 | 8.84±0.70 | <0.001 |
| METS-IR | 43.72±12.63 | 29.08±3.44 | 40.00±8.52 | 46.23±12.94 | 44.56±11.71 | 47.22±14.08 | <0.001 |
| HOMA-IR | 2.51 (1.51, 4.32) | 1.11 (0.78, 1.63) | 1.95 (1.36, 3.00) | 2.94 (1.74, 4.96) | 3.07 (1.76, 5.02) | 3.22 (1.80, 5.82) | <0.001 |

Participants with missing data on age, gender, race, education, smoking status, physical activity, total energy intake, TyG, METS-IR, and HOMA-IR is further excluded.

Data are presented as weighted mean ± standard deviation for normally distributed continuous variables, weighted median (interquartile range) for non-normally distributed continuous variables, or number (weighted percentage) for categorical variables.

Abbreviations: BMI: body mass index; WC, waist circumference; DII, dietary inflammatory index; TG: triglyceride; TC, total cholesterol; LDL, low-density lipoprotein cholesterol; HDL, high-density lipoprotein cholesterol; FBG, fasting blood glucose; Scr, serum creatinine; eGFR, estimated glomerular filtration rate; UACR, urinary albumin creatinine ratio; CKD, chronic kidney disease; CVD, cardiovascular disease; TyG, triglyceride–glucose index; METS-IR, Metabolic Score for IR; HOMA-IR, Homeostatic Model Assessment for IR.

**Table 18** **Weighted logistic regression analysis for the association** **between dietary inflammatory index and** **Cardiovascular-Kidney-Metabolic syndrome stages** **(complete dataset).**

|  | DII | Stage 1 | Stage 2 | Stage 3 | Stage 4 | Advanced stages |
| --- | --- | --- | --- | --- | --- | --- |
| Model 1 | Continuous | 1.04 (0.98, 1.11) | 1.09 (1.02, 1.15) | 1.16 (1.07, 1.26) | 1.17 (1.08, 1.26) | 1.10 (1.06, 1.14) |
|  | Q1 | Reference | Reference | Reference | Reference | Reference |
|  | Q2 | 0.92 (0.61, 1.39) | 1.05 (0.73, 1.51) | 1.28 (0.81, 2.02) | 1.03 (0.71, 1.50) | 1.09 (0.86, 1.37) |
|  | Q3 | 1.08 (0.77, 1.50) | 1.18 (0.86, 1.62) | 1.26 (0.81, 1.96) | 1.49 (1.04, 2.13) | 1.26 (0.99, 1.60) |
|  | Q4 | 1.12 (0.80, 1.56) | 1.39 (1.00, 1.94) | 2.13 (1.34, 3.37) | 2.00 (1.30, 3.07) | 1.59 (1.26, 2.00) |
|  | *P* for trend | 0.361 | 0.035 | 0.003 | <0.001 | <0.001 |
| Model 2 | Continuous | 1.11 (1.04, 1.18) | 1.18 (1.11, 1.26) | 1.31 (1.14, 1.52) | 1.33 (1.20, 1.48) | 1.11 (1.06, 1.16) |
|  | Q1 | Reference | Reference | Reference | Reference | Reference |
|  | Q2 | 1.05 (0.68, 1.60) | 1.18 (0.78, 1.79) | 1.07 (0.43, 2.69) | 1.25 (0.71, 2.21) | 1.11 (0.86, 1.43) |
|  | Q3 | 1.34 (0.95, 1.90) | 1.54 (1.12, 2.14) | 1.07 (0.43, 2.67) | 2.57 (1.43, 4.62) | 1.29 (1.00, 1.68) |
|  | Q4 | 1.49 (1.03, 2.13) | 2.12 (1.48, 3.03) | 3.20 (1.17, 8.74) | 3.54 (1.99, 6.32) | 1.65 (1.28, 2.13) |
|  | *P* for trend | 0.014 | <0.001 | 0.028 | <0.001 | <0.001 |
| Model 3 | Continuous | 1.12 (1.04, 1.20) | 1.20 (1.13, 1.28) | 1.41 (1.17, 1.69) | 1.30 (1.16, 1.45) | 1.08 (1.03, 1.13) |
|  | Q1 | Reference | Reference | Reference | Reference | Reference |
|  | Q2 | 1.11 (0.75, 1.64) | 1.36 (0.95, 1.96) | 1.44 (0.57, 3.66) | 1.36 (0.76, 2.43) | 1.10 (0.86, 1.40) |
|  | Q3 | 1.37 (0.99, 1.88) | 1.69 (1.25, 2.30) | 1.36 (0.56, 3.32) | 2.13 (1.19, 3.80) | 1.12 (0.85, 1.48) |
|  | Q4 | 1.72 (1.17, 2.53) | 2.63 (1.82, 3.80) | 3.97 (1.16, 13.6) | 3.21 (1.71, 6.04) | 1.43 (1.12, 1.83) |
|  | *P* for trend | 0.003 | <0.001 | 0.025 | <0.001 | <0.001 |

Data are presented as odds ratio (95% confidence interval). Advanced CKM stages was defined as Stage 3—4 (high-risk or established cardiovascular disease).

Model 1: unadjusted.

Model 2: adjusted for age, gender, and race.

Model 3: adjusted for age, gender, race, education, smoking status, physical activity, and total energy intake.

**Table S19** **Mediation effects of TyG between dietary inflammatory index and Cardiovascular-Kidney-Metabolic syndrome stages (complete dataset).**

| **Model 1** | | | | | |
| --- | --- | --- | --- | --- | --- |
| **Exposure to Mediator (a: DII → TyG)** | | | **Indirect Effect (a*b: DII → TyG → CKM stages)** | | |
| $\beta$ (SE) | *P* value | Standardized $\beta$ | $\beta$ (95% CI) | *P* value | Standardized $\beta$ |
| 0.008 (0.003) | 0.004 | 0.025 | 0.006 (0.002, 0.01) | 0.005 | 0.010 |
| **Mediator to Outcome (b: TyG → CKM stages)** | | | **Total Effect (c+a*b: DII → CKM stages)** | | |
| $\beta$ (SE) | *P* value | Standardized $\beta$ | $\beta$ (95% CI) | *P* value | Standardized $\beta$ |
| 0.670 (0.013) | <0.001 | 0.414 | 0.052 (0.042, 0.062) | <0.001 | 0.096 |
| **Direct Effect (c: DII** $\frac{\boldsymbol{\times}\mathbf{TyG}}{\boldsymbol{\to}}$ **CKM stages)** | | | **Proportion Mediated (a*b/c+a*b)** | | |
| $\beta$ (SE) | *P* value | Standardized $\beta$ | 10.8% |  |  |
| 0.047 (0.005) | <0.001 | 0.086 |  |  |  |
| **Model 2** |  |  |  |  |  |
| **Exposure to Mediator (a: DII → TyG)** | | | **Indirect Effect (a*b: DII → TyG → CKM stages)** | | |
| $\beta$ (SE) | *P* value | Standardized $\beta$ | $\beta$ (95% CI) | *P* value | Standardized $\beta$ |
| 0.017 (0.003) | <0.001 | 0.051 | 0.010 (0.007, 0.014) | <0.001 | 0.016 |
| **Mediator to Outcome (b: TyG → CKM stages)** | | | **Total Effect (c+a*b: DII → CKM stages)** | | |
| $\beta$ (SE) | *P* value | Standardized $\beta$ | $\beta$ (95% CI) | *P* value | Standardized $\beta$ |
| 0.600 (0.014) | <0.001 | 0.310 | 0.070 (0.060, 0.080) | <0.001 | 0.107 |
| **Direct Effect (c: DII** $\frac{\boldsymbol{\times}\mathbf{TyG}}{\boldsymbol{\to}}$ **CKM stages)** | | | **Proportion Mediated (a*b/c+a*b)** | | |
| $\beta$ (SE) | *P* value | Standardized $\beta$ | 14.8% |  |  |
| 0.060 (0.005) | <0.001 | 0.091 |  |  |  |
| **Model 3** |  |  |  |  |  |
| **Exposure to Mediator (a: DII → TyG)** | | | **Indirect Effect (a*b: DII → TyG → CKM stages)** | | |
| $\beta$ (SE) | *P* value | Standardized $\beta$ | $\beta$ (95% CI) | *P* value | Standardized $\beta$ |
| 0.016 (0.003) | <0.001 | 0.047 | 0.009 (0.005, 0.013) | <0.001 | 0.014 |
| **Mediator to Outcome (b: TyG → CKM stages)** | | | **Total Effect (c+a*b: DII → CKM stages)** | | |
| $\beta$ (SE) | *P* value | Standardized $\beta$ | $\beta$ (95% CI) | *P* value | Standardized $\beta$ |
| 0.592 (0.014) | <0.001 | 0.304 | 0.060 (0.048, 0.072) | <0.001 | 0.091 |
| **Direct Effect (c: DII** $\frac{\boldsymbol{\times}\mathbf{TyG}}{\boldsymbol{\to}}$ **CKM stages)** | | | **Proportion Mediated (a*b/c+a*b)** | | |
| $\beta$ (SE) | *P* value | Standardized $\beta$ | 15.5% |  |  |
| 0.051 (0.006) | <0.001 | 0.077 |  |  |  |

Model 1: unadjusted.

Model 2: adjusted for age, gender, and race.

Model 3: adjusted for age, gender, race, education, smoking status, physical activity, and total energy intake.

Abbreviations: DII, dietary inflammatory index; CKM, Cardiovascular-Kidney-Metabolic; TyG, triglyceride–glucose index.

**Table S20 Mediation effects of METS-IR between dietary inflammatory index and Cardiovascular-Kidney-Metabolic syndrome stages (complete dataset).**

| **Model 1** | | | | | |
| --- | --- | --- | --- | --- | --- |
| **Exposure to Mediator (a: DII → METS-IR)** | | | **Indirect Effect (a*b: DII → METS-IR → CKM stages)** | | |
| $\beta$ (SE) | *P* value | Standardized $\beta$ | $\beta$ (95% CI) | *P* value | Standardized $\beta$ |
| 0.414 (0.054) | <0.001 | 0.067 | 0.011 (0.008, 0.014) | <0.001 | 0.021 |
| **Mediator to Outcome (b: METS-IR → CKM stages)** | | | **Total Effect (c+a*b: DII → CKM stages)** | | |
| $\beta$ (SE) | *P* value | Standardized $\beta$ | $\beta$ (95% CI) | *P* value | Standardized $\beta$ |
| 0.026 (0.001) | <0.001 | 0.313 | 0.050 (0.041, 0.060) | <0.001 | 0.096 |
| **Direct Effect (c: DII** $\frac{\boldsymbol{\times}\mathbf{METS-IR}}{\boldsymbol{\to}}$ **CKM stages)** | | | **Proportion Mediated (a*b/c+a*b)** | | |
| $\beta$ (SE) | *P* value | Standardized $\beta$ | 21.7% |  |  |
| 0.039 (0.005) | <0.001 | 0.075 |  |  |  |
| **Model 2** |  |  |  |  |  |
| **Exposure to Mediator (a: DII →METS-IR)** | | | **Indirect Effect (a*b: DII → METS-IR → CKM stages)** | | |
| $\beta$ (SE) | *P* value | Standardized $\beta$ | $\beta$ (95% CI) | *P* value | Standardized $\beta$ |
| 0.491 (0.055) | <0.001 | 0.079 | 0.016 (0.012, 0.019) | <0.001 | 0.024 |
| **Mediator to Outcome (b: METS-IR → CKM stages)** | | | **Total Effect (c+a*b: DII → CKM stages)** | | |
| $\beta$ (SE) | *P* value | Standardized $\beta$ | $\beta$ (95% CI) | *P* value | Standardized $\beta$ |
| 0.032 (0.001) | <0.001 | 0.305 | 0.070 (0.060, 0.081) | <0.001 | 0.107 |
| **Direct Effect (c: DII** $\frac{\boldsymbol{\times}\mathbf{METS-IR}}{\boldsymbol{\to}}$ **CKM stages)** | | | **Proportion Mediated (a*b/c+a*b)** | | |
| $\beta$ (SE) | *P* value | Standardized $\beta$ | 22.5% |  |  |
| 0.054 (0.005) | <0.001 | 0.083 |  |  |  |
| **Model 3** |  |  |  |  |  |
| **Exposure to Mediator (a: DII →METS-IR)** | | | **Indirect Effect (a*b: DII → METS-IR → CKM stages)** | | |
| $\beta$ (SE) | *P* value | Standardized $\beta$ | $\beta$ (95% CI) | *P* value | Standardized $\beta$ |
| 0.677 (0.063) | <0.001 | 0.109 | 0.022 (0.018, 0.026) | <0.001 | 0.034 |
| **Mediator to Outcome (b: METS-IR → CKM stages)** | | | **Total Effect (c+a*b: DII → CKM stages)** | | |
| $\beta$ (SE) | *P* value | Standardized $\beta$ | $\beta$ (95% CI) | *P* value | Standardized $\beta$ |
| 0.033 (0.001) | <0.001 | 0.308 | 0.060 (0.048, 0.072) | <0.001 | 0.091 |
| **Direct Effect (c: DII** $\frac{\boldsymbol{\times}\mathbf{METS-IR}}{\boldsymbol{\to}}$ **CKM stages)** | | | **Proportion Mediated (a*b/c+a*b)** | | |
| $\beta$ (SE) | *P* value | Standardized $\beta$ | 36.7% |  |  |
| 0.038 (0.006) | <0.001 | 0.058 |  |  |  |

Model 1: unadjusted.

Model 2: adjusted for age, gender, and race.

Model 3: adjusted for age, gender, race, education, smoking status, physical activity, and total energy intake.

Abbreviations: DII, dietary inflammatory index; CKM, Cardiovascular-Kidney-Metabolic; METS-IR, Metabolic Score for IR.

**Table S21 Mediation effects of HOMA-IR between dietary inflammatory index and Cardiovascular-Kidney-Metabolic syndrome stages (complete dataset).**

| **Model 1** | | | | | |
| --- | --- | --- | --- | --- | --- |
| **Exposure to Mediator (a: DII → HOMA-IR)** | | | **Indirect Effect (a*b: DII → HOMA-IR → CKM stages)** | | |
| $\beta$ (SE) | *P* value | Standardized $\beta$ | $\beta$ (95% CI) | *P* value | Standardized $\beta$ |
| 0.076 (0.036) | 0.036 | 0.020 | 0.004 (0.000, 0.008) | 0.036 | 0.007 |
| **Mediator to Outcome (b: HOMA-IR → CKM stages)** | | | **Total Effect (c+a*b: DII → CKM stages)** | | |
| $\beta$ (SE) | *P* value | Standardized $\beta$ | $\beta$ (95% CI) | *P* value | Standardized $\beta$ |
| 0.052 (0.000) | <0.001 | 0.363 | 0.051 (0.041, 0.061) | <0.001 | 0.096 |
| **Direct Effect (c: DII** $\frac{\boldsymbol{\times}\mathbf{HOMA-IR}}{\boldsymbol{\to}}$ **CKM stages)** | | | **Proportion Mediated (a*b/c+a*b)** | | |
| $\beta$ (SE) | *P* value | Standardized $\beta$ | 7.7% |  |  |
| 0.047 (0.004) | <0.001 | 0.089 |  |  |  |
| **Model 2** |  |  |  |  |  |
| **Exposure to Mediator (a: DII →HOMA-IR)** | | | **Indirect Effect (a*b: DII → HOMA-IR → CKM stages)** | | |
| $\beta$ (SE) | *P* value | Standardized $\beta$ | $\beta$ (95% CI) | *P* value | Standardized $\beta$ |
| 0.103 (0.041) | 0.013 | 0.028 | 0.006 (0.001, 0.011) | 0.013 | 0.009 |
| **Mediator to Outcome (b: HOMA-IR → CKM stages)** | | | **Total Effect (c+a*b: DII → CKM stages)** | | |
| $\beta$ (SE) | *P* value | Standardized $\beta$ | $\beta$ (95% CI) | *P* value | Standardized $\beta$ |
| 0.060 (0.000) | <0.001 | 0.336 | 0.071 (0.061, 0.082) | <0.001 | 0.107 |
| **Direct Effect (c: DII** $\frac{\boldsymbol{\times}\mathbf{HOMA-IR}}{\boldsymbol{\to}}$ **CKM stages)** | | | **Proportion Mediated (a*b/c+a*b)** | | |
| $\beta$ (SE) | *P* value | Standardized $\beta$ | 8.7% |  |  |
| 0.065 (0.006) | <0.001 | 0.098 |  |  |  |
| **Model 3** |  |  |  |  |  |
| **Exposure to Mediator (a: DII → HOMA-IR)** | | | **Indirect Effect (a*b: DII → HOMA-IR → CKM stages)** | | |
| $\beta$ (SE) | *P* value | Standardized $\beta$ | $\beta$ (95% CI) | *P* value | Standardized $\beta$ |
| 0.157 (0.051) | 0.002 | 0.042 | 0.011 (0.004, 0.017) | 0.002 | 0.016 |
| **Mediator to Outcome (b: HOMA-IR → CKM stages)** | | | **Total Effect (c+a*b: DII → CKM stages)** | | |
| $\beta$ (SE) | *P* value | Standardized $\beta$ | $\beta$ (95% CI) | *P* value | Standardized $\beta$ |
| 0.068 (0.000) | <0.001 | 0.368 | 0.063 (0.050, 0.075) | <0.001 | 0.091 |
| **Direct Effect (c: DII** $\frac{\boldsymbol{\times}\mathbf{HOMA-IR}}{\boldsymbol{\to}}$ **CKM stages)** | | | **Proportion Mediated (a*b/c+a*b)** | | |
| $\beta$ (SE) | *P* value | Standardized $\beta$ | 17.7% |  |  |
| 0.052 (0.007) | <0.001 | 0.076 |  |  |  |

Model 1: unadjusted.

Model 2: adjusted for age, gender, and race.

Model 3: adjusted for age, gender, race, education, smoking status, physical activity, and total energy intake.

Abbreviations: DII, dietary inflammatory index; CKM, Cardiovascular-Kidney-Metabolic; HOMA-IR, Homeostatic Model Assessment for IR.

**Table S22 Weighted baseline characteristics of participants according to Cardiovascular-Kidney-Metabolic syndrome stages** **(Dietary inflammatory index recalculated dataset).**

| Characteristic | Total  N = 20,325 | Cardiovascular–kidney–metabolic syndrome stage | | | | | *P* value |
| --- | --- | --- | --- | --- | --- | --- | --- |
|  |  | 0  N = 1,177 | 1  N = 4,751 | 2  N = 10,785 | 3  N = 1,059 | 4  N = 2,553 |  |
| Age, years | 48.54±16.86 | 36.29±13.83 | 40.27±14.10 | 49.81±15.01 | 72.70±9.50 | 65.11±12.78 | <0.001 |
| Male, % | 9,844 (48.71%) | 489 (40.80%) | 2,064 (46.39%) | 5,310 (50.20%) | 518 (46.95%) | 1,463 (54.28%) | <0.001 |
| Race, % |  |  |  |  |  |  | <0.001 |
| Mexican American | 2,973 (8.80%) | 99 (6.34%) | 885 (11.82%) | 1,646 (8.62%) | 106 (4.39%) | 237 (5.09%) |  |
| Non-Hispanic White | 8,843 (67.17%) | 543 (71.22%) | 1,889 (63.71%) | 4,438 (66.43%) | 567 (76.78%) | 1,406 (73.65%) |  |
| Non-Hispanic Black | 4,267 (10.83%) | 181 (7.36%) | 880 (10.51%) | 2,400 (11.30%) | 245 (11.41%) | 561 (11.82%) |  |
| Other races | 4,242 (13.20%) | 354 (15.07%) | 1,097 (13.96%) | 2,301 (13.66%) | 141 (7.42%) | 349 (9.43%) |  |
| Education, % |  |  |  |  |  |  | <0.001 |
| Less than high school | 4,697 (14.77%) | 129 (7.76%) | 937 (13.46%) | 2,520 (14.51%) | 311 (20.78%) | 800 (23.04%) |  |
| High school | 4,677 (23.22%) | 221 (17.03%) | 994 (21.85%) | 2,539 (23.99%) | 266 (24.69%) | 657 (27.34%) |  |
| Above high school | 10,951 (62.01%) | 827 (75.21%) | 2,820 (64.70%) | 5,726 (61.49%) | 482 (54.54%) | 1,096 (49.62%) |  |
| Current smoker, % | 16,416 (81.39%) | 943 (82.01%) | 3,837 (81.25%) | 8,706 (81.30%) | 914 (88.22%) | 2,016 (79.13%) | 0.019 |
| Physical activity, % |  |  |  |  |  |  | <0.001 |
| Vigorous | 3,988 (22.08%) | 246 (21.22%) | 1,068 (23.22%) | 2,224 (23.77%) | 111 (9.75%) | 339 (15.53%) |  |
| Moderate | 4,442 (24.73%) | 285 (26.59%) | 1,057 (25.23%) | 2,385 (24.69%) | 215 (22.48%) | 500 (22.96%) |  |
| Inactive | 11,895 (53.19%) | 646 (52.19%) | 2,626 (51.55%) | 6,176 (51.55%) | 733 (67.77%) | 1,714 (61.51%) |  |
| DII, /day | 0.91±1.96 | 0.54±1.99 | 0.82±1.99 | 0.94±1.94 | 1.05±1.86 | 1.23±1.94 | <0.001 |
| Energy intake, Kcal/day | 2,117.67±936.67 | 2,177.66±1,059.75 | 2,169.63±916.54 | 2,144.89±945.06 | 1,838.14±702.76 | 1,890.56±867.24 | <0.001 |
| TyG | 8.68±0.52 | 8.10±0.32 | 8.39±0.30 | 8.82±0.48 | 8.99±0.55 | 9.06±0.58 | <0.001 |
| METS-IR | 44.10±12.41 | 29.75±3.60 | 42.53±9.15 | 46.13±12.88 | 46.21±12.46 | 48.51±13.54 | <0.001 |
| HOMA-IR | 3.01 (2.08, 4.12) | 1.67 (1.10, 2.16) | 2.70 (1.96, 3.31) | 3.33 (2.39, 4.62) | 3.46 (2.43, 7.94) | 4.12 (2.87, 8.48) | <0.001 |

DII and total energy intake are recalculated based on the mean intake from two 24-hour dietary recalls.

Participants with missing information on DII or energy intake in the second day of 24-hour dietary recalls and with a two-day DII level difference exceeding the interquartile range were further excluded.

Data are presented as weighted mean ± standard deviation for normally distributed continuous variables, weighted median (interquartile range) for non-normally distributed continuous variables, or number (weighted percentage) for categorical variables.

Abbreviations: BMI: body mass index; WC, waist circumference; DII, dietary inflammatory index; TG: triglyceride; TC, total cholesterol; LDL, low-density lipoprotein cholesterol; HDL, high-density lipoprotein cholesterol; FBG, fasting blood glucose; Scr, serum creatinine; eGFR, estimated glomerular filtration rate; UACR, urinary albumin creatinine ratio; CKD, chronic kidney disease; CVD, cardiovascular disease;

TyG, triglyceride–glucose index; METS-IR, Metabolic Score for IR; HOMA-IR, Homeostatic Model Assessment for IR.

**Table S23** **Weighted logistic regression analysis for the association between dietary inflammatory index and Cardiovascular-Kidney-Metabolic syndrome stages** **(Dietary inflammatory index recalculated dataset).**

|  | DII | Stage 1 | Stage 2 | Stage 3 | Stage 4 | Advanced stages |
| --- | --- | --- | --- | --- | --- | --- |
| Model 1 | Continuous |  |  |  |  |  |
|  | Q1 | Reference | Reference | Reference | Reference | Reference |
|  | Q2 | 1.16 (0.92, 1.47) | 1.41 (1.09, 1.81) | 1.58 (1.15, 2.18) | 1.58 (1.19, 2.09) | 1.23 (1.03, 1.47) |
|  | Q3 | 1.20 (0.94, 1.52) | 1.32 (1.06, 1.65) | 1.38 (0.93, 2.03) | 1.60 (1.24, 2.06) | 1.23 (1.03, 1.47) |
|  | Q4 | 1.38 (1.11, 1.71) | 1.67 (1.33, 2.11) | 1.97 (1.33, 2.93) | 2.39 (1.77, 3.23) | 1.51 (1.28, 1.78) |
|  | *P* for trend | 0.004 | <0.001 | 0.005 | <0.001 | <0.001 |
| Model 2 | Continuous |  |  |  |  |  |
|  | Q1 | Reference | Reference | Reference | Reference | Reference |
|  | Q2 | 1.24 (0.96, 1.59) | 1.55 (1.15, 2.09) | 1.97 (0.94, 4.13) | 2.94 (1.81, 4.78) | 1.29 (1.07, 1.56) |
|  | Q3 | 1.37 (1.06, 1.77) | 1.69 (1.30, 2.19) | 3.04 (1.47, 6.28) | 4.10 (2.60, 6.46) | 1.36 (1.11, 1.67) |
|  | Q4 | 1.62 (1.25, 2.10) | 2.31 (1.74, 3.07) | 4.12 (1.79, 9.46) | 6.10 (3.69, 10.1) | 1.65 (1.39, 1.97) |
|  | *P* for trend | <0.001 | <0.001 | 0.028 | <0.001 | <0.001 |
| Model 3 | Continuous |  |  |  |  |  |
|  | Q1 | Reference | Reference | Reference | Reference | Reference |
|  | Q2 | 1.19 (0.92, 1.55) | 1.53 (1.13, 2.07) | 1.79 (0.90, 3.56) | 2.79 (1.74, 4.47) | 1.20 (0.99, 1.46) |
|  | Q3 | 1.28 (0.96, 1.71) | 1.60 (1.19, 2.15) | 2.31 (1.04, 5.14) | 3.48 (2.11, 5.75) | 1.16 (0.93, 1.43) |
|  | Q4 | 1.43 (1.07, 1.92) | 2.12 (1.57, 2.88) | 3.01 (1.21, 7.52) | 4.72 (2.63, 8.50) | 1.29 (1.04, 1.61) |
|  | *P* for trend | 0.019 | <0.001 | 0.020 | <0.001 | 0.046 |

DII and total energy intake are recalculated based on the mean intake from two 24-hour dietary recalls.

Data are presented as odds ratio (95% confidence interval). Advanced CKM stages was defined as Stage 3—4 (high-risk or established cardiovascular disease).

Model 1: unadjusted.

Model 2: adjusted for age, gender, and race.

Model 3: adjusted for age, gender, race, education, smoking status, physical activity, and total energy intake.

**Table S24** **Mediation effects of TyG between dietary inflammatory index and Cardiovascular-Kidney-Metabolic syndrome stages (Dietary inflammatory index recalculated dataset).**

| **Model 1** | | | | | |
| --- | --- | --- | --- | --- | --- |
| **Exposure to Mediator (a: DII → TyG)** | | | **Indirect Effect (a*b: DII → TyG → CKM stages)** | | |
| $\beta$ (SE) | *P* value | Standardized $\beta$ | $\beta$ (95% CI) | *P* value | Standardized $\beta$ |
| 0.014 (0.002) | <0.001 | 0.046 | 0.016 (0.011, 0.021) | <0.001 | 0.025 |
| **Mediator to Outcome (b: TyG → CKM stages)** | | | **Total Effect (c+a*b: DII → CKM stages)** | | |
| $\beta$ (SE) | *P* value | Standardized $\beta$ | $\beta$ (95% CI) | *P* value | Standardized $\beta$ |
| 1.177 (0.009) | <0.001 | 0.542 | 0.071 (0.061, 0.08) | <0.001 | 0.108 |
| **Direct Effect (c: DII** $\frac{\boldsymbol{\times}\mathbf{TyG}}{\boldsymbol{\to}}$ **CKM stages)** | | | **Proportion Mediated (a*b/c+a*b)** | | |
| $\beta$ (SE) | *P* value | Standardized $\beta$ | 23.0% |  |  |
| 0.054 (0.004) | <0.001 | 0.083 |  |  |  |
| **Model 2** |  |  |  |  |  |
| **Exposure to Mediator (a: DII → TyG)** | | | **Indirect Effect (a*b: DII → TyG → CKM stages)** | | |
| $\beta$ (SE) | *P* value | Standardized $\beta$ | $\beta$ (95% CI) | *P* value | Standardized $\beta$ |
| 0.024 (0.002) | <0.001 | 0.078 | 0.026 (0.021, 0.03) | <0.001 | 0.033 |
| **Mediator to Outcome (b: TyG → CKM stages)** | | | **Total Effect (c+a*b: DII → CKM stages)** | | |
| $\beta$ (SE) | *P* value | Standardized $\beta$ | $\beta$ (95% CI) | *P* value | Standardized $\beta$ |
| 1.101 (0.010) | <0.001 | 0.422 | 0.098 (0.088, 0.108) | <0.001 | 0.124 |
| **Direct Effect (c: DII** $\frac{\boldsymbol{\times}\mathbf{TyG}}{\boldsymbol{\to}}$ **CKM stages)** | | | **Proportion Mediated (a*b/c+a*b)** | | |
| $\beta$ (SE) | *P* value | Standardized $\beta$ | 26.5% |  |  |
| 0.072 (0.005) | <0.001 | 0.091 |  |  |  |
| **Model 3** |  |  |  |  |  |
| **Exposure to Mediator (a: DII → TyG)** | | | **Indirect Effect (a*b: DII → TyG → CKM stages)** | | |
| $\beta$ (SE) | *P* value | Standardized $\beta$ | $\beta$ (95% CI) | *P* value | Standardized $\beta$ |
| 0.026 (0.002) | <0.001 | 0.087 | 0.027 (0.022, 0.032) | <0.001 | 0.035 |
| **Mediator to Outcome (b: TyG → CKM stages)** | | | **Total Effect (c+a*b: DII → CKM stages)** | | |
| $\beta$ (SE) | *P* value | Standardized $\beta$ | $\beta$ (95% CI) | *P* value | Standardized $\beta$ |
| 1.042 (0.011) | <0.001 | 0.404 | 0.084 (0.073, 0.095) | <0.001 | 0.108 |
| **Direct Effect (c: DII** $\frac{\boldsymbol{\times}\mathbf{TyG}}{\boldsymbol{\to}}$ **CKM stages)** | | | **Proportion Mediated (a*b/c+a*b)** | | |
| $\beta$ (SE) | *P* value | Standardized $\beta$ | 32.3% |  |  |
| 0.057 (0.005) | <0.001 | 0.073 |  |  |  |

DII and total energy intake are recalculated based on the mean intake from two 24-hour dietary recalls.

Model 1: unadjusted.

Model 2: adjusted for age, gender, and race.

Model 3: adjusted for age, gender, race, education, smoking status, physical activity, and total energy intake.

Abbreviations: DII, dietary inflammatory index; CKM, Cardiovascular-Kidney-Metabolic; TyG, triglyceride–glucose index.

**Table S25 Mediation effects of METS-IR between dietary inflammatory index and Cardiovascular-Kidney-Metabolic syndrome stages (Dietary inflammatory index recalculated dataset).**

| **Model 1** | | | | | |
| --- | --- | --- | --- | --- | --- |
| **Exposure to Mediator (a: DII → METS-IR)** | | | **Indirect Effect (a*b: DII → METS-IR → CKM stages)** | | |
| $\beta$ (SE) | *P* value | Standardized $\beta$ | $\beta$ (95% CI) | *P* value | Standardized $\beta$ |
| 0.672 (0.049) | <0.001 | 0.098 | 0.016 (0.014, 0.019) | <0.001 | 0.029 |
| **Mediator to Outcome (b: METS-IR → CKM stages)** | | | **Total Effect (c+a*b: DII → CKM stages)** | | |
| $\beta$ (SE) | *P* value | Standardized $\beta$ | $\beta$ (95% CI) | *P* value | Standardized $\beta$ |
| 0.025 (0.001) | <0.001 | 0.292 | 0.062 (0.054, 0.07) | <0.001 | 0.108 |
| **Direct Effect (c: DII** $\frac{\boldsymbol{\times}\mathbf{METS-IR}}{\boldsymbol{\to}}$ **CKM stages)** | | | **Proportion Mediated (a*b/c+a*b)** | | |
| $\beta$ (SE) | *P* value | Standardized $\beta$ | 26.6% |  |  |
| 0.045 (0.004) | <0.001 | 0.079 |  |  |  |
| **Model 2** |  |  |  |  |  |
| **Exposure to Mediator (a: DII →METS-IR)** | | | **Indirect Effect (a*b: DII → METS-IR → CKM stages)** | | |
| $\beta$ (SE) | *P* value | Standardized $\beta$ | $\beta$ (95% CI) | *P* value | Standardized $\beta$ |
| 0.775 (0.050) | <0.001 | 0.113 | 0.024 (0.02, 0.027) | <0.001 | 0.032 |
| **Mediator to Outcome (b: METS-IR → CKM stages)** | | | **Total Effect (c+a*b: DII → CKM stages)** | | |
| $\beta$ (SE) | *P* value | Standardized $\beta$ | $\beta$ (95% CI) | *P* value | Standardized $\beta$ |
| 0.030 (0.001) | <0.001 | 0.286 | 0.090 (0.081, 0.099) | <0.001 | 0.124 |
| **Direct Effect (c: DII** $\frac{\boldsymbol{\times}\mathbf{METS-IR}}{\boldsymbol{\to}}$ **CKM stages)** | | | **Proportion Mediated (a*b/c+a*b)** | | |
| $\beta$ (SE) | *P* value | Standardized $\beta$ | 26.1% |  |  |
| 0.067 (0.004) | <0.001 | 0.092 |  |  |  |
| **Model 3** |  |  |  |  |  |
| **Exposure to Mediator (a: DII →METS-IR)** | | | **Indirect Effect (a*b: DII → METS-IR → CKM stages)** | | |
| $\beta$ (SE) | *P* value | Standardized $\beta$ | $\beta$ (95% CI) | *P* value | Standardized $\beta$ |
| 1.023 (0.056) | <0.001 | 0.149 | 0.033 (0.029, 0.037) | <0.001 | 0.045 |
| **Mediator to Outcome (b: METS-IR → CKM stages)** | | | **Total Effect (c+a*b: DII → CKM stages)** | | |
| $\beta$ (SE) | *P* value | Standardized $\beta$ | $\beta$ (95% CI) | *P* value | Standardized $\beta$ |
| 0.032 (0.001) | <0.001 | 0.302 | 0.080 (0.069, 0.09) | <0.001 | 0.108 |
| **Direct Effect (c: DII** $\frac{\boldsymbol{\times}\mathbf{METS-IR}}{\boldsymbol{\to}}$ **CKM stages)** | | | **Proportion Mediated (a*b/c+a*b)** | | |
| $\beta$ (SE) | *P* value | Standardized $\beta$ | 41.6% |  |  |
| 0.047 (0.005) | <0.001 | 0.063 |  |  |  |

DII and total energy intake are recalculated based on the mean intake from two 24-hour dietary recalls.

Model 1: unadjusted.

Model 2: adjusted for age, gender, and race.

Model 3: adjusted for age, gender, race, education, smoking status, physical activity, and total energy intake.

Abbreviations: DII, dietary inflammatory index; CKM, Cardiovascular-Kidney-Metabolic; METS-IR, Metabolic Score for IR.

**Table S26 Mediation effects of HOMA-IR between dietary inflammatory index and Cardiovascular-Kidney-Metabolic syndrome stages (Dietary inflammatory index recalculated dataset).**

| **Model 1** | | | | | |
| --- | --- | --- | --- | --- | --- |
| **Exposure to Mediator (a: DII → HOMA-IR)** | | | **Indirect Effect (a*b: DII → HOMA-IR → CKM stages)** | | |
| $\beta$ (SE) | *P* value | Standardized $\beta$ | $\beta$ (95% CI) | *P* value | Standardized $\beta$ |
| 0.137 (0.024) | <0.001 | 0.044 | 0.013 (0.009, 0.018) | <0.001 | 0.021 |
| **Mediator to Outcome (b: HOMA-IR → CKM stages)** | | | **Total Effect (c+a*b: DII → CKM stages)** | | |
| $\beta$ (SE) | *P* value | Standardized $\beta$ | $\beta$ (95% CI) | *P* value | Standardized $\beta$ |
| 0.096 (0.001) | <0.001 | 0.473 | 0.067 (0.058, 0.076) | <0.001 | 0.108 |
| **Direct Effect (c: DII** $\frac{\boldsymbol{\times}\mathbf{HOMA-IR}}{\boldsymbol{\to}}$ **CKM stages)** | | | **Proportion Mediated (a*b/c+a*b)** | | |
| $\beta$ (SE) | *P* value | Standardized $\beta$ | 19.5% |  |  |
| 0.054 (0.005) | <0.001 | 0.087 |  |  |  |
| **Model 2** |  |  |  |  |  |
| **Exposure to Mediator (a: DII →HOMA-IR)** | | | **Indirect Effect (a*b: DII → HOMA-IR → CKM stages)** | | |
| $\beta$ (SE) | *P* value | Standardized $\beta$ | $\beta$ (95% CI) | *P* value | Standardized $\beta$ |
| 0.016 (0.003) | <0.001 | 0.047 | 0.009 (0.005, 0.013) | <0.001 | 0.014 |
| **Mediator to Outcome (b: HOMA-IR → CKM stages)** | | | **Total Effect (c+a*b: DII → CKM stages)** | | |
| $\beta$ (SE) | *P* value | Standardized $\beta$ | $\beta$ (95% CI) | *P* value | Standardized $\beta$ |
| 0.592 (0.014) | <0.001 | 0.304 | 0.060 (0.048, 0.072) | <0.001 | 0.091 |
| **Direct Effect (c: DII** $\frac{\boldsymbol{\times}\mathbf{HOMA-IR}}{\boldsymbol{\to}}$ **CKM stages)** | | | **Proportion Mediated (a*b/c+a*b)** | | |
| $\beta$ (SE) | *P* value | Standardized $\beta$ | 15.5% |  |  |
| 0.051 (0.006) | <0.001 | 0.077 |  |  |  |
| **Model 3** |  |  |  |  |  |
| **Exposure to Mediator (a: DII → HOMA-IR)** | | | **Indirect Effect (a*b: DII → HOMA-IR → CKM stages)** | | |
| $\beta$ (SE) | *P* value | Standardized $\beta$ | $\beta$ (95% CI) | *P* value | Standardized $\beta$ |
| 0.208 (0.032) | <0.001 | 0.068 | 0.021 (0.015, 0.027) | <0.001 | 0.027 |
| **Mediator to Outcome (b: HOMA-IR → CKM stages)** | | | **Total Effect (c+a*b: DII → CKM stages)** | | |
| $\beta$ (SE) | *P* value | Standardized $\beta$ | $\beta$ (95% CI) | *P* value | Standardized $\beta$ |
| 0.101 (0.001) | <0.001 | 0.395 | 0.085 (0.074, 0.096) | <0.001 | 0.108 |
| **Direct Effect (c: DII** $\frac{\boldsymbol{\times}\mathbf{HOMA-IR}}{\boldsymbol{\to}}$ **CKM stages)** | | | **Proportion Mediated (a*b/c+a*b)** | | |
| $\beta$ (SE) | *P* value | Standardized $\beta$ | 24.7% |  |  |
| 0.064 (0.006) | <0.001 | 0.082 |  |  |  |

DII and total energy intake are recalculated based on the mean intake from two 24-hour dietary recalls.

Model 1: unadjusted.

Model 2: adjusted for age, gender, and race.

Model 3: adjusted for age, gender, race, education, smoking status, physical activity, and total energy intake.

Abbreviations: DII, dietary inflammatory index; CKM, Cardiovascular-Kidney-Metabolic; HOMA-IR, Homeostatic Model Assessment for IR.

**Table S27 Weighted logistic regression analysis for the association between dietary inflammatory index and Redefined Advanced Cardiovascular-Kidney-Metabolic syndrome stages (Excluding Stage 0 participants).**

|  | DII | Advanced stages |
| --- | --- | --- |
| Model 1 | Continuous | 1.09 (1.06-1.12) |
|  | Q1 | Reference |
|  | Q2 | 1.13 (0.95-1.34) |
|  | Q3 | 1.30 (1.10-1.54) |
|  | Q4 | 1.53 (1.33-1.77) |
|  | *P* for trend | <0.001 |
| Model 2 | Continuous | 1.11 (1.08-1.14) |
|  | Q1 | Reference |
|  | Q2 | 1.15 (0.96-1.38) |
|  | Q3 | 1.36 (1.12-1.64) |
|  | Q4 | 1.67 (1.43-1.95) |
|  | *P* for trend | <0.001 |
| Model 3 | Continuous | 1.05 (1.02-1.08) |
|  | Q1 | Reference |
|  | Q2 | 1.04 (0.87-1.25) |
|  | Q3 | 1.13 (0.93-1.37) |
|  | Q4 | 1.24 (1.04-1.48) |
|  | *P* for trend | 0.014 |

Data are presented as odds ratio (95% confidence interval). Non-advanced CKM stages was defined as Stage 1—2, advanced CKM stages was defined as Stage 3—4.

Model 1: unadjusted.

Model 2: adjusted for age, gender, and race.

Model 3: adjusted for age, gender, race, education, smoking status, physical activity, and total energy intake.

**Table S28 Mediation effects of IR surrogates between dietary inflammatory index and Redefined Advanced Cardiovascular-Kidney-Metabolic syndrome stages (Excluding Stage 0 participants).**

| **TyG** | | | | | |
| --- | --- | --- | --- | --- | --- |
| **Exposure to Mediator (a: DII → TyG)** | | | **Indirect Effect (a*b: DII → TyG →advanced CKM stages** | | |
| $\beta$ (SE) | *P* value | Standardized $\beta$ | $\beta$ (95% CI) | *P* value | Standardized $\beta$ |
| 0.015 (0.002) | <0.001 | 0.054 | 0.009 (0.007-0.012) | <0.001 | 0.013 |
| **Mediator to Outcome (b: TyG →** **advanced CKM stages)** | | | **Total Effect (c+a*b: DII →advanced CKM stages)** | | |
| $\beta$ (SE) | *P* value | Standardized $\beta$ | $\beta$ (95% CI) | *P* value | Standardized $\beta$ |
| 0.650 (0.016) | <0.001 | 0.245 | 0.048 (0.035-0.061) | <0.001 | 0.068 |
| **Direct Effect (c: DII** $\frac{\boldsymbol{\times}\mathbf{TyG}}{\boldsymbol{\to}}$ **advanced CKM stages)** | | | **Proportion Mediated (a*b/c+a*b)** | | |
| $\beta$ (SE) | *P* value | Standardized $\beta$ | 19.7% |  |  |
| 0.039 (0.006) | <0.001 | 0.054 |  |  |  |
| **METS-IR** |  |  |  |  |  |
| **Exposure to Mediator (a: DII →METS-IR)** | | | **Indirect Effect (a*b: DII →METS-IR →advanced CKM stages)** | | |
| $\beta$ (SE) | *P* value | Standardized $\beta$ | $\beta$ (95% CI) | *P* value | Standardized $\beta$ |
| 0.731 (0.043) | <0.001 | 0.121 | 0.015 (0.012-0.017) | <0.001 | 0.021 |
| **Mediator to Outcome (b: METS-IR →advanced CKM stages)** | | | **Total Effect (c+a*b: DII →advanced CKM stages)** | | |
| $\beta$ (SE) | *P* value | Standardized $\beta$ | $\beta$ (95% CI) | *P* value | Standardized $\beta$ |
| 0.020 (0.001) | <0.001 | 0.173 | 0.047 (0.034-0.059) | <0.001 | 0.068 |
| **Direct Effect (c: DII** $\frac{\boldsymbol{\times}\boldsymbol{METS-IR}}{\boldsymbol{\to}}$ **advanced CKM stages)** | | | **Proportion Mediated (a*b/c+a*b)** | | |
| $\beta$ (SE) | *P* value | Standardized $\beta$ | 31.0% |  |  |
| 0.032 (0.006) | <0.001 | 0.047 |  |  |  |
| **HOMA-IR** |  |  |  |  |  |
| **Exposure to Mediator (a: DII →HOMA-IR)** | | | **Indirect Effect (a*b: DII →HOMA-IR →advanced CKM stages)** | | |
| $\beta$ (SE) | *P* value | Standardized $\beta$ | $\beta$ (95% CI) | *P* value | Standardized $\beta$ |
| 0.133 (0.026) | <0.001 | 0.048 | 0.235 (0.144-0.325) | <0.001 | 0.035 |
| **Mediator to Outcome (b: HOMA-IR →advanced CKM stages)** | | | **Total Effect (c+a*b: DII →advanced CKM stages)** | | |
| $\beta$ (SE) | *P* value | Standardized $\beta$ | $\beta$ (95% CI) | *P* value | Standardized $\beta$ |
| 1.762 (0.021) | <0.001 | 0.732 | 0.456 (0.335-0.577) | <0.001 | 0.068 |
| **Direct Effect (c: DII** $\frac{\boldsymbol{\times}\boldsymbol{HOMA-IR}}{\boldsymbol{\to}}$ **advanced CKM stages)** | | | **Proportion Mediated (a*b/c+a*b)** | | |
| $\beta$ (SE) | *P* value | Standardized $\beta$ | 51.5% |  |  |
| 0.221 (0.070) | 0.002 | 0.033 |  |  |  |

Non-advanced CKM stages was defined as Stage 1—2, advanced CKM stages was defined as Stage 3—4.

Adjusted for age, gender, race, education, smoking status, physical activity, and total energy intake.

Abbreviations: DII, dietary inflammatory index; CKM, Cardiovascular-Kidney-Metabolic; TyG, triglyceride–glucose index; METS-IR, Metabolic Score for Insulin Resistance; HOMA-IR, Homeostatic Model Assessment for Insulin Resistance.

**Table S29 Weighted logistic regression analysis for the association between dietary inflammatory index and Cardiovascular-Kidney-Metabolic syndrome stages (****Excluding participants with** **prior genetic conditions).**

|  | DII | Stage 1 | Stage 2 | Stage 3 | Stage 4 | Advanced stages |
| --- | --- | --- | --- | --- | --- | --- |
| Model 1 | Continuous | 1.08 (1.04-1.12) | 1.10 (1.06-1.13) | 1.13 (1.08-1.19) | 1.19 (1.14-1.25) | 1.09 (1.06-1.13) |
|  | Q1 | Reference | Reference | Reference | Reference | Reference |
|  | Q2 | 1.09 (0.88-1.35) | 1.28 (1.02-1.59) | 1.38 (0.95-1.99) | 1.36 (1.08-1.71) | 1.15 (0.95-1.40) |
|  | Q3 | 1.24 (0.99-1.56) | 1.32 (1.07-1.64) | 1.33 (0.93-1.89) | 1.75 (1.35-2.27) | 1.30 (1.07-1.57) |
|  | Q4 | 1.42 (1.17-1.72) | 1.55 (1.30-1.86) | 1.77 (1.30-2.41) | 2.46 (1.91-3.16) | 1.56 (1.33-1.84) |
|  | *P* for trend | <0.001 | <0.001 | 0.002 | <0.001 | <0.001 |
| Model 2 | Continuous | 1.12 (1.08-1.16) | 1.15 (1.11-1.19) | 1.34 (1.17-1.52) | 1.36 (1.26-1.46) | 1.12 (1.09-1.16) |
|  | Q1 | Reference | Reference | Reference | Reference | Reference |
|  | Q2 | 1.17 (0.94-1.45) | 1.33 (1.03-1.71) | 1.65 (0.76-3.56) | 1.69 (1.15-2.50) | 1.17 (0.96-1.43) |
|  | Q3 | 1.43 (1.13-1.81) | 1.58 (1.24-2.01) | 2.68 (1.32-5.45) | 3.43 (2.22-5.29) | 1.42 (1.15-1.76) |
|  | Q4 | 1.72 (1.39-2.13) | 2.02 (1.63-2.51) | 3.67 (1.89-7.13) | 4.88 (3.32-7.16) | 1.78 (1.50-2.11) |
|  | *P* for trend | <0.001 | <0.001 | <0.001 | <0.001 | <0.001 |
| Model 3 | Continuous | 1.12 (1.07-1.18) | 1.17 (1.12-1.23) | 1.24 (1.05-1.45) | 1.35 (1.23-1.48) | 1.06 (1.02-1.10) |
|  | Q1 | Reference | Reference | Reference | Reference | Reference |
|  | Q2 | 1.16 (0.94-1.45) | 1.36 (1.05-1.77) | 1.43 (0.65-3.12) | 1.67 (1.14-2.43) | 1.05 (0.86-1.27) |
|  | Q3 | 1.42 (1.10-1.84) | 1.64 (1.26-2.14) | 1.89 (0.89-4.03) | 3.14 (1.97-5.01) | 1.16 (0.93-1.44) |
|  | Q4 | 1.68 (1.31-2.16) | 2.12 (1.64-2.75) | 2.40 (1.10-5.24) | 4.28 (2.69-6.81) | 1.29 (1.06-1.55) |
|  | *P* for trend | <0.001 | <0.001 | 0.029 | <0.001 | 0.008 |

Prior genetic conditions: Type 1 diabetes mellitus (a diagnosis of diabetes before 30 years of age with the use of insulin within one year of diabetes diagnosis) and familial hypercholesterolemia (LDL-C ≥190 mg/dL with a history of premature CVD in parents).

Data are presented as odds ratio (95% confidence interval). Advanced CKM stages was defined as Stage 3—4 (high-risk or established cardiovascular disease).

Model 1: unadjusted.

Model 2: adjusted for age, gender, and race.

Model 3: adjusted for age, gender, race, education, smoking status, physical activity, and total energy intake.

Abbreviations: DII, dietary inflammatory index; CKM, Cardiovascular-Kidney-Metabolic.

**Table S30 Mediation effects of IR surrogates between dietary inflammatory index and Redefined Advanced Cardiovascular-Kidney-Metabolic syndrome stages (Excluding participants with prior genetic conditions).**

| **TyG** | | | | | | | | |
| --- | --- | --- | --- | --- | --- | --- | --- | --- |
| **Exposure to Mediator (a: DII → TyG)** | | | | | **Indirect Effect (a*b: DII → TyG →CKM stages** | | | |
| $\beta$ (SE) | *P* value | | Standardized $\beta$ | | $\beta$ (95% CI) | | *P* value | Standardized $\beta$ |
| 0.019 (0.002) | <0.001 | | 0.068 | | 0.013 (0.010-0.015) | | <0.001 | 0.018 |
| **Mediator to Outcome (b: TyG →CKM stages)** | | | | | **Total Effect (c+a*b: DII →CKM stages)** | | | |
| $\beta$ (SE) | *P* value | | Standardized $\beta$ | | $\beta$ (95% CI) | | *P* value | Standardized $\beta$ |
| 0.677 (0.017) | <0.001 | | 0.273 | | 0.054 (0.040-0.068) | | <0.001 | 0.079 |
| **Direct Effect (c: DII** $\frac{\boldsymbol{\times}\mathbf{TyG}}{\boldsymbol{\to}}$ **CKM stages)** | | | | | **Proportion Mediated (a*b/c+a*b)** | | | |
| $\beta$ (SE) | *P* value | | Standardized $\beta$ | | 23.3% | |  |  |
| 0.041 (0.007) | <0.001 | | 0.061 | |  | |  |  |
| **METS-IR** |  | |  | |  | |  |  |
| **Exposure to Mediator (a: DII →METS-IR)** | | | | | **Indirect Effect (a*b: DII →METS-IR →CKM stages)** | | | |
| $\beta$ (SE) | *P* value | | Standardized $\beta$ | | $\beta$ (95% CI) | | *P* value | Standardized $\beta$ |
| 0.837 (0.045) | <0.001 | | 0.135 | | 0.018 (0.016-0.020) | | <0.001 | 0.027 |
| **Mediator to Outcome (b: METS-IR →CKM stages)** | | | | | **Total Effect (c+a*b: DII →CKM stages)** | | | |
| $\beta$ (SE) | *P* value | | Standardized $\beta$ | | $\beta$ (95% CI) | | *P* value | Standardized $\beta$ |
| 0.022 (0.001) | <0.001 | | 0.202 | | 0.053 (0.039-0.066) | | <0.001 | 0.079 |
| **Direct Effect (c: DII** $\frac{\boldsymbol{\times}\boldsymbol{METS-IR}}{\boldsymbol{\to}}$**CKM stages)** | | | | | **Proportion Mediated (a*b/c+a*b)** | | | |
| $\beta$ (SE) | *P* value | | Standardized $\beta$ | | 34.4% | |  |  |
| 0.035 (0.007) | <0.001 | | 0.052 | |  | |  |  |
| **HOMA-IR** |  | |  | |  | |  |  |
| **Exposure to Mediator (a: DII →HOMA-IR)** | | | | | **Indirect Effect (a*b: DII →HOMA-IR →advanced CKM stages)** | | | |
| $\beta$ (SE) | *P* value | | Standardized $\beta$ | | $\beta$ (95% CI) | | *P* value | Standardized $\beta$ |
| 0.155 (0.026) | <0.001 | | 0.056 | | 0.277 (0.185-0.368) | | <0.001 | 0.043 |
| **Mediator to Outcome (b: HOMA-IR →CKM stages)** | | | | | **Total Effect (c+a*b: DII →CKM stages)** | | | |
| $\beta$ (SE) | *P* value | | Standardized $\beta$ | | $\beta$ (95% CI) | | *P* value | Standardized $\beta$ |
| 1.785 (0.031) | <0.001 | | 0.767 | | 0.509 (0.380-0.639) | | <0.001 | 0.079 |
| **Direct Effect (c: DII** $\frac{\boldsymbol{\times}\boldsymbol{HOMA-IR}}{\boldsymbol{\to}}$ **CKM stages)** | | | | | **Proportion Mediated (a*b/c+a*b)** | | | |
| $\beta$ (SE) | *P* value | | Standardized $\beta$ | | 54.4% | |  |  |
| 0.232 (0.073) | <0.001 | | 0.036 | |  | |  |  |
|  | *P* for trend | <0.001 | <0.001 | 0.029 | <0.001 | | 0.008 |  |

Prior genetic conditions: Type 1 diabetes mellitus (a diagnosis of diabetes before 30 years of age with the use of insulin within one year of diabetes diagnosis) and familial hypercholesterolemia (LDL-C ≥190 mg/dL with a history of premature CVD in parents).Adjusted for age, gender, race, education, smoking status, physical activity, and total energy intake.

Abbreviations: DII, dietary inflammatory index; CKM, Cardiovascular-Kidney-Metabolic; TyG, triglyceride–glucose index; METS-IR, Metabolic Score for Insulin Resistance; HOMA-IR, Homeostatic Model Assessment for Insulin Resistance.

**Table S31 Alternate** **Mediterranean Diet Index components and criteria for scoring.**

| Food Groups | Criteria for score 0 | Criteria for score 1 |
| --- | --- | --- |
| Whole Fruit | < Median | ≥ Median |
| Vegetables | < Median | ≥ Median |
| Whole grain products | < Median | ≥ Median |
| Legumes | < Median | ≥ Median |
| Nuts | < Median | ≥ Median |
| Fish | < Median | ≥ Median |
| Red and processed meat | < Median | ≥ Median |
| Ratio of monosat to sat lipids | < Median | ≥ Median |
| Alcohol | < 5 or >15 g/d for female;  < 5 or >25 g/d for male | 5-15 g/d for female;  5-25 g/d for male |
| Total | 0 | 9 |

The higher the alternate Mediterranean Diet Index (aMD) scores, the better of the adherence to the MD.

**Table S32 Weighted logistic regression analysis for the association between dietary inflammatory index and Cardiovascular-Kidney-Metabolic syndrome stages (****Further adjusting for aMD).**

| DII | Stage 1 | Stage 2 | Stage 3 | Stage 4 | Advanced stages |
| --- | --- | --- | --- | --- | --- |
| Continuous | 1.11 (1.06-1.18) | 1.16 (1.11-1.22) | 1.17 (1.00-1.40) | 1.37 (1.24-1.51) | 1.06 (1.02-1.10) |
| Q1 | Reference | Reference | Reference | Reference | Reference |
| Q2 | 1.13 (0.90-1.41) | 1.32 (1.02-1.73) | 1.28 (0.59-2.80) | 1.64 (1.12-2.40) | 1.05 (0.87-1.26) |
| Q3 | 1.34 (1.03-1.75) | 1.56 (1.19-2.04) | 1.57 (0.71-3.47) | 3.15 (1.91-5.20) | 1.05 (0.93-1.41) |
| Q4 | 1.56 (1.19-2.04) | 1.97 (1.49-2.62) | 1.98 (1.05-4.44) | 4.37 (2.59-7.37) | 1.26 (1.03-1.55) |
| *P* for trend | 0.001 | <0.001 | 0.048 | <0.001 | 0.021 |

Adjusted for age, gender, race, education, smoking status, physical activity, total energy intake, and aMD.

Abbreviations: DII, dietary inflammatory index; CKM, Cardiovascular-Kidney-Metabolic.

**Fig. S9. Subgroup analysis by aMD of the association between the DII and advanced CKM syndrome stages.**


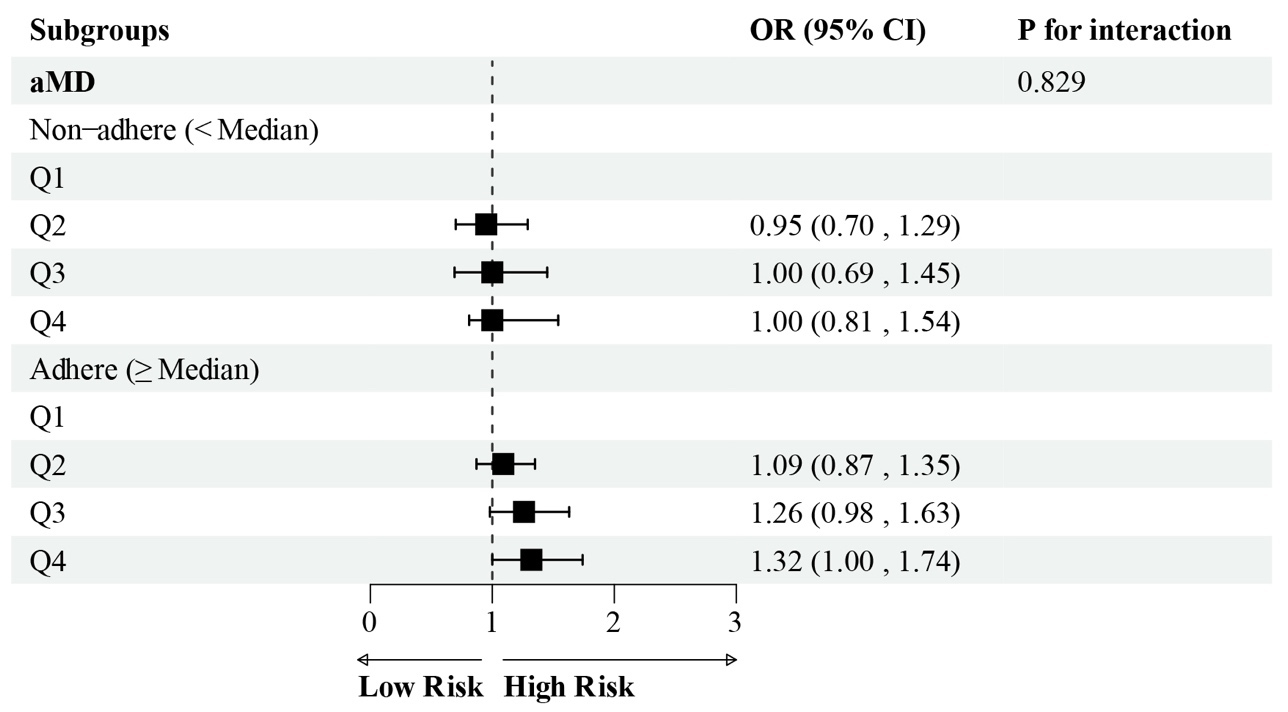


The aMD score was divided into two groups based on the median: < median as low adherence to the MD group, and ≥median as high adherence to the MD group.

Advanced CKM stages was defined as Stage 3—4 (high-risk or established cardiovascular disease).

Adjusted for age, gender, race, education, smoking status, physical activity, and total energy intake.

**Table S33 Mediation effects of IR surrogates between dietary inflammatory index and Redefined Advanced Cardiovascular-Kidney-Metabolic syndrome stages (Further adjusting for aMD).**

| **TyG** | | | | | |
| --- | --- | --- | --- | --- | --- |
| **Exposure to Mediator (a: DII → TyG)** | | | **Indirect Effect (a*b: DII → TyG →CKM stages** | | |
| $\beta$ (SE) | *P* value | Standardized $\beta$ | $\beta$ (95% CI) | *P* value | Standardized $\beta$ |
| 0.015 (0.002) | <0.001 | 0.053 | 0.015 (0.011-0.019) | <0.001 | 0.021 |
| **Mediator to Outcome (b: TyG →CKM stages)** | | | **Total Effect (c+a*b: DII →CKM stages)** | | |
| $\beta$ (SE) | *P* value | Standardized $\beta$ | $\beta$ (95% CI) | *P* value | Standardized $\beta$ |
| 1.033 (0.009) | <0.001 | 0.403 | 0.056 (0.047-0.065) | <0.001 | 0.080 |
| **Direct Effect (c: DII** $\frac{\boldsymbol{\times}\mathbf{TyG}}{\boldsymbol{\to}}$ **CKM stages)** | | | **Proportion Mediated (a*b/c+a*b)** | | |
| $\beta$ (SE) | *P* value | Standardized $\beta$ | 26.9% |  |  |
| 0.041 (0.004) | <0.001 | 0.058 |  |  |  |
| **METS-IR** |  |  |  |  |  |
| **Exposure to Mediator (a: DII →METS-IR)** | | | **Indirect Effect (a*b: DII →METS-IR →CKM stages)** | | |
| $\beta$ (SE) | *P* value | Standardized $\beta$ | $\beta$ (95% CI) | *P* value | Standardized $\beta$ |
| 0.663 (0.046) | <0.001 | 0.108 | 0.020 (0.018-0.023) | <0.001 | 0.031 |
| **Mediator to Outcome (b: METS-IR →CKM stages)** | | | **Total Effect (c+a*b: DII →CKM stages)** | | |
| $\beta$ (SE) | *P* value | Standardized $\beta$ | $\beta$ (95% CI) | *P* value | Standardized $\beta$ |
| 0.031 (0.001) | <0.001 | 0.288 | 0.052 (0.044-0.061) | <0.001 | 0.080 |
| **Direct Effect (c: DII** $\frac{\boldsymbol{\times}\boldsymbol{METS-IR}}{\boldsymbol{\to}}$**CKM stages)** | | | **Proportion Mediated (a*b/c+a*b)** | | |
| $\beta$ (SE) | *P* value | Standardized $\beta$ | 39.1% |  |  |
| 0.032 (0.004) | <0.001 | 0.049 |  |  |  |
| **HOMA-IR** |  |  |  |  |  |
| **Exposure to Mediator (a: DII →HOMA-IR)** | | | **Indirect Effect (a*b: DII →HOMA-IR →advanced CKM stages)** | | |
| $\beta$ (SE) | *P* value | Standardized $\beta$ | $\beta$ (95% CI) | *P* value | Standardized $\beta$ |
| 0.106 (0.026) | <0.001 | 0.039 | 0.011 (0.006-0.016) | <0.001 | 0.016 |
| **Mediator to Outcome (b: HOMA-IR →CKM stages)** | | | **Total Effect (c+a*b: DII →CKM stages)** | | |
| $\beta$ (SE) | *P* value | Standardized $\beta$ | $\beta$ (95% CI) | *P* value | Standardized $\beta$ |
| 0.105 (0.000) | <0.001 | 0.403 | 0.057 (0.047-0.066) | <0.001 | 0.080 |
| **Direct Effect (c: DII** $\frac{\boldsymbol{\times}\boldsymbol{HOMA-IR}}{\boldsymbol{\to}}$ **CKM stages)** | | | **Proportion Mediated (a*b/c+a*b)** | | |
| $\beta$ (SE) | *P* value | Standardized $\beta$ | 19.6% |  |  |
| 0.046 (0.005) | <0.001 | 0.064 |  |  |  |

Adjusted for age, gender, race, education, smoking status, physical activity, total energy intake, and aMD.

Abbreviations: DII, dietary inflammatory index; CKM, Cardiovascular-Kidney-Metabolic; TyG, triglyceride–glucose index; METS-IR, Metabolic Score for Insulin Resistance; HOMA-IR, Homeostatic Model Assessment for Insulin Resistance.
